# Effects of *CYP2C19* and *CYP2D6* gene variants on escitalopram and aripiprazole treatment outcome and serum levels: results from the CAN-BIND 1 study

**DOI:** 10.1038/s41398-022-02124-4

**Published:** 2022-09-06

**Authors:** Farhana Islam, Victoria S. Marshe, Leen Magarbeh, Benicio N. Frey, Roumen V. Milev, Claudio N. Soares, Sagar V. Parikh, Franca Placenza, Stephen C. Strother, Stefanie Hassel, Valerie H. Taylor, Francesco Leri, Pierre Blier, Rudolf Uher, Faranak Farzan, Raymond W. Lam, Gustavo Turecki, Jane A. Foster, Susan Rotzinger, Sidney H. Kennedy, Daniel J. Müller

**Affiliations:** 1 Campbell Family Mental Health Research Institute, Centre for Addiction and Mental Health, Toronto, ON Canada; 2 Department of Pharmacology & Toxicology, University of Toronto, Toronto, ON Canada; 3 Institute of Medical Science, University of Toronto, Toronto, ON Canada; 4 Department of Psychiatry and Behavioural Neurosciences, McMaster University, Hamilton, ON Canada; 5 St. Joseph’s Healthcare Hamilton, Hamilton, ON Canada; 6 Department of Psychiatry, Queen’s University, Providence Care, Kingston, ON Canada; 7 Department of Psychiatry, University of Michigan, Ann Arbor, MI USA; 8 Centre for Mental Health, University Health Network, Toronto, ON Canada; 9 Rotman Research Institute, Baycrest Medical Centre, and Medical Biophysics, University of Toronto, Toronto, ON Canada; 10 Department of Psychiatry, University of Calgary, Calgary, AB Canada; 11 Department of Psychology and Neuroscience, University of Guelph, Guelph, ON Canada; 12 The Royal Institute of Mental Health Research, Ottawa, ON Canada; 13 Department of Psychiatry, Dalhousie University, Halifax, NS Canada; 14 Mechatronic Systems Engineering, Simon Fraser University, Surrey, BC Canada; 15 University of British Columbia and Vancouver Coastal Health Authority, Vancouver, BC Canada; 16 McGill Group for Suicide Studies, Douglas Mental Health University Institute, McGill University, Verdun, QC Canada; 17 Department of Psychiatry, St Michael’s Hospital, University of Toronto, Toronto, ON Canada; 18 Department of Psychiatry, University of Toronto, Toronto, ON Canada; 19 Keenan Research Centre for Biomedical Science, Li Ka Shing Knowledge Institute, St Michael’s Hospital, Toronto, ON Canada; 20 Department of Psychiatry, Psychosomatics and Psychotherapy, Center of Mental Health, University Clinic of Würzburg, Würzburg, Germany

**Keywords:** Clinical genetics, Predictive markers, Depression, Pharmacogenomics, Personalized medicine

## Abstract

Cytochrome P450 drug-metabolizing enzymes may contribute to interindividual differences in antidepressant outcomes. We investigated the effects of *CYP2C19* and *CYP2D6* gene variants on response, tolerability, and serum concentrations. Patients (*N* = 178) were treated with escitalopram (ESC) from weeks 0–8 (Phase I), and at week 8, either continued ESC if they were responders or were augmented with aripiprazole (ARI) if they were non-responders (<50% reduction in Montgomery–Åsberg Depression Rating Scale from baseline) for weeks 8–16 (Phase II). Our results showed that amongst patients on ESC-Only, *CYP2C19* intermediate and poor metabolizers (IM + PMs), with reduced or null enzyme function, trended towards significantly lower symptom improvement during Phase II compared to normal metabolizers (NMs), which was not observed in ESC + ARI. We further showed that *CYP2D6* NMs and IM + PMs had a higher likelihood of reporting a treatment-related central nervous system side effect in ESC-Only and ESC + ARI, respectively. The differences in the findings between ESC-Only and ESC + ARI may be due to the altered pharmacokinetics of ESC by ARI coadministration in ESC + ARI. We provided evidence for this postulation when we showed that in ESC-Only, *CYP2C19* and *CYP2D6* IM + PMs demonstrated significantly higher ESC concentrations at Weeks 10 and 16 compared to NMs. In contrast, ESC + ARI showed an association with *CYP2C19* but not with *CYP2D6* metabolizer group. Instead, ESC + ARI showed an association between *CYP2D6* metabolizer group and ARI metabolite-to-drug ratio suggesting potential competition between ESC and ARI for CYP2D6. Our findings suggest that dosing based on *CYP2C19* and *CYP2D6* genotyping could improve safety and outcome in patients on ESC monotherapy.

## Introduction

Polymorphisms in genes encoding cytochrome P450 (CYP) enzymes, which mediate the Phase I metabolism of many antidepressants, result in variability in enzyme activity and contribute to large interindividual differences in drug metabolism [1, 2]. Hence, CYP450 genotyping has the potential to improve the efficacy and tolerability of antidepressants for the treatment of the major depressive disorder (MDD) by guiding medication selection and dosage adjustments according to the genetically predicted rate of drug metabolism of individual patients [3].

Escitalopram (ESC), the *S*-enantiomer of racemic citalopram, is one of the most effective and well-tolerated selective-serotonin reuptake inhibitors (SSRIs) prescribed for the treatment of MDD [4, 5]. ESC is biotransformed to its primary metabolite, *S*-desmethylcitalopram (S-DCT), by CYP2C19 and CYP3A4, with a minor role of CYP2D6 [6–8]. For patients who do not show symptom improvement with first-line antidepressant monotherapy, augmentation strategies with atypical antipsychotics have been reported to improve outcomes [9]. Aripiprazole (ARI), a second-generation antipsychotic, is an effective augmentation option with standard antidepressant therapy for MDD treatment [10]. ARI is predominantly metabolized by CYP2D6 into its active metabolite, dehydroaripiprazole (DHA).

*CYP2CD6* and *CYP2C19* genes are highly polymorphic with multiple allelic variants associated with altered enzymatic capacity, while variations in the *CYP3A4* gene are less common and have little impact on its enzymatic activity. The Clinical Pharmacogenetics Implementation Consortium (CPIC) has published guidelines on *CYP2D6* and *CYP2C19* phenotype prediction from genotypes [11]. Individuals can be classified into phenotypic subgroups based on inherited alleles associated with differing rates of drug metabolism, including normal metabolizers (NMs), intermediate metabolizers (IMs), poor metabolizers (PMs), and ultra-rapid metabolizers (UMs)[11]. For *CYP2C19*, there are three additional phenotypic subgroups according to CPIC, including rapid metabolizers (RMs), as well as “likely IM” and “likely PM” for decreased function *CYP2C19* alleles with limited data to characterize function [12, 13].

Previous studies have shown genetic variations in *CYP2C19* to be associated with ESC exposure, efficacy, and tolerability [14–23]. Additionally, genetic variations in *CYP2D6* have been shown to significantly affect serum concentration of ARI and the sum of ARI and DHA [24–26]. However, the effects of variants of these genes on ESC and ARI exposure, efficacy, and tolerability when the two medications are co-administered are not well understood [25, 26].

Using data from the well-characterized Canadian Biomarker Integration Network in Depression—Study 1 (CAN-BIND-1) in which patients with MDD were treated with ESC monotherapy or ESC with adjunctive ARI, we had three main objectives:

1. to examine the relationships between *CYP2C19* and *CYP2D6* metabolizer phenotypes, treatment response, and tolerability;
2. to replicate previous findings showing that *CYP2C19* and *CYP2D6* metabolizer phenotypes influence serum concentrations of ESC and ARI and their metabolite-to-drug ratio; and
3. to explore whether there is a relationship between serum concentrations with the response and side effects of treatment.

We hypothesized that, compared to the non-NM metabolizer groups, *CYP2C19* and *CYP2D6* NMs would (1) be predominant among responders, show greater symptom improvement, and experience less treatment-related side effects, as well as (2) demonstrate lower parent drug concentrations and higher drug-to-metabolite ratio. We further hypothesized that (3) differences in serum concentrations would be correlated with variability in treatment response and tolerability.

## Methods

A detailed description of the protocol for the Canadian Biomarker Integration Network for Depression (CAN-BIND-1) clinical trial has been published elsewhere [10, 27].

### Treatment protocol

The 16-week study protocol consisted of two phases following screening and baseline visits. During Phase I (Weeks 0–8), participants were treated with open-label ESC (10–20 mg/day, flexible dosage) for 8 weeks. At Week 8, participants were classified as “responders” or “non-responders” if they demonstrated Montgomery–Åsberg Depression Rating Scale (MADRS) reductions of ≥50% or <50% from baseline, respectively. During Phase II (Weeks 8–16), responders continued ESC, whereas non-responders to ESC were augmented with ARI (2–10 mg/d, flexible dosage) for the second eight weeks. Blood samples were collected on Weeks 2, 10, and 16 to measure medication levels and on Week 4 for pharmacogenetic analyses. The methods used for the quantification of serum levels of drug and metabolite concentrations is described in the Supplementary Methods.

### Clinical sample

The study sample consisted of 211 participants diagnosed with MDD according to the Diagnostic and Statistical Manual for Mental Disorders IV (DSM-IV-TR; American Psychiatric Association, 2000) and confirmed using the Mini International Neuropsychiatric Interview (MINI) with age ranging from 18 to 61 years. Participants were free of psychotropic medications for at least five half-lives prior to the start of the trial, had a depressive episode duration of ⩾3 months, a total score of ⩾24 in the Montgomery–Åsberg depression rating scale (MADRS) at the time of screening, and fluency in English to complete self-report questionnaires. The exclusion criteria included any other psychiatric diagnosis as the primary diagnosis. including Bipolar I or II, significant neurological disorders or head trauma, high suicidal risk, psychosis in the current episode, or substance dependence or abuse in the past six months.

### Measures

#### Montgomery-Åsberg depression rating scale (MADRS)

Depressive symptoms were assessed using MADRS every 2 weeks from Week 0 to 16. The primary outcomes for response were: (1) response status (responder versus non-responder) on the last visit of Phases I and II (i.e., Week 8 and 16), and (2) the percentage of symptom improvement across visits during Phases I and II. Remission status (remitter versus non-remitter) at the end of Phases I and II was a secondary outcome for a response. These response outcomes are described in the Supplementary Methods.

#### Toronto side effects scale (TSES)

The Toronto side effects scale (TSES), administered on Weeks 2, 4, 10, 12, and 16, is a clinical instrument designed to assess the frequency and severity of treatment-related side effects on 5-point Likert scales. The “intensity” score is derived by multiplying the frequency and severity of each side effect [28, 29]. The items assessed can be broadly categorized into the central nervous system (CNS), gastrointestinal (GI), and sexual side effects, as well as weight gain (Table S1)[28]. The primary outcomes for side effects were both dichotomous and continuous: (1) absence or presence of side effects within the four categories on the last visit of Phases I and II and (2) the intensity of each category of side effect across visits during each Phase.

### DNA isolation and genotyping

SNPs and haplotypes for *CYP2C19* and *CYP2D6* that are associated with altered metabolism and are common in the reference population (consisting of Europeans, African Americans, and East Asian ancestry) were included for genotyping. These SNPs cover >95% of the common alleles associated with altered metabolism. Genomic DNA was extracted from venous blood samples using a modified version of the FlexiGene DNA kit (QIAGEN, Hilden, Germany) and sent for genotyping at the CAMH Biobank and Molecular Core Facility (Centre for Addiction and Mental Health, Toronto, Canada). Genotyping was performed using standard TaqMan® Assays (Thermo Fisher Scientific, ON, Canada) according to the manufacturer’s protocol to assess alleles and copy number variants (CNVs) in CYP2D6 (*1, *2, *3, *4, *5, *6, *9, *10, *17, *29, *36, *41) and in *CYP2C19* (*1, *2, *3, *17) [30]. CNVs, including deletion (*5) and multiplications of *CYP2D6*, were assessed using a copy-number assay and CopyCaller Version 1.0 (Applied Biosystems, Burlington, ON, Canada). The overall phenotype for *CYP2D6* duplications was determined using the results from the SNP and CNV assays (e.g. genotype is reported as *1/*3 (xN) if SNP assays revealed *1 and *3 and CNV assay showed more than two copies of the *CYP2D6* gene for the same participant).

Genotyping results were reviewed by two laboratory staff blind to the clinical data. Predictions of *CYP2D6* and *CYP2C19* metabolizer phenotypes were based on the expected enzyme activity of the alleles as reported in CPIC guidelines for CYP450 genes (https://cpicpgx.org/guidelines/guideline-for-selective-serotonin-reuptake-inhibitors-and-cyp2d6-and-cyp2c19/). Table S2 summarizes the expected enzymatic activity for *CYP2C19* and *CYP2D6* alleles. The predicted metabolizer phenotype based on *CYP2C19* and *CYP2D6* genotype is reported in Table S3. Ten percent of the sample was re-genotyped for quality control. Subjects for whom genotype was ambiguous were retyped, and if this result remained ambiguous, data from the participants were excluded from further analyses. A genotype is determined to be ambiguous if the sample failed to amplify or it does not clearly cluster with one of the three genotype clusters visualized in the ABI ViiA7 RUO (Applied Biosystems) software post-amplification.

### Statistical analysis

All analyses were conducted using R Version 4.2.1 (R Foundation for Statistical Computing Platform, 2022) and RStudio Version 2022.02.3 (RStudio Inc, 2022). The normality of variables was tested using the Shapiro–Wilk test. Descriptive statistics for demographic and clinical characteristics by *CYP2C19* (NMs vs. IM + PMs vs. RM + UMs) and *CYP2D6* (NMs vs. IM + PMs) metabolizer groups were generated using the chi-squared or Fisher’s exact test for categorical variables and the Mann–Whitney *U* or Kruskal–Wallis tests for continuous variables, as appropriate. There were only two *CYP2D6* UMs (two in ESC-Only and zero in ESC + ARI) precluding the creation of a separate group, and therefore they were excluded from analyses.

Given the different metabolic pathways of ESC and ARI, ESC-Only and ESC + ARI treatment arms are analyzed separately for Phase II. For analyses involving ESC, *CYP2C19* and *CYP2D6* metabolizer groups were included as fixed effect independent variables, since both enzymes are involved in the metabolism of ESC [6]. All analyses were also adjusted for age, sex, ancestry, and recruitment site, with the latter, included as a random effect factor in the linear mixed-effects models. Post-hoc comparisons for trends between ungrouped metabolizer phenotypes (NMs, IMs, and PMs for *CYP2D6*, as well as RMs and UMs for *CYP2C19*) were conducted for significant associations.

Linear models were checked for assumptions of normality and non-linearity, and log transformation was applied for skewed, non-normally distributed data, where applicable. The effect size for linear models was calculated using Cohen’s $f^2 = \frac{{R^2AB - R^2A}}{{\left( {1 - R^2A} \right)}}$, where *B* is the variable of interest, *A* is the set of all other predictors, *R*^2^*AB* is the variance explained for a multiple regression model with all of the predictors, and *R*^2^*A* is the variance explained for a model without the predictor (*B*) for which we want to calculate a “local” effect size [31, 32]. The effect is considered small at 0.02, medium at 0.15, and large at 0.35 [33].

Due to heterogeneity in ancestry among study participants, all analyses were repeated in the largest ancestral group, Europeans, to control for population stratification [34]. False discovery rate (FDR) approach was used to control for multiple comparisons in the analysis of each subsample (i.e. total sample for Phase I and treatment arms for Phase II) with a significance threshold of *q* < 0.05 (two-tailed) [35, 36]. For post-hoc comparisons, *p* < 0.05 was considered significant.

#### Association of CYP2C19 and CYP2D6 metabolizer status with outcome measures

We assessed the dichotomous measures of response (responder vs. non-responder and remitter vs. non-remitter) and side effects (present vs. absent) for the last timepoint in Phases I and II using logistic regression models with total MADRS score at baseline included as a covariate. Given the availability of biweekly MADRS scores and multiple time points for TSES, continuous measures of response (percentage of symptom improvement) and side effects (intensity of each category of side effects) were assessed using linear mixed-effects models that included interactions of *CYP2C19* and *CYP2D6* metabolizer groups with timepoint, and recruitment site and individual as random effects variables.

#### Association of CYP2C19 and CYP2D6 metabolizer status with measures of drug exposure

Using linear regression models, we examined the effects of *CYP2C19* and *CYP2D6* metabolizer groups on ESC exposure using three serum measures: concentrations of ESC, its primary metabolite, S-DCT, and the S-DCT/ESC ratio. Serum concentrations were adjusted for dosage (i.e., ng/mL/mg). Sampling time (i.e., hours since the last dose) was entered as a covariate in the regression models to account for when the medication was taken.

In the ESC + ARI treatment arm during Phase II, we also examined the effects of *CYP2D6* metabolizer group on dose-adjusted serum concentrations of ARI, its primary metabolite, DHA, and the DHA/ARI ratio using linear regression models, as described above. The *CYP2C19* metabolizer group was not included as a fixed effect covariate, as CYP2C19 is not known to be involved in the metabolism of ARI.

#### Associations of drug exposure with outcome measures

Using Spearman’s rank correlation, we explored whether measures of unadjusted serum concentrations were associated with symptom improvement and the intensity of side effects during Phase I and II.

## Results

### Sample demographics

**Fig. 1:**
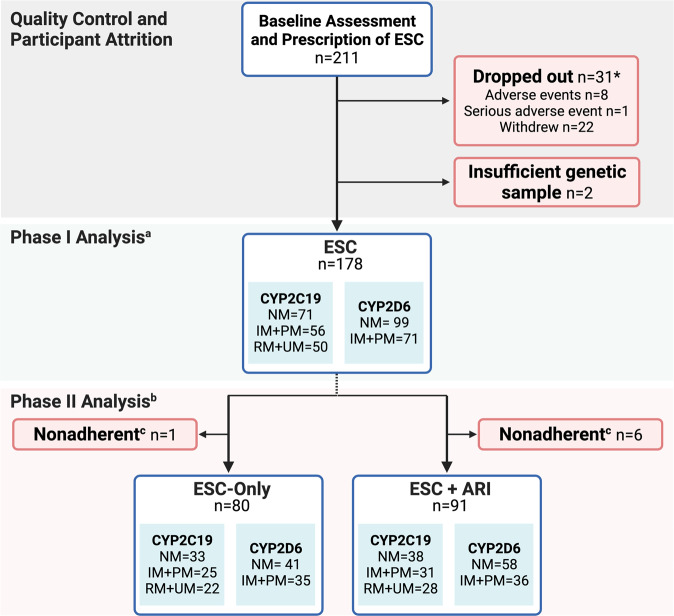
Process of research design. **a** During Phase I (Weeks 0–8), all participants received open-label ESC monotherapy (10–20 mg/d, flexible dosage). On Week 8, participants were classified as responders (≥50% decrease from baseline in MADRS scores) or non-responders (<50% decrease from baseline in MADRS scores). **b** During Phase II (Weeks 8–16), responders continued ESC monotherapy, while non-responders received ARI (2–10 mg/d, flexible dosage) augmentation in addition to ESC. **c** Adherence to the study medication was confirmed in participants based on the detection of ESC in serum at Weeks 2, 10, and 16, and the detection of ARI in serum at Weeks 10 and 16 for the ESC + ARI treatment arm. All participants were adherent to treatment during Phase I based on serum levels of the drug at Week 2. During Phase II, seven participants were non-adherent determined by a lack of treatment medication detected in serum at both Weeks 10 and 16, therefore they were not included in the Phase II analyses. ARI aripiprazole, ESC escitalopram, IM intermediate metabolizer, MADRS Montgomery–Åsberg Depression Rating Scale, NM normal metabolizer, PM poor metabolizer, RM rapid metabolizer, UM ultra-rapid metabolizer. *For details on the CAN-BIND 1 study protocol and a description of the sample, see ref. [27].

Participant flow is detailed in Fig. 1. We excluded 31 participants who dropped out prior to Week 8 and therefore lacked MADRS scores and drug serum levels for Phases I and II [27]. Amongst these dropouts, chi-square goodness-of-fit tests show that there were 10 NMs, 13 IM + PMs (11 IM, 2 PM), and 8 RM + UMs (7 RM, 1 UM) for *CYP2C19* (*χ*^2^(2) = 1.23, *p* = 0.542), and 18 NMs and 12 IM + PMs (6 IM, 0 PM) for *CYP2D6* (*χ*^2^(1) = 1.20, *p* = 0.273), with one lacking genotyping data. Thus, it does not appear that dropouts were overrepresented in any of the *CYP2C19* or *CYP2D6* metabolizer groups. For the dropouts for whom MADRS scores and serum levels at Week 2 were available, there were no significant differences in symptom improvement or ESC_adj_ serum concentrations between *CYP2C19* or *CYP2D6* metabolizer phenotypes (Fig. S1). Two participants who did not have genotyping data were also excluded. Therefore, 178 participants were included in the study (211 recruited−31 dropouts−2 lacking genotyping data = 178).

For *CYP2D6*, the effect of genotype on enzymatic function was unclear for five participants, and genotyping was consistently unsuccessful for one participant. For *CYP2C19*, there was one participant with a poor-quality sample. Therefore, analyses were conducted on 177 participants for *CYP2C19* and 170 participants for *CYP2D6* after the exclusion of UMs (*N* = 2). The distribution of metabolizer phenotypes of *CYP2C19* and *CYP2D6* did not differ significantly (*χ*^2^(12) = 6.26, *p* = 0.902).

All participants were adherent to treatment during Phase I based on drug serum levels at Week 2. During Phase II, seven participants were suspected of non-adherence determined by a lack of treatment medication detected in serum at both Weeks 10 and 16, therefore they were not included in Phase II analyses. Non-adherence was not associated with *CYP2C19* (*χ*^2^(2) = 0.29, *p* = 0.867) or *CYP2D6* (*χ*^2^(1) = 1.29, *p* = 0.257) metabolizer groups.

For demographic characteristics stratified by metabolizer group for the study sample and the European subset (see Tables 1 and S4), respectively. Also, see Supplementary Results for a description of the study sample. A summary of metabolizer status and genotypic frequencies of *CYP2C19* and *CYP2D6* can be found in Table S3.

**Table 1:** Basic sample demographics and clinical information.

Characteristic	All		CYP2C19			CYP2D6		
	*N* = 178	NM (*N* = 71)	IM + PM (*N* = 56)	RM + UM (*N* = 50)	*p*-value^a^	NM (*N* = 99)	IM + PM (*N* = 71)	*p*-value^b^
Age	35.43 (12.77)	35.04 (12.96)	35.54 (12.75)	35.94 (12.88)	0.888	36.61 (13.18)	34.44 (12.53)	0.268
*Sex*					0.646			0.755
Female	110 (62%)	41 (58%)	35 (62%)	33 (66%)		59 (60%)	44 (62%)	
Male	68 (38%)	30 (42%)	21 (38%)	17 (34%)		40 (40%)	27 (38%)	
*Ancestry*^c^					<0.001 ***			0.564
Non-European [African (4), East Asian (14), Latin American (9), South Asian (5), South East Asian (4), and mixed ancestry (13)]	49 (28%)	14 (20%)	27 (48%)	8 (16%)		24 (24%)	20 (28%)	
European	129 (72%)	57 (80%)	29 (52%)	42 (84%)		75 (76%)	51 (72%)	
*Previous AD treatment for current MDE*					0.104			0.334
None	104 (58%)	35 (49%)	38 (68%)	30 (60%)		54 (55%)	44 (62%)	
1+	74 (42%)	36 (51%)	18 (32%)	20 (40%)		45 (45%)	27 (38%)	
*ESC dose at Week 8*					0.599			>0.999
10 mg	177 (99%)	71 (100%)	55 (98%)	50 (100%)		98 (99%)	71 (100%)	
20 mg	1 (0.6%)	0 (0%)	1 (1.8%)	0 (0%)		1 (1.0%)	0 (0%)	
*ESC dose at Week 16*					0.633			0.023 *
10 mg	16 (9.8%)	5 (7.5%)	6 (12%)	5 (11%)		5 (5.4%)	11 (17%)	
15 mg	2 (1.2%)	2 (3.0%)	0 (0%)	0 (0%)		2 (2.2%)	0 (0%)	
20 mg	145 (89%)	60 (90%)	43 (88%)	41 (89%)		86 (92%)	54 (83%)	
*Phase II treatment arm*					0.959			0.308
ESC	81 (46%)	33 (46%)	25 (45%)	22 (44%)		41 (41%)	35 (49%)	
ESC + ARI	97 (54%)	38 (54%)	31 (55%)	28 (56%)		58 (59%)	36 (51%)	
Baseline MADRS Score	29.98 (5.50)	30.06 (5.26)	29.75 (5.36)	30.10 (6.11)	0.953	30.60 (5.82)	29.14 (5.09)	0.122
*CYP2C19 Metabolizer Groups*					–			0.510
NM	71 (40%)	–	–	–		42 (42%)	26 (37%)	
IM + PM	56 (32%)	–	–	–		28 (28%)	26 (37%)	
RM + UM	50 (28%)	–	–	–		29 (29%)	19 (27%)	
*CYP2D6 Metabolizer Groups*					0.510			–
NM	99 (58%)	42 (62%)	28 (52%)	29 (60%)		–	–	
IM + PM	71 (42%)	26 (38%)	26 (48%)	19 (40%)		–	–	

Mean (with standard deviation) and frequency are displayed for continuous and categorical variables, respectively.

*AD* Antidepressant, *ARI* aripiprazole, *ESC* escitalopram, *IM* intermediate metabolizer, *MADRS* Montgomery–Åsberg depression rating scale, *MDE* major depressive episode, *NM* normal metabolizer, *PM* poor metabolizer, *RM* rapid metabolizer, *SD* standard deviation, *UM* ultra-rapid metabolizer.

*p < 0.05; **p < 0.01; ***p < 0.001. Statistically significant differences between metabolizer groups are indicated in bold.

^a^Kruskal–Wallis rank sum test; Pearson’s Chi-squared test; Fisher’s exact test.

^b^Wilcoxon rank sum test; Pearson’s Chi-squared test; Fisher’s exact test.

^c^Categories for ancestry are adapted from the International Genome Sample Resource 1000 Genomes Project (http://www.internationalgenome.org/category/population/).

### Association of CYP2C19 and CYP2D6 metabolizer groups with antidepressant response

The overall response rates at the end of Phases I and II were 46.62% (83/178) and 68.54% (122/178), respectively. There was no significant impact of either *CYP2C19* or *CYP2D6* metabolizer groups on response or remission status at the end of Phase I or II (Table S5).

**Fig. 2:**
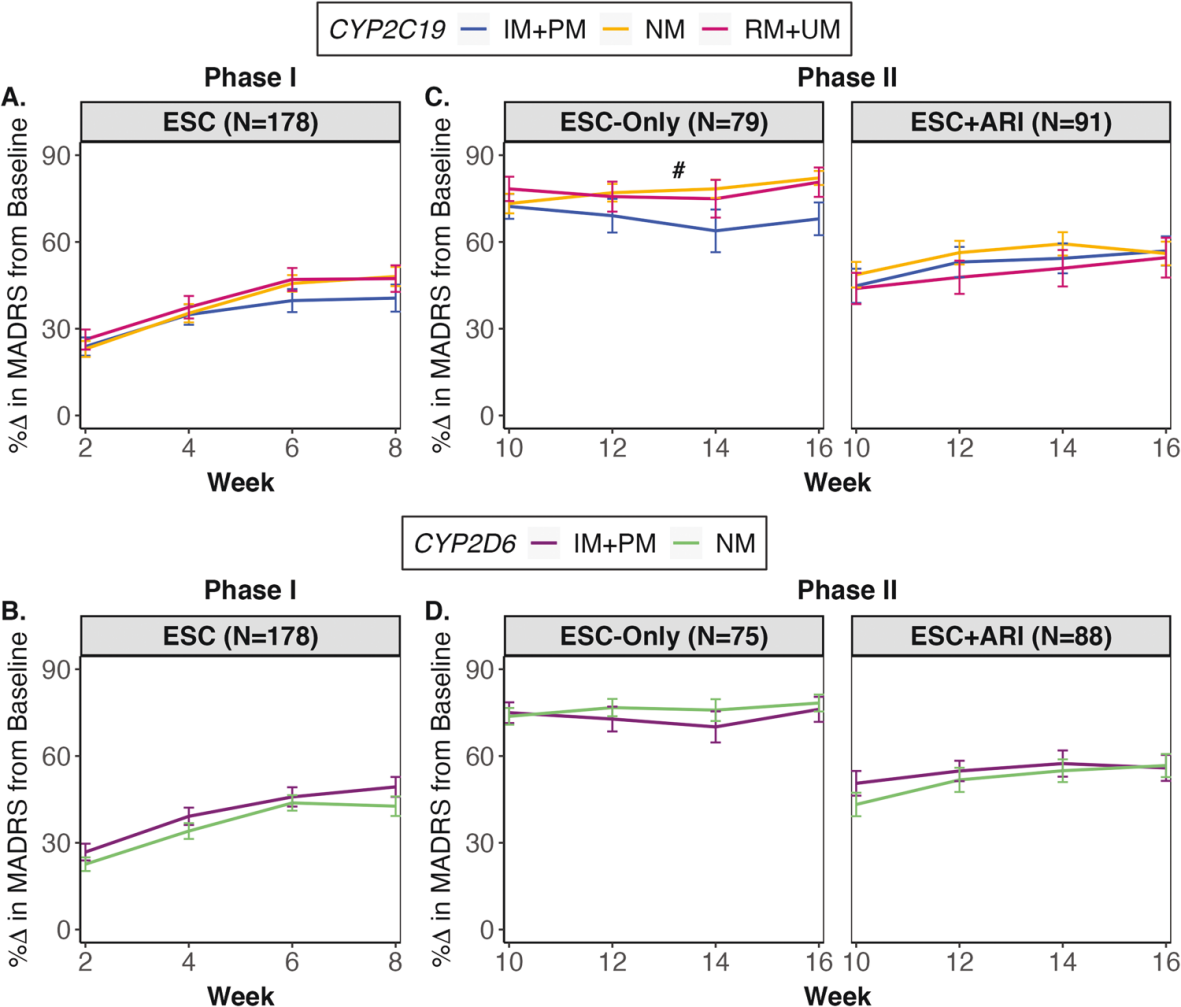
Symptom improvement over time for Phase I and II by *CYP2C19* and *CYP2D6* metabolizer group. During Phase I, **A** *CYP2C19* or **B** *CYP2D6* metabolizer groups did not have a significant influence on symptom improvement over time. During Phase II, for the ESC-Only treatment arm, the average symptom improvement from baseline for every two-week assessment trended towards being lower in **C** *CYP2C19* IM + PMs compared to NMs, which was not observed in the ESC + ARI group. There were no associations between symptom improvement over the course of Phase II and **D** *CYP2D6* metabolizer groups for any of the treatment arms. All linear mixed effects analyses were adjusted for age, ancestry, sex, and interaction between time and *CYP2C19* and *CYP2D6* metabolizer groups as fixed effects, and recruitment site and subject as random effects variables. Error bars represent standard error. ARI aripiprazole, ESC escitalopram, IM intermediate metabolizer, NM normal metabolizer, PM poor metabolizer, RM rapid metabolizer, UM ultra-rapid Metabolizer. # indicates trend with *q* between 0.050 and 0.070.

During Phase I (Weeks 0–8), symptom improvement across timepoints was not significantly influenced by *CYP2C19* or *CYP2D6* metabolizer groups (Fig. 2A, B). During Phase II (Weeks 8–16), in the ESC-Only treatment arm (*N* = 283 observations), there was a trend for an influence of *CYP2C19* metabolizer group on symptom improvement across timepoints (*F*_(2,207)_ = 3.99, *p* = 0.020, *q* = 0.068, *f*^2^ = 0.05). The linear mixed-effects model indicated that the average percentage change in MADRS from baseline was lower in IM + PMs versus NMs (*B* = −2.34, 95% CI: [−3.62, −0.37]) (Fig. 2C, D). Simple effects analysis revealed that the average percentage change in MADRS from baseline was 2.54% (95% CI: [−4.33, −0.74], *p* = 0.006) lower in IMs than in NMs with every two-week assessment, while PMs were not statistically different from NMs (Fig. S2C). Overall, the cumulative difference in symptom improvement from baseline between *CYP2C19* NMs and IMs was 11.52% (*W* = 381, *p* = 0.050, *r* = 0.28) at trial end in ESC-Only. Follow-up mediation analyses revealed that about 45% of the effect of *CYP2C19* IM+PM phenotype on symptom improvement during Phase II in ESC-Only may be mediated by ESC_adj_ serum concentrations (see Supplementary Results).

For ESC + ARI (*N* = 325 observations), the percentage of symptom improvement was uninfluenced by either *CYP2C19* or *CYP2D6* metabolizer groups (Table S7). Associations between symptom improvement and *CYP2C19* and *CYP2D6* metabolizer groups were not observed in the European subset following multiple testing corrections (Table S8).

### Associations of CYP2C19 and CYP2D6 metabolizer groups with antidepressant side effects

Analyses of the associations of *CYP2C19* and *CYP2D6* metabolizer groups with the absence or presence of CNS, GI, sexual side effects and treatment-related weight gain are presented in Table S9. Differences in sample sizes between weeks or side effect categories are accounted for by missed visits or failure to respond to the corresponding item on the TSES.

**Fig. 3:**
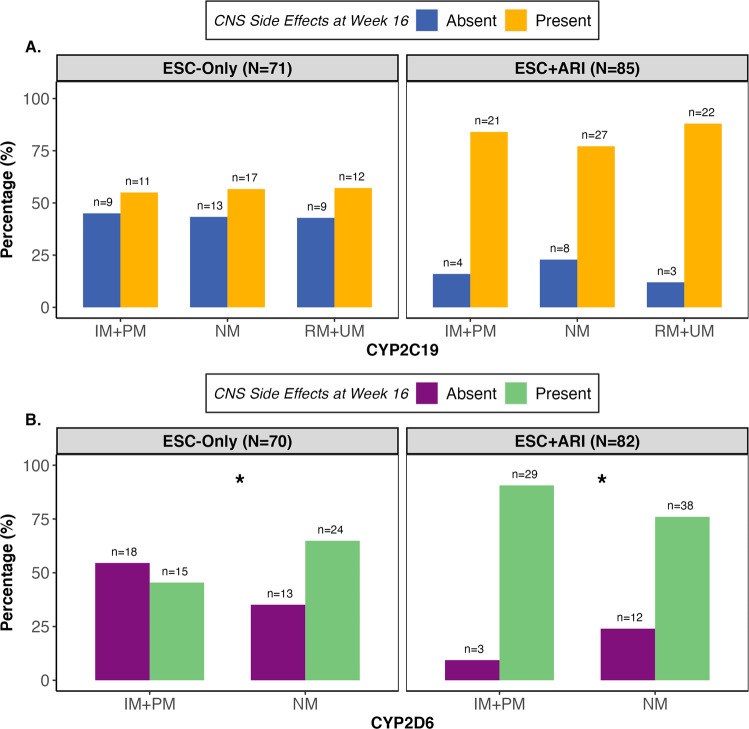
Central nervous system (CNS) side effects self-reported to be present or absent by *CYP2C19* and *CYP2D6* metabolizer groups and treatment arm. **A** Central Nervous System (CNS) side effects did not show an association with *CYP2C19* metabolizer group. **B** Presence of CNS side effects was influenced by *CYP2D6* metabolizer group. In ESC-Only, the odds of reporting a CNS side effect was 7.69 (SE = 6.09, 95% CI 1.63, 36.30) times higher for NMs compared to IM + PMs (*χ*^2^ (1, *N* = 70) = 6.65, *p* = 0.010, *q* = 0.048). The ESC + ARI treatment arm also showed an association between CNS side effects and *CYP2D6* metabolizer group (*χ*^2^ (1, *N* = 82) = 6.70, *p* = 0.010, *q* = 0.049). The odds of reporting a CNS side effect 11.52 (SE = 10.90, 95% CI 1.80, 73.35) times higher for IM + PMs compared to NMs in this treatment arm. All logistic regression analyses were adjusted for age, ancestry, sex, recruitment site, total MADRS score at baseline, *CYP2C19* and *CYP2D6* metabolizer groups. *P*-values are corrected for multiple testing using the false discovery rate (FDR) approach. ARI aripiprazole, ESC escitalopram, IM intermediate metabolizer, NM normal metabolizer, PM poor metabolizer, RM rapid metabolizer, UM ultra-rapid metabolizer. *q < 0.05.

The presence of CNS side effects was associated with *CYP2D6* metabolizer group in ESC-Only (*χ*^2^(1, *N* = 70)=6.65, *p* = 0.009, *q* = 0.048) and ESC + ARI (*χ*^2^(1, N = 82)=6.70, *p* = 0.006, *q* = 0.049) at Week 16 (Fig. 3B). For ESC-Only, the odds of reporting a CNS side effect were higher by a factor of 7.69 (95% CI [1.63, 36.30]) for NMs compared to IM + PMs. Within the category of CNS side effects, NMs had 25.53 (95% CI [1.99, 328.18]) and 29.20 (95% CI [1.26, 676.37]) higher odds of reporting decreased sleepiness and sweating, respectively, compared to IM + PMs (Fig. S3A, B). In contrast, for ESC + ARI, the odds of reporting a CNS side effect were 11.52 (95% CI [1.81, 73.35]) times higher for IM + PMs compared to NMs. Specifically for this treatment arm, IM + PMs were likelier to report postural hypotension compared to NMs (OR = 8.07, 95% CI [1.51, 43.04]) (Fig. S3C). Post-hoc comparisons did not show a significant association between the presence of CNS side effects at Week 16 and ungrouped *CYP2D6* metabolizer phenotypes in either treatment arm (Fig. S4).

Presence of sexual side effects was significantly associated with *CYP2D6* metabolizer group in only ESC + ARI at Week 16 (*χ*^2^(1, *N* = 81) = 8.26, *p* = 0.004, *q* = 0.046) (Fig. S5B). The odds of reporting a sexual side effect were 6.72 (95% CI [1.83, 24.67]) times higher for IM + PMs compared to NMs. Specifically, *CYP2D6* IM + PMs had an increased likelihood of reporting decreased libido compared to NMs (OR = 9.63, 95% CI [1.97, 47.04]) (Fig. S6). Post-hoc tests revealed IMs (*N* = 28) had 3.27 (95% CI [1.17, 9.18]) higher odds of reporting the presence of sexual side effects compared to NMs (*N* = 54), specifically decreased libido (OR = 3.85, 95% CI: [1.30, 11.39]), while the likelihood of reporting a sexual side effect was not significantly different between NMs and PMs (*N* = 6) (Fig. S7).

No associations between the intensity of side effects across timepoints with either *CYP2C19* or *CYP2D6* metabolizer groups were observed (Figs. S10–13). Further, when these tests were repeated in the European subset, no associations were observed (Tables S13 and S14).

### Association of CYP2C19 and CYP2D6 metabolizer groups with ESC and ARI exposure

#### Serum ESC concentrations

**Fig. 4:**
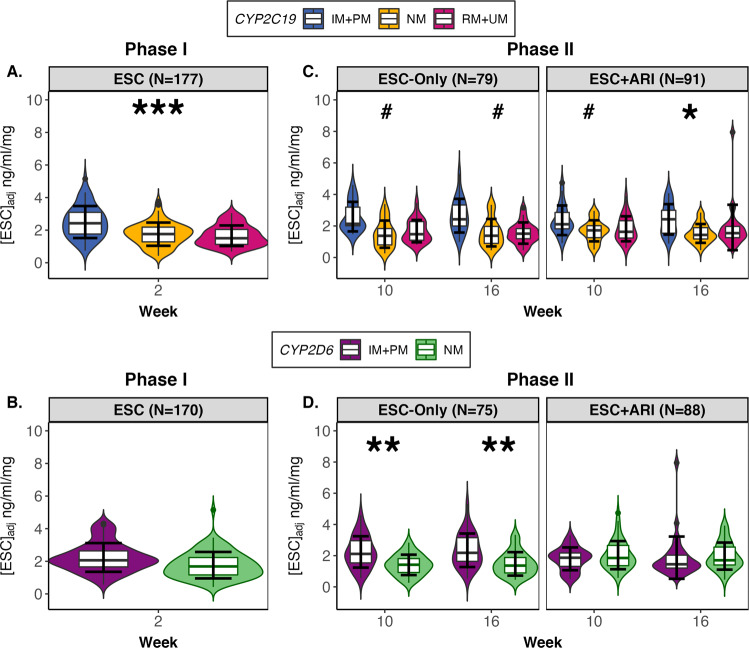
Dose-adjusted ESC concentrations in serum for Phase I and II by *CYP2C19* and *CYP2D6* metabolizer groups. During Phase I, **A** *CYP2C19* IM + PMs showed higher mean ESC_adj_ concentrations relative to NMs, whereas there was no significant difference in ESC_adj_ concentrations between NMs and RM + UMs. **B** A significant difference in ESC_adj_ concentrations was not observed between *CYP2D6* metabolizer group. During Phase II, for the ESC-Only treatment arm, **C** *CYP2C19* and **D** *CYP2D6* IM + PMs compared to NMs had higher ESC levels in serum. In the ESC + ARI treatment arm, ESC_adj_ serum levels were associated with only **C** *CYP2C19*, but not **D** *CYP2D6*, with higher ESC_adj_ concentrations in *CYP2C19* IM + PMs relative to NMs. All linear regression analyses were adjusted for age, ancestry, sex, recruitment site, time since last dose, *CYP2C19* and *CYP2D6* metabolizer groups. Error bars represent standard error. ARI aripiprazole, ESC escitalopram, IM intermediate metabolizer, NM normal metabolizer, PM poor metabolizer, RM rapid metabolizer, UM ultra-rapid metabolizer. *q < 0.05; **q < 0.01; ***q < 0.001; # indicates trend with *q* between 0.050 and 0.070.

For Phase I, serum levels of ESC were available for 175 participants. Using linear regression analyses, we observed a significant association between serum ESC_adj_ concentrations at Week 2 and *CYP2C19* metabolizer group (*F*_(2,147)_ = 12.54, *p* < 0.001, *q* < 0.001, *f*^2^ = 0.13) (Table S15). *CYP2C19* IM + PMs showed 42.9% higher mean ESC_adj_ concentrations compared to NMs (*B* = 0.59, 95% CI: [0.30, 0.87]), while NMs and RM + UMs were not significantly different (Fig. 4A). Simple effects tests showed IMs (*N* = 51) and PMs (*N* = 5) when ungrouped also had higher ESC levels compared to NMs (Fig. S14A). In the European subset, the same association between Week 2 ESC_adj_ concentrations and *CYP2C19* metabolizer groups was observed (*F*_(2,110)_ = 6.45, *p* = 0.002, *q* = 0.028, *f*^2^ = 0.10) (Table S16). *CYP2D6* metabolizer groups did not show a significant association with ESC_adj_ levels at Week 2 (Fig. 4B).

For Phase II, serum levels of ESC_adj_ were available for 153 and 152 participants at Weeks 10 and 16, respectively. In ESC-Only, there was a trend for an association between *CYP2C19* metabolizer group and ESC_adj_ concentrations for Weeks 10 (*F*_(2,53)_ = 4.98*, p* = 0.013, *q* = 0.056, *f*^2^ = 0.11) and 16 (*F*_(2,54)_ = 5.26*, p* = 0.016, *q* = 0.062, *f*^2^ = 0.13) (Table S15). Relative to NMs, *CYP2C19* IM + PMs demonstrated higher levels of ESC_adj_ in serum by 75.0% at Week 10 (*B* = 0.65, 95% CI: [0.14, 1.17]), and by 68.8% at Week 16 (*B* = 0.66, 95% CI: [0.13, 1.19]), while RM + UMs were not significantly different (Fig. 4C). Likewise, there was a significant association between ESC_adj_ concentrations and *CYP2D6* metabolizer group at Weeks 10 (*F*_(2,53)_ = 7.65*, p* < 0.001, *q* = 0.004, *f*^2^ = 0.11) and 16 (*F*_(2,54)_ = 8.45*, p* < 0.001, *q* = 0.004, *f*^2^ = 0.13). Specifically, *CYP2D6* IM + PMs demonstrated higher levels of ESC_adj_ in serum by 58.9% (*B* = 0.65, 95% CI: [0.14, 1.17]) at Week 10 and by 59.2% (*B* = 75, 95% CI: [0.35, 1.15]) at Week 16, compared to NMs (Fig. 4D). IMs (*N* = 30) and PMs (*N* = 5) ungrouped showed the same effect, with PMs demonstrating the highest serum levels relative to NMs (Fig. S14C). The European subset showed a significant association between ESC_adj_ levels and *CYP2D6* metabolizer groups at Weeks 10 (*F*_(1,40)_ = 6.25, *p* = 0.001, *q* = 0.011, *f*^2^ = 0.09) and 16 (*F*_(1,38)_ = 10.37, *p* < 0.001, *q* < 0.001, *f*^2^ = 0.10), but not with *CYP2C19* (Table S16).

In ESC + ARI, we observed that there was an association between *CYP2C19* metabolizer group and ESC_adj_ concentrations at Weeks 10 (*F*_(2,64)_ = 6.27*, p* = 0.009, *q* = 0.049, *f*^2^ = 0.15) and 16 (*F*_(2,61)_ = 6.58*, p* = 0.002, *q* = 0.040, *f*^2^ = 0.20). The strength of the association was stronger over time, with the difference between *CYP2C19* NMs and IM + PMs increasing from 39.6% (*B* = 0.69, 95% CI: [0.24, 1.13]) at Week 10 to 58.2% (*B* = 0.77, 95% CI: [0.37, 1.17]) at Week 16 (Fig. 4C). Post-hoc comparisons show ungrouped IMs (*N* = 27) and PM (*N* = 1) have higher ESC_adj_ concentrations relative to NMs at Weeks 10 and 16 (Fig. S14C). Of note, there was no association between ESC_adj_ serum concentrations and *CYP2D6* metabolizer group for ESC + ARI (Fig. 4D). *CYP2C19* and *CYP2D6* metabolizer groups were not significantly associated with ESC_adj_ concentrations in the European subset (Table S16).

Interestingly, between the two treatment arms, *CYP2D6* NMs in ESC + ARI demonstrate significantly higher ESC_adj_ serum levels compared to *CYP2D6* NMs in ESC-Only at both Weeks 10 (*W* = 381, *p* = 0.001, *r* = −0.52) and 16 (*W* = 549, *p* = 0.006, *r* = −0.47), whereas *CYP2C19* NMs do not differ in ESC_adj_ concentrations by treatment arm (Fig. S15).

#### Serum S-DCT concentrations

At Weeks 2, 10, and 16, serum levels of S-DCT were available for 172, 152, and 150 participants, respectively. There was no significant association between S-DCT_adj_ serum concentrations and *CYP2C19* or *CYP2D6* metabolizer groups (Fig. S16).

#### Serum S-DCT/ESC ratio

**Fig. 5:**
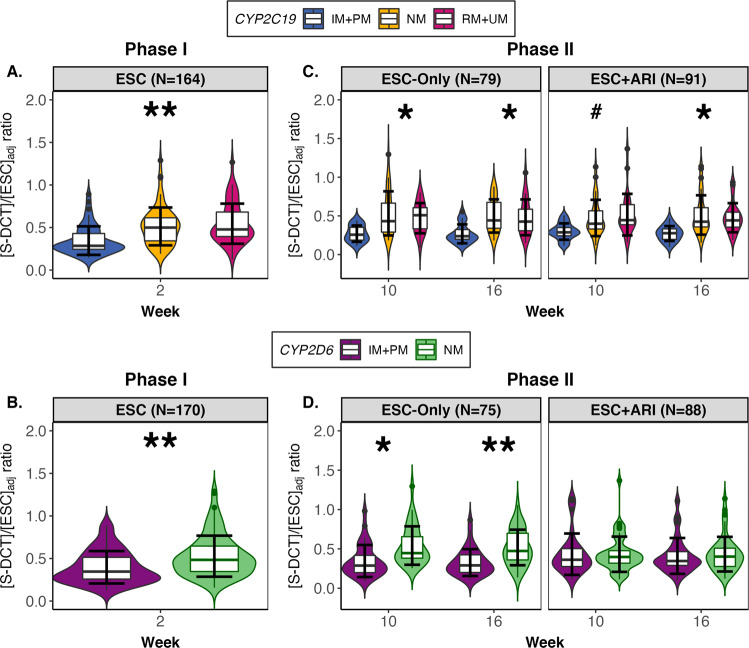
Dose-adjusted serum S-DCT/ESC_adj_ ratio for Phase I and II by *CYP2C19* and *CYP2D6* metabolizer groups. During Phase I, **A** *CYP2C19* and **B** *CYP2D6* IM + PMs showed lower mean S-DCT_ad_/ESC_adj_ ratio relative to NMs. Likewise, during Phase II, in the ESC-Only and ESC + ARI treatment arms, **C** *CYP2C19* IM + PMs compared to NMs had lower mean S-DCT_ad_/ESC_adj_ ratio in serum. **D** For *CYP2D6*, IM + PMs displayed lower S-DCT_ad_/ESC_adj_ ratio in ESC-Only, whereas S-DCT_ad_/ESC_adj_ ratio was not associated with *CYP2D6* metabolizer group in the ESC + ARI treatment arm. All linear regression analyses were adjusted for age, ancestry, sex, site, time since last dose, *CYP2C19* and *CYP2D6* metabolizer groups. Error bars represent standard error. ARI aripiprazole, ESC escitalopram, IM intermediate metabolizer, NM normal metabolizer, PM poor metabolizer, RM rapid metabolizer, UM ultra-rapid metabolizer. *q < 0.05; **q < 0.01; ***q < 0.001.

There were significant effects of *CYP2C19* and *CYP2D6* metabolizer status on S-DCT_adj_/ESC_adj_ serum ratio at Week 2 (*CYP2C19*: *F*_(2,144)_ = 0.66*, p* < 0.001, *q* = 0.003, *f*^2^ = 0.10; *CYP2D6*: *F*_(1,144)_ = 0.44*, p* < 0.001, *q* = 0.007, *f*^2^ = 0.10) (Table S15). With NMs as the reference group, *CYP2C19* IM + PMs demonstrated lower mean S-DCT_adj_/ESC_adj_ ratio by 32.6% (*B* = −0.13, 95% CI: [−0.20, −0.05]), whereas RM + UMs were not significantly different (Fig. 5A). Likewise, IM + PMs of *CYP2D6* had S-DCT_adj_/ESC_adj_ ratio that is 24.7% (*B* = −0.11, 95% CI: [−0.19, −0.02]) lower than NMs (Fig. 5B). *CYP2C19* and *CYP2D6* PMs (*N* = 5 and 2, respectively) showed the lowest DCT_adj_/ESC_adj_ ratio relative to the other metabolizer phenotypes when ungrouped (Fig. S17A, B). The European subset showed the same association between DCT_adj_/ESC_adj_ and *CYP2C19* (*F*_(2,108)_ = 0.47, *p* = 0.002, *q* = 0.028, *f*^2^ = 0.09) and *CYP2D6* (*F*_(1,108)_ = 0.45, *p* < 0.001, *q* = 0.027, *f*^2^ = 0.10) metabolizer groups at Week 2 (Table S16).

For Phase II, in ESC-Only, S-DCT_adj_/ESC_adj_ ratio showed an association with *CYP2C19* and *CYP2D6* metabolizer groups at Weeks 10 (*CYP2C19*: *F*_(2,52)_ = 0.41*, p* = 0.009, *q* = 0.048, *f*^2^ = 0.13; *CYP2D6*: *F*_(1,52)_ = 0.44*, p* = 0.002, *q* = 0.015, *f*^2^ = 0.16) and 16 (*CYP2C19*: *F*_(2,53)_ = 0.29*, p* = 0.009, *q* = 0.048, *f*^2^ = 0.16; *CYP2D6*: *F*_(1,53)_ = 0.55*, p* < 0.001, *q* = 0.001, *f*^2^ = 0.27) (Fig. 5C, D). *CYP2C19* IM + PMs demonstrated S-DCT_adj_/ESC_adj_ ratios lower by 49.3% (*B* = −0.19, 95% CI: [−0.34, −0.05]) and 46.3% (*B* = −0.16, 95% CI: [−0.28, −0.05]) at Weeks 10 and 16 relative to NMs, respectively (Table S15). For *CYP2C19*, IM + PMs showed lower ratios by 36.2% (*B* = −0.17, 95% CI: [−0.28, −0.08]) and by 36.9% (*B* = −0.20, 95% CI: [−0.28, −0.11]) at Weeks 10 and 16, respectively. Interestingly, in the European subset, S-DCT_adj_/ESC_adj_ ratio was associated with *CYP2D6* at Weeks 10 (*F*_(1,39)_ = 0.41*, p* = 0.005, *q* = 0.043, *f*^2^ = 0.18) and 16 (*F*_(1,37)_ = 0.60*, p* < 0.001, *q* = 0.003, *f*^2^ = 32), but with *CYP2C19* metabolizer group (Table S16).

Similarly, in ESC + ARI during Phase II, S-DCT_adj_/ESC_adj_ ratio was influenced by *CYP2C19* metabolizer group at Weeks 10 (*F*_(2,63)_ = 0.51*, p* = 0.006, *q* = 0.046, *f*^2^ = 0.16) and 16 (*F*_(2,59)_ = 0.59*, p* = 0.001, *q* = 0.040, *f*^2^ = 0.20), but not by *CYP2D6* (Fig. 5C, D). IM + PMs have S-DCT_adj_/ESC_adj_ ratio that is 37.0% (*B* = −0.17, 95% CI: [−0.29, −0.04]) and 46.9% (*B* = −0.22, 95% CI: [−0.34, −0.10]) lower than NMs at Weeks 10 and 16, respectively (Table S15). This association is observed in the European subset only at Week 16 (*F*_(2,43)_ = 0.56*, p* = 0.001, *q* = 0.040, *f*^2^ = 0.25) (Table S16).

#### Serum levels of ARI, DHA, and DHA/ARI ratio

A trend for an association between *CYP2D6* metabolizer group and DHA at Week 10 (*F*_(1,61)_ = 13.07, *p* = 0.006, *q* = 0.046, *f*^2^ = 0.12) and ARI_adj_/DHA_adj_ ratio at Week 16 (*F*_(1,61)_ = 0.07, *p* = 0.010, *q* = 0.049, *f*^2^ = 0.12) was observed (Fig. S18B, C). IM + PMs showed 17.4% lower DHA (*B* = −0.99, 95% CI: [−1.67, −0.30]) and 19.8% lower ARI_adj_/DHA_adj_ ratio (*B* = −0.04, 95% CI: [−0.09, −0.01]) relative to NMs (Table S17). Simple effects tests revealed that PMs (*N* = 6) had significant lower DHA and ARI_adj_/DHA_adj_ ratio relative to the other metabolizer phenotypes when ungrouped (Fig. S19). Serum levels of ARI_adj_, DHA_adj_, and the DHA_adj_/ARI_adj_ ratio were not significantly associated with *CYP2D6* metabolizer group in the European subset (Table S18).

### Association of ESC and ARI exposure with antidepressant response and side effects

Results of the Spearman correlation indicated that serum levels of ESC, S-DCT, and their ratio were not associated with symptom improvement, CNS or gastrointestinal side effects, or treatment-related weight gain during Phase I and II. In ESC-Only, there was a significant negative association between the intensity of sexual side effects and serum ESC concentrations at Week 10 (*r*_s_(67) = −0.36, *p* = 0.002, *q* = 0.035), while ESC + ARI did not show this effect (Table S19). The lower the serum levels of ESC levels, the greater the intensity of sexual side effects that are reported. Post-hoc analyses revealed this effect was driven by a significant correlation between concentrations of ESC and intensity of anorgasmia (*r*_s_(67) = −0.32, *p* = 0.007) and decreased libido (*r*_s_(67) = −0.25, *p* = 0.038) (Fig. S20). Serum ARI and DHA levels were correlated with symptom improvement for the ESC + ARI treatment arm (*r*_s_(83) = −0.36, *p* = 0.001, *q* = 0.048; *r*_s_(83) = −0.35, *p* = 0.002, *q* = 0.048, respectively). The higher the concentrations of ARI and DHA, the lower the percent change in MADRS from baseline at Week 16 (Fig. S21).

## Discussion

To the best of our knowledge, the current study is the first to assess the relationship of *CYP2C19* and *CYP2D6* metabolizer groups with the response, tolerability, and serum concentrations in individuals on ARI augmentation of ESC for the treatment of MDD, which represents a commonly used practice.

Our results showed that *CYP2C19* IM + PMs demonstrated a trend towards lower symptom improvement (i.e., the percentage change in MADRS from baseline) during Phase II than NMs amongst patients on ESC monotherapy. Post-hoc comparisons revealed that there was a significant difference specifically between *CYP2C19* NMs and IMs, demonstrating a cumulative difference of 11.5% in percentage MADRS change by Week 16, whereas NMs and PMs did not differ, likely due to the small number of PMs in the sample. However, when the clinical relevance of this effect was evaluated using response and remission status, we found that the proportion of responders and non-responders, as well as remitters and non-remitters, did not differ by *CYP2C19* metabolizer group. Therefore, although we observed that symptom improvement trended towards being different between *CYP2C19* IM + PMs versus NMs in patients on ESC monotherapy, the size of this effect was small (*f*^2^ = 0.05) and of limited clinical utility. Therefore, our findings warrant further validation in a larger, independent sample.

Further, we showed that ESC serum concentrations are influenced by *CYP2C19* metabolizer group. *CYP2C19* IM + PMs demonstrated a trend towards higher ESC_adj_ serum concentrations and lower S-DCT_adj_/ESC_adj_ ratio in comparison to NMs during Phase I and in both treatment arms during Phase II, which replicates findings from previous studies [14, 15, 19, 37]. The mediation analysis showed that about 45% of the effect of *CYP2C19* IM + PM phenotype on symptom improvement may be mediated by ESC_adj_ serum levels in ESC-Only. These findings taken together suggest that for slower *CYP2C19* metabolizers, there are higher concentrations of ESC in serum possibly above the therapeutic range, which negatively impacts symptom improvement over time in individuals on ESC monotherapy (Fig. S23). Therefore, individuals who are *CYP2C19* IM and PM may benefit from ESC dose reductions to achieve greater improvements in depressive symptomology.

The association between *CYP2D6* metabolizer group and serum measures of study medications is more complex due to ESC and its metabolite, S-DCT, being weak inhibitors of CYP2D6 in vitro [8, 38]. The underlying mechanisms and the extent to which CYP2D6 activity is affected by ESC inhibition remain to be elucidated. It has been reported that IMs may be more susceptible to phenoconversion by concomitant use of weak to moderate CYP2D6 inhibitors compared to PMs, NMs, and UMs [39]. In this study, *CYP2D6* IMs demonstrated elevated ESC concentrations similar to concentrations in PMs, suggesting possible phenoconversion of CYP2D6 IMs by ESC into a lower metabolizer phenotype (Fig. S14). In contrast, ESC is not a known inhibitor of CYP2C19, thus *CYP2C19* NMs, IMs, and PMs showed differences in ESC_adj_ concentrations consistent with their predicted enzymatic capacity.

Although both treatment arms are affected by possible phenoconversion of CYP2D6 by ESC and its metabolite, a significant association between *CYP2D6* metabolizer group and ESC_adj_ serum concentrations was observed in ESC-Only, but not in ESC + ARI (Fig. 4D). This suggests that there may be a difference in the pharmacokinetics of ESC between the two treatment arms, with ESC + ARI being affected by ARI coadministration. We postulated that the differential effect of *CYP2D6* metabolizer group on ESC_adj_ serum levels between treatment arms is due to the competition for CYP2D6 by both ESC (a CYP2D6 weak inhibitor) and ARI (a CYP2D6 substrate) in the ESC + ARI treatment arm. This postulation is supported by the observation that, during Phase II, *CYP2D6* NMs in ESC + ARI consistently demonstrated higher ESC_adj_ serum levels compared to *CYP2D6* NMs in ESC-Only. This is possibly due to more unmetabolized ESC in serum in ESC + ARI as a result of competition with ARI for CYP2D6, which is not present in ESC-Only (Fig. S15). This competition for CYP2D6 by both ESC and ARI shifts the metabolism of ESC to be more dependent on CYP2C19, hence resulting in a lack of a significant association between ESC_adj_ concentrations and *CYP2D6* metabolizer group in ESC + ARI. In ESC-Only, where there is no competition for CYP2D6 by ARI, the metabolism of ESC is dependent on both CYP2C19 and CYP2D6, resulting in a significant association of ESC_adj_ concentrations with both enzymes.

For treatment-related side effects, *CYP2D6* NMs and IM + PMs had a higher likelihood of reporting a CNS side effect in ESC-Only and ESC + ARI, respectively. The mechanism underlying this difference between treatment arms in the direction of association between *CYP2D6* metabolizer group and the presence of CNS side effects is unclear. CYP2D6 is reported to be expressed centrally, where it may be enzymatically active and involved in the metabolism of endogenous compounds, including neuronal amines (e.g. tyramine to dopamine), as well as peripherally administered drugs [40, 41]. Like hepatic CYP2D6, brain CYP2D6 can also be induced and inhibited by medications; therefore, it is possible that the direction of the effect was different between treatment arms due to the phenoconversion of brain CYP2D6 by ESC and competition for CYP2D6 by both ESC and ARI in ESC + ARI, affecting the metabolism and functioning of endogenous systems, including serotonin and dopamine [42].

Our analyses included both *CYP2C19* and *CYP2D6* metabolizer groups as fixed effects in the same model. This approach was previously shown to be a better predictor of ESC blood levels than when *CYP2C19* and *CYP2D6* were evaluated individually [43]. A further strength of our study included the repeated measures approach of assessing symptom improvement and intensity of side effects over time which increased statistical power. The limitations of this study include the relatively small sample size when stratifying by treatment arm during Phase II, which limits statistical power to detect differences in response and side effects by CYP450 metabolizer groups. However, our sample was adequately powered to investigate differences in serum levels by CYP450 metabolizer groups during both Phases. Further, we were limited by the low representation of the less common PMs and UMs; therefore, because of power considerations, we employed grouping together slower and faster metabolizer phenotypes. As a result, we conducted post-hoc comparisons for significant results using ungrouped metabolizer phenotypes to distinguish which specific pairs of phenotypes are different. Another study limitation is that comedication was not recorded which might have modulated CYP2C19 or CYP2D6 activity and resulted in phenoconversion [39, 44]. Similarly, one subject reported a liver condition and three subjects reported alcohol misuse, which might have affected medication metabolism. Finally, heterogeneity in ancestry within the study sample raises the issue of population stratification resulting in false positive associations. To ameliorate this issue, we conducted the same analyses in only Europeans and identified discrepancies in the results between the total sample and the European subsample which need to be explored in future studies with a larger sample.

In summary, our results shed some light on the pharmacokinetics of ESC in vivo. We provided support for the phenoconversion of CYP2D6 by ESC and S-DCT inhibition, which was previously shown in vitro. In ESC + ARI, our results revealed altered pharmacokinetics of ESC with ARI coadministration, both of which may be competing for CYP2D6. Of particular interest is our finding showing that *CYP2C19* IM + PMs demonstrated higher ESC_adj_ concentrations and trended towards lower symptom improvement relative to NMs amongst those on ESC monotherapy. These results suggest preemptive *CYP2C19* genotyping may be useful to identify patients who are *CYP2C19* IM or PM, so that they may be treated with a reduced dose to optimize their treatment outcome. These results are in line with the recommendations provided by CPIC, confirming that “a 50% reduction of recommended starting dose” should be considered for *CYP2C19* PMs. Based on these results, dose reductions may also be considered for *CYP2C19* IMs. Further, we found an association between *CYP2D6* metabolizer group with CNS and sexual side effects, differentially by treatment arm, indicating that *CYP2D6* genotyping may also preemptively identify patients susceptible to these side effects.

## Supplementary information

Supplementary Materials
