# Supplementary material for: Effects of CYP2C19 and CYP2D6 gene variants on escitalopram and aripiprazole treatment outcome and serum levels: results from the CAN-BIND 1 study
Source: Transl Psychiatry. 2022 Sep 6;12:366. doi: 10.1038/s41398-022-02124-4 (PMC9448818; doi:10.1038/s41398-022-02124-4)
Supplement: Supplementary file 1 — Supplementary Materials [file 41398_2022_2124_MOESM1_ESM.pdf]

SUPPLEMENTARY MATERIALS

Supplementary Methods

Clinical Sample

Participants included in this study were recruited from six different clinical centers across Canada including: Djavad Mowafaghian Centre for Brain Health in Vancouver, BC (n = 63), Hotchkiss Brain Institute in Calgary, AB (n = 33), Providence Care Hospital in Kingston, ON (n = 21), St. Joseph's Healthcare Hamilton in Hamilton, ON (n = 32), and University Health Network (n = 53) and Centre for Addiction and Mental Health (n = 9) in Toronto , ON (total sample, n = 211). Written, informed consent was obtained from all participants. All procedures of this study comply with the ethical standards for research with humans of each participating institution.

Collection of blood samples and quantification of serum drug and metabolite concentrations

Participants self-reported the time at which the last dosage of the study medication was taken during the collection of the blood samples. Serum concentration measurements of ESC, ARI and their respective major metabolites, (S-DCT) and (DHA), were performed at the CAMH Clinical Laboratory using LC-MS/MS technology. Briefly, a mix of deuterated internal standards (Cerilliant) was added to 100 μL of each serum specimen, calibrator (Cerilliant) and quality control (MassCheck Antidepressants/Neuroleptics, Level 1 and Level 2). Proteins were precipitated with 300 μL of 9:1 Acetonitrile:Methanol (Sigma-Aldrich) followed by 5 min centrifugation at 9000 rpm. 25 μL of each supernatant was diluted with 200 μL of 0.1% Formic acid (Sigma-Aldrich) and then analyzed on the LC-MS/MS platform consisting of ThermoFisher TSQ Quantum Ultra mass spectrometer coupled with ThermoFisher Surveyor LC pump and HTC PAL autosampler fitted with a Kinetex F5 2.6 μm, 100 x 2.1 mm column (Phenomenex). Six minute gradient elution with 0.1% Formic Acid (mobile phase A) and Acetonitrile (mobile phase B) was applied. Collision energies ranged from 14 to 20. Mass transitions (M+H) were monitored in SIM mode as follows: ARI (448 →285); DHA (446→285); Citalopram (325 →109); Desmethyl-citalopram (314 →109) Quantification was performed against a 7-point calibration curve ranging from 10 to 1000 ng/mL for each analyte. Assay limit of detection was 5 ng/mL. Vendor nominal means for quality controls were used to assess the assay accuracy for each of the four analytes at Level 1 and Level 2, respectively, and ± 15 % of nominal mean value combined with <15 % analytical precision was considered acceptable assay performance. Both the laboratory means and the assay precisions were within the set acceptance criteria. Data was analyzed with ThemoFisher XCalibur software.

Description of measures of response outcome

Response status signifies the classification of patients as "responder" or "non-responder" if MADRS reduction of ≥50% or <50% from baseline was reached, respectively. For remission status, patients are classified as "remitter" if they achieved a MADRS total score of 10 or less; otherwise, they are classified as "non-remitter". The percentage of symptom improvement was calculated by subtracting the total MADRS score from the baseline MADRS score and then dividing by the baseline MADRS score for each of the eight time points. These measures of response were selected, since continuous measures, such as percentage symptom improvement, capture more information and have higher power, whereas dichotomous measures, such as response status, has a particular clinical relevance since it is associated with MDD prognosis.

Post-hoc mediation analyses

Mediation analyses were performed to investigate whether measures of ESC exposure mediated any significant relationships between *CYP2C19* or *CYP2D6* metabolizer groups and the outcome measures. The strength of the association between the *CYP2C19* or *CYP2D6* metabolizer groups (predictor variable) and measures of treatment response or side effect (outcome variable) must be attenuated or rendered non-significant when controlling for measures of ESC exposure (mediator variable) to establish partial or complete mediation, respectively.[1,2] R package "Mediation"[3] and "lavaan"[4] was used for continuous and binary variables, respectively, to perform nonparametric bootstrap analyses with 5000 iterations to calculate the estimate of the indirect (i.e. mediator) effect, which was considered significant when the 95% confidence interval (CI) excluded zero at $p<0.05$.

References

[1]Baron RM, Kenny DA. The moderator–mediator variable distinction in social psychological research: Conceptual, strategic, and statistical considerations. *Journal of Personality and Social Psychology* 1986; **51**: 1173–1182.

[2]MacKinnon DP, Lockwood CM, Hoffman JM, West SG, Sheets V. A Comparison of Methods to Test Mediation and Other Intervening Variable Effects. *Psychol Methods* 2002; **7**: 83.

[3]Tingley D, Yamamoto T, Hirose K, Keele L, Imai K, Trinh M *et al. mediation: Causal Mediation Analysis*. 2019 https://CRAN.R-project.org/package=mediation (accessed 7 Apr2021).

[4]Savalei V, Rosseel Y. Computational Options for Standard Errors and Test Statistics with Incomplete Normal and Nonnormal Data in SEM. *Structural Equation Modeling: A Multidisciplinary Journal* 2021; : 1–19.

Supplementary Results

Description of Study Sample

There were six cases of protocol deviations during Phase II, in which four responders at Week 8 received ESC + ARI, two non-responders remained on ESC monotherapy, and one responder started receiving ARI augmentation around the end of Phase II. These individuals were included in the treatment group in which they had been inadvertently allocated, except for the latter participant who remained in the ESC-Only group, since they were on ESC monotherapy for the majority of their treatment.

Analyses were conducted on 178 participants following exclusions with a mean age of 35.43 years (SD=12.77, range of 18-61 years) of which 110 (61.8%) of participants were female and 68 (38.2%) were male ($\chi 2(1.\ N=178)=9.91$, $p=0.002$). Although a total of 129 (72.5%) participants were European, while 49 (27.5%) were non-European ($\chi 2(1.\ N=178)=35.96$, $p<0.001$), Europeans were significantly overrepresented within *CYP2C19* NMs and RMs compared to non-European participants ($\chi 2(5.\ N=178)=21.32$, $p<0.001$). 42% of participants were treated with AD at least once previously (range 1-5), whereas 58% were treatment naïve for their current major depressive episode. Eighteen (10%) were not taking concomitant medicines, including herbal and multivitamins.

Almost all participants (99%) were on ESC at 10 mg during Phase I, and then most received an increase in dosage to 20 mg during Phase II (89%). When splitting Phase II by treatment arm, 14% (n=10) and 6% (n=5) received 10 mg in the ESC-Only and ESC+ARI treatment arms, respectively, while one from each treatment arm received 15 mg, and the remaining received 20 mg. ARI dosage also did not vary in the ESC+ARI treatment arm with the majority of participants (98%) receiving 2 mg.

Mediating effects of ESC exposure on significant associations of *CYP2C19* and *CYP2D6* metabolizer groups with outcome measures.

Since a trend was observed for an association between *CYP2C19* metabolizer status and symptom improvement during Phase II in the ESC-Only treatment arm, *post-hoc* mediation analyses were conducted to assess whether measures of ESC exposure were mediating this relationship. As shown in **Figure S22A-B**, there was a significant difference between *CYP2C19* IM+PMs and NMs in symptom improvement (Model 1: B=-14.97, SE=6.07, 95% CI: [-27.08, -2.85], $p= 0.016$) and in ESC_{adj} serum concentrations (Model 2: B=1.00, SE=0.23, 95% CI: [0.54, 1.46], $p<0.001$) at Week 16. After controlling for ESC_{adj} serum concentrations, the

relationship between *CYP2C19* metabolizer group and total symptom improvement was no longer significant, whereas ESC_{adj} serum concentration was significantly associated with total symptom improvement (Model 3: B=-6.70, SE=3.19, 95% CI: [-13.08, -0.32], p=0.040), indicating a possible complete mediation. Additionally, results of the bootstrap procedure estimated a percent mediation of 45% (95% CI: [0.05, 1.71]) indicating that about half of the effect of *CYP2C19* IM+PM phenotype on symptom improvement may be mediated by ESC serum levels (**Figure S22C**). *CYP2D6* metabolizer groups were not significantly associated with symptom improvement in either Model 1 and 3. When covariates were included in the three models, the mediation effect was no longer observed, possibly due to insufficient sample size (n=69). Further, there was no mediation effect when S-DCT or S-DCT/ESC ratio was replaced as mediators in the models.

Given a significant association between presence of CNS side effects and *CYP2D6* metabolizer group was observed in both treatment arms, mediation analyses were conducted to investigate whether the association between CNS side effects and *CYP2D6* metabolizer group was mediated by ESC_{adj} serum levels using two regression models. In the first ordinary least squares regression model, higher ESC concentrations were significantly related to *CYP2D6* IM+PMs (B=0.92, SE=0.23, 95% CI: [0.46, 1.38], p<0.001) in ESC-Only, but not in ESC+ARI (B=-0.10, SE=0.33, 95% CI: [-0.75, 0.55], p=n.s.). In the second logistic regression model, which included *CYP2D6* metabolizer group and ESC_{adj} serum levels as predictors of the presence of CNS side effects, neither *CYP2D6* metabolizer group (B=-0.55, SE=0.35, 95% CI: [-1.23, 0.13], p=n.s.), nor ESC_{adj} serum levels (B=-0.05, SE=0.16, 95% CI: [-0.37, 0.27], p=n.s.) was significantly independently associated with the presence of CNS side effects in the ESC-Only treatment arm. In the ESC+ARI treatment arm, a possible mediation effect was observed with ESC_{adj} serum concentration significantly associated with the presence of CNS side effects (B=0.50, SE=0.19, 95% CI: [0.14, 0.86], p=0.007), whereas the relationship between *CYP2D6* metabolizer group and CNS side effects was no longer significant (B=0.55, SE=0.38, 95% CI: [-0.18, 1,29], p=n.s.). Furthermore, in this treatment arm, a possible mediation effect was also observed with the presence of CNS side effects significantly associated with ARI_{adj} serum concentration (B=0.05, SE=0.02, 95% CI: [0.09, 0.05], p=0.026), but not with *CYP2D6* metabolizer group (B=0.57, SE=0.36, 95% CI: [1.28, 0.57], p=n.s.). However, the bootstrap confidence intervals derived from 5000 samples indicated that the indirect effect coefficient was not significant (ESC-Only: B=-0.04, SE=0.92, p=n.s.; ESC+ARI: B=-0.06, SE=0.42, p=n.s.), which did not support the hypothesis that the relation between the presence of CNS side effects and *CYP2D6* metabolizer group is mediated by ESC_{adj} serum levels (or ARI_{adj} serum levels in ESC+ARI treatment arm [B=0.06, SE=0.45, p=n.s.]).

For the observed association between the presence of sexual side effects and CYP2D6 metabolizer group in the ESC+ARI treatment arm, subsequent mediation analyses did not demonstrate a mediation effect by ESC_{adj} or ARI_{adj} serum levels on this relationship.

Supplementary Discussions

No significant differences in serum ARI_{adj} and DHA_{adj} concentrations between *CYP2D6* NMs and IM+PMs were observed, while previous studies reported significantly higher ARI levels in IM+PMs than NMs when ARI is administered alone.[1–3] This provides further support for the phenoconversion of CYP2D6 by ESC and S-DCT, as well as competition by ESC and ARI for CYP2D6. These factors may have contributed to higher concentrations of ARI in NMs and IMs of the ESC+ARI treatment arm compared to when ARI is administered alone, thereby decreasing the difference in ARI concentration between NMs, IMs and PMs (**Figure S20**). Similarly, a previous study had shown that the coadministration of paroxetine or fluoxetine, both SSRI which compete for and inhibits CYP2D6, resulted in large interindividual variability in the systematic clearance of ARI, with NMs showing more variability and decreased systematic clearance than IM+PMs.[4]

In ESC+ARI, more *CYP2D6* IM+PMs reported the presence of sexual side effects compared to NMs during Phase II, specifically decreased libido. *Post-hoc* comparisons revealed that the presence of decreased libido was significantly different between IMs and NMs. Given that ESC has been reported to pose a higher risk of sexual dysfunction compared to other antidepressants,[5] it is possible that significant increases in serum levels of ESC in *CYP2D6* IMs, caused by decreased CYP2D6 activity, may negatively affect sexual functioning. However, when sexual side effect intensity was tested for correlations with serum concentrations of ESC_{adj}, S-DCT_{adj}, ARI_{adj}, DHA_{adj}, or their metabolite-to-drug ratio in this treatment arm, no significant correlations were observed. Mediation analyses also did not reveal a mediating effect by serum measures of drug on the relationship between CYP2D6 and sexual side effects. Therefore, this relationship and its underlying mechanisms needs to be further studied in a larger sample.

References

1 Hendset M, Hermann M, Lunde H, Refsum H, Molden E. Impact of the CYP2D6 genotype on steady-state serum concentrations of aripiprazole and dehydroaripiprazole. *Eur J Clin Pharmacol* 2007; **63**: 1147–1151.

2 Suzuki T, Mihara K, Nakamura A, Kagawa S, Nagai G, Nemoto K *et al.* Effects of Genetic Polymorphisms of CYP2D6, CYP3A5, and ABCB1 on the Steady-State Plasma Concentrations of Aripiprazole and Its Active Metabolite, Dehydroaripiprazole, in Japanese Patients With Schizophrenia. *Therapeutic Drug Monitoring* 2014; **36**: 651–655.

3 Jukic MM, Smith RL, Haslemo T, Molden E, Ingelman-Sundberg M. Effect of CYP2D6 genotype on exposure and efficacy of risperidone and aripiprazole: a retrospective, cohort study. *The Lancet Psychiatry* 2019; **6**: 418–426.

4 Azuma J, Hasunuma T, Kubo M, Miyatake M, Koue T, Higashi K *et al.* The relationship between clinical pharmacokinetics of aripiprazole and CYP2D6 genetic polymorphism: effects of CYP enzyme inhibition by coadministration of paroxetine or fluvoxamine. *Eur J Clin Pharmacol* 2012; **68**: 29–37.

5 Reichenpfader U, Gartlehner G, Morgan LC, Greenblatt A, Nussbaumer B, Hansen RA *et al.* Sexual dysfunction associated with second-generation antidepressants in patients with major depressive disorder: results from a systematic review with network meta-analysis. *Drug Saf* 2014; **37**: 19–31.

Table S1. Items on the Toronto Side Effects Scale grouped into four categories.

	Central Nervous System	Gastrointestinal System	Sexual Functioning	Weight gain
1.	Agitation	Abdominal pain	Anorgasmia	Weight gain
2.	Blurred vision	Constipation	Decreased libido	
3.	Decreased sleepiness	Decreased appetite	Increased libido	
4.	Drowsiness	Diarrhea		
5.	Dizziness	Dry mouth		
6.	Flushing	Dyspepsia		
7.	Headache	Edema		
8.	Increased sleepiness	Increased appetite		
9.	Nervousness	Nausea		
10.	Postural hypotension			
11.	Sweating			
12.	Tremor			
13.	Twitching myoclonus			
14.	Weakness and fatigue			

Table S2. Description of human *CYP2D6* and *CYP2C19* gene variants genotyped in this study.

Allele	Predicted Activity[a]
CYP2C19	
*1	Normal
*2	Non-functional
*3	Non-functional
*17	Increased
CYP2D6	
*1	Normal
*2	Normal
*3	Non-functional
*4	Non-functional
*5	Non-functional
*6	Non-functional
*9	Reduced
*10	Reduced
*17	Reduced
*36	Reduced
*41	Reduced
*2 (xN)	Increased
*36	Reduced
*4 (xN)	Non-functional
*10 (xN)	Reduced
*9 (xN)	Reduced
*41 (xN)	Reduced

[a]Data extracted from SNPedia.com and consistent with guidelines by Clinical Pharmacogenetics Implementation Consortium (CPIC®).

Table S3. Distribution of *CYP2C19* and *CYP2D6* genotypes and predicted metabolizer status.

Metabolizer Status	Frequency (*n,* %) Sample N=178	ESC N=81	ESC+ARI N=97	Age (mean ± SD)	Genotypes (*n*)
		CYP2C19			
NM	71 (39.89)	33 (40.74)	38 (39.18)	35.04 ± 12.96	*1/*1 (71)
IM	51 (28.65)	22 (27.16)	29 (29.90)	35.67 ± 12.74	*1/*2 (39)<br>*2/*17 (10)<br>*1/*3 (1)<br>*3/*17 (1)
PM	5 (2.809)	3 (3.704)	2 (2.062)	34.20 ± 14.20	*2/*2 (3)<br>*2/*3 (2)
RM	43 (24.16)	21(25.93)	22 (22.68)	35.91 ± 13.08	*1/*17 (43)
UM	7 (3.933)	1 (1.235)	6 (6.186)	36.14 ± 12.54	*17/*17 (7)
Not known	1 (0.57)	1 (1.235)	0 (0)	-	-
		CYP2D6			
NM	99 (55.62)	41 (50.61)	58 (59.79)	36.61 ± 13.18	*1/*2 (27)<br>*1/*1 (22)<br>*2/*4 (10)<br>*2/*41 (9)<br>*2/*2 (7)<br>*1/*41 (5)<br>*1/*36+*10 (4)<br>*2/*5 (4)<br>*1/*9 (3)<br>*1/*17 (2)<br>*1/*10 (1)<br>*2/*3 (1)<br>*2/*36+*4 (1)<br>*2/*9 (1)<br>*2/*9 (xN) (1)<br>*2/*17 (1)
IM	60 (33.71)	30 (37.04)	30 (30.93)	33.70 ± 12.86	*1/*4 (23)<br>*1/*5 (8)<br>*36+10/*36 (5)<br>*1/*4 (xN) (3)<br>*10/*36+10 (3)<br>*41/*41 (3)<br>*1/*3 (2)<br>*4/*41 (2)<br>*4/*10 (2)<br>*2/*4 (xN) (1)<br>*4/*9 (1)<br>*4/*36+*10 (1)<br>*5/*9 (1)<br>*5/*36+*10 (1)<br>*6/*41 (1)<br>*9/*9 (1)

					*10/*41 (1)<br>*36+*10/*41 (1)
PM	11 (6.18)	5 (6.173)	6 (6.186)	38.45 ± 10.14	*4/*4 (7)<br>*4/*5 (2)<br>*4/*6 (1)<br>*5/*6 (1)
UM*	2 (1.124)	2 (2.469)	0 (0)	35.50 ± 14.85	*1/*2(xN) (1)<br>*2/*2(xN) (1)
Not known	6 (3.371)	3 (3.704)	3 (3.093)	27.67 ± 3.78	-

**Note*: UMs for CYP2D6 were excluded from analyses due to the small sample size (n=2).
IM = Intermediate Metabolizer; NM = Normal Metabolizer; PM = Poor Metabolizer; RM = Rapid Metabolizer; SD = Standard error; UM = Ultrarapid Metabolizer.

Figure S1. Symptom improvement and ESC_{adj} serum concentrations at Week 2 by *CYP2C19* and *CYP2D6* metabolizer phenotypes for Phase I dropouts.

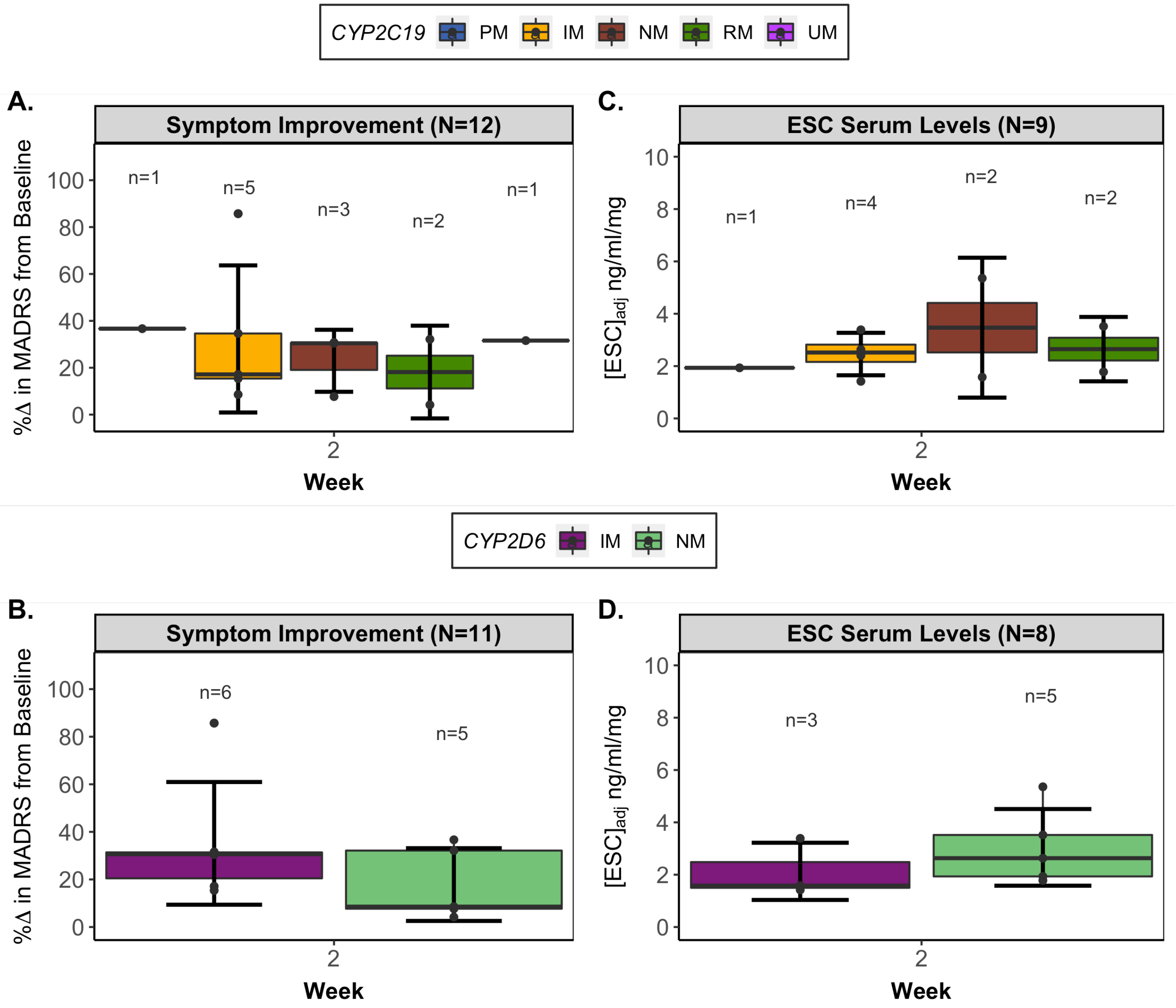

There were no differences in (**A-B**) symptom improvement (*CYP2C19*: H(4)=2.63, *p*=0.621; *CYP2D6*: H(1)=0.83, p=0.361) or (**C-D**) ESC serum concentrations (*CYP2C19*: H(3)=0.30, p=0.960; *CYP2D6*: H(1)=1.80, p=0.180) at Week 2 between *CYP2C19* and *CYP2D6* metabolizer phenotypes in dropouts for whom data was available. Error bars represent standard error.
ARI = Aripiprazole; ESC = Escitalopram; IM = Intermediate Metabolizer; NM = Normal Metabolizer; PM = Poor Metabolizer; RM = Rapid Metabolizer; UM = Ultra-rapid Metabolizer.

Table S4. Basic sample demographics and clinical information for the European subset.

Characteristics	All	*CYP2C19*				*CYP2D6*		
	**N = 129**	**NM (N = 57)**	**IM+PM (N = 29)**	**RM+UM (N = 42)**	***p*-value[1]**	**NM (N = 75)**	**IM+PM (N = 51)**	***p*-value[2]**
Age	36.25 (13.12)	35.47 (13.24)	37.48 (13.16)	36.57 (13.31)	0.715	37.15 (13.61)	35.49 (12.58)	0.507
Sex					0.404			0.924
Female	74 (57%)	29 (51%)	17 (59%)	27 (64%)		42 (56%)	29 (57%)	
Male	55 (43%)	28 (49%)	12 (41%)	15 (36%)		33 (44%)	22 (43%)	
Previous AD treatment for current MDE					0.152			0.295
None	74 (57%)	29 (51%)	21 (72%)	23 (55%)		40 (53%)	32 (63%)	
1+	55 (43%)	28 (49%)	8 (28%)	19 (45%)		35 (47%)	19 (37%)	
ESC Dose at Week 8					0.227			>0.999
10 mg	128 (99%)	57 (100%)	28 (97%)	42 (100%)		74 (99%)	51 (100%)	
20 mg	1 (0.8%)	0 (0%)	1 (3.4%)	0 (0%)		1 (1.3%)	0 (0%)	
ESC Dose at Week 16					0.899			**0.007 ****
10 mg	11 (9.2%)	4 (7.4%)	3 (12%)	4 (10%)		2 (2.9%)	9 (19%)	
15 mg	1 (0.8%)	1 (1.9%)	0 (0%)	0 (0%)		1 (1.4%)	0 (0%)	
20 mg	108 (90%)	49 (91%)	23 (88%)	35 (90%)		67 (96%)	38 (81%)	
Phase II Treatment Arm					0.339			0.152
ESC	58 (45%)	29 (51%)	10 (34%)	18 (43%)		30 (40%)	27 (53%)	
ESC+ARI	71 (55%)	28 (49%)	19 (66%)	24 (57%)		45 (60%)	24 (47%)	
Baseline MADRS Score	29.83 (5.25)	30.05 (5.29)	29.38 (4.78)	29.79 (5.63)	0.911	30.69 (5.56)	28.63 (4.62)	**0.043 ***
CYP2C19 Metabolizer Groups					-			0.700
NM	57 (45%)	-	-	-		35 (47%)	20 (39%)	
IM+PM	29 (23%)	-	-	-		16 (21%)	13 (25%)	
RM+UM	42 (33%)	-	-	-		24 (32%)	18 (35%)	
CYP2D6 Metabolizer Groups					0.700			-
NM	75 (60%)	35 (64%)	16 (55%)	24 (57%)		-	-	
IM+PM	51 (40%)	20 (36%)	13 (45%)	18 (43%)		-	-	

[1]Kruskal-Wallis rank sum test; Pearson's Chi-squared test; Fisher's exact test

[2]Wilcoxon rank sum test; Pearson's Chi-squared test; Fisher's exact test

AD = Antidepressant; ARI = Aripiprazole; ESC = Escitalopram; IM = Intermediate Metabolizer; MADRS = Montgomery–Åsberg Depression Rating Scale; MDE = Major Depressive Episode; NM = Normal Metabolizer; PM = Poor Metabolizer; RM = Rapid Metabolizer; SD = Standard error; UM = Ultra-rapid Metabolizer.

* p<0.05; ** p<0.01; ***p<0.001

Table S5. Summary of logistic regression models for response and remission status at the end of Phase I and II adjusted for adjusted for age, ancestry, recruitment site, sex, and total MADRS score at baseline as fixed effects.

	Phase I				*Phase II: ESC-Only*				*Phase II: ESC+ARI*			
**Independent Variables**	**Odds Ratios**	**SE**	**CI (95%)**	***P*-value**	**Odds Ratios**	**SE**	**CI (95%)**	***P*-value**	**Odds Ratios**	**SE**	**CI (95%)**	***P*-value**
						**Response Status**						
**Intercept**	2.15	2.92	0.15 – 30.76	0.572	34.90	128.83	0.03 – 48380.55	0.336	0.25	0.51	0.00 – 14.01	0.497
**CYP2C19 RM+UM**	0.78	0.30	0.36 – 1.66	0.514	0.41	0.67	0.02 – 9.97	0.588	0.63	0.39	0.19 – 2.09	0.451
**CYP2C19 IM+PM**	0.97	0.38	0.46 – 2.08	0.947	0.45	0.66	0.03 – 7.89	0.587	0.77	0.49	0.22 – 2.65	0.679
**CYP2D6 IM+PM**	1.13	0.36	0.60 – 2.12	0.713	0.39	0.41	0.05 – 3.09	0.376	0.90	0.48	0.31 – 2.58	0.845
Observations	170				71				82			
R^2	0.031				0.408				0.165			
AIC	256.823				56.451				122.088			
						**Remission Status**						
**Intercept**	2.95	4.77	0.12 – 70.42	0.505	14.59	38.16	0.09 – 2457.95	0.306	3.98	7.83	0.08 – 187.77	0.482
**CYP2C19 RM+UM**	0.89	0.38	0.38 – 2.08	0.791	0.42	0.38	0.07 – 2.47	0.339	0.87	0.51	0.28 – 2.73	0.814
**CYP2C19 IM+PM**	1.13	0.49	0.48 – 2.66	0.774	0.66	0.55	0.13 – 3.37	0.616	1.38	0.80	0.45 – 4.27	0.576
**CYP2D6 IM+PM**	1.26	0.45	0.63 – 2.54	0.513	1.35	0.91	0.36 – 5.09	0.660	0.64	0.32	0.24 – 1.73	0.379
Observations	170				71				82			

R^2	0.106	0.230	0.096
AIC	220.477	87.822	132.803

There were no significant impacts of either *CYP2C19* or *CYP2D6* metabolizer groups on response status (responder vs. non-responder) or remission status (remitter vs. non-remitter) at the end of Phase I or Phase II.
AIC=Akaike information criterion; ARI=Aripiprazole; CI=confidence interval; ESC=Escitalopram; IM = Intermediate Metabolizer; NM = Normal Metabolizer; PM = Poor Metabolizer; RM = Rapid Metabolizer; SE=standard error; UM = Ultra-rapid Metabolizer.
*p<0.05 ** p<0.01 *** p<0.001

Table S6. Summary of logistic regression models for response and remission status at the end of Phase I and II for the European subset, adjusted for age, ancestry, recruitment site, sex, and total MADRS score at baseline as fixed effects.

	Phase I				*Phase II: ESC-Only*				*Phase II: ESC+ARI*			
**Independent Variables**	**Odds Ratios**	**SE**	**CI (95%)**	**P-Value**	**Odds Ratios**	**SE**	**CI (95%)**	**P-Value**	**Odds Ratios**	**SE**	**CI (95%)**	**P-Value**
**Response Status**												
**Intercept**	2.52	3.39	0.18 - 35.32	0.494	4.02×10^3	2.01×10^4	$0.22 – 7.27 \times 10^7$	0.097	6.81	18.60	$0.03 – 1.44 \times 10^3$	0.482
**CYP2C19 RM+UM**	0.83	0.32	0.39 – 1.75	0.620	0.24	0.36	0.01 – 4.68	0.346	0.50	0.42	0.10 – 2.60	0.410
**CYP2C19 IM+PM**	1.09	0.43	0.50 – 2.37	0.820	0.73	1.14	0.03 – 15.32	0.841	1.03	0.79	0.23 – 4.60	0.971
**CYP2D6**	1.13	0.37	0.59 – 2.15	0.720	0.34	0.46	0.02 – 4.79	0.422	0.26	0.19	0.06 – 1.09	0.065
Observations	163				53				63			
R^2	0.033				0.134				0.347			
AIC	245.634				34.486				87.267			
**Remission Status**												
**Intercept**	3.28	5.28	0.14 – 77.30	0.462	6.05×10^2	1.82×10^3	$1.66 – 2.20 \times 10^5$	**0.033 ***	4.07×10^2	1.12×10^3	$1.85 – 9.00 \times 10^4$	**0.029 ***
**CYP2C19 RM+UM**	0.98	0.41	0.43 – 2.24	0.956	0.42	0.46	0.05 – 3.66	0.432	1.93	1.55	0.40 – 9.34	0.414
**CYP2C19 IM+PM**	1.19	0.53	0.50 – 2.83	0.689	1.01	0.95	0.16 – 6.41	0.991	2.47	1.78	0.60 – 10.15	0.209
**CYP2D6**	1.22	0.44	0.60 – 2.48	0.584	1.90	1.58	0.37 – 9.69	0.438	0.20	0.14	0.05 – 0.79	**0.021 ***
Observations	163				53				63			
R^2	0.118				0.197				0.325			

AIC	212.217	55.656	93.392

There were no significant impacts of either *CYP2C19* or *CYP2D6* metabolizer groups on response status (responder vs. non-responder) or remission status (remitter vs. non-remitter) at the end of Phase I or Phase II for Europeans.

AIC = Akaike information criterion; ARI = Aripiprazole; CI = confidence interval; ESC = Escitalopram; IM = Intermediate Metabolizer; NM = Normal Metabolizer; PM = Poor Metabolizer; RM = Rapid Metabolizer; SE = standard error; UM = Ultra-rapid Metabolizer.

*$p<0.05$ ** $p<0.01$ *** $p<0.001$

Figure S2. Symptom improvement over time for Phase I and II by ungrouped *CYP2C19* and *CYP2D6* metabolizer phenotypes.

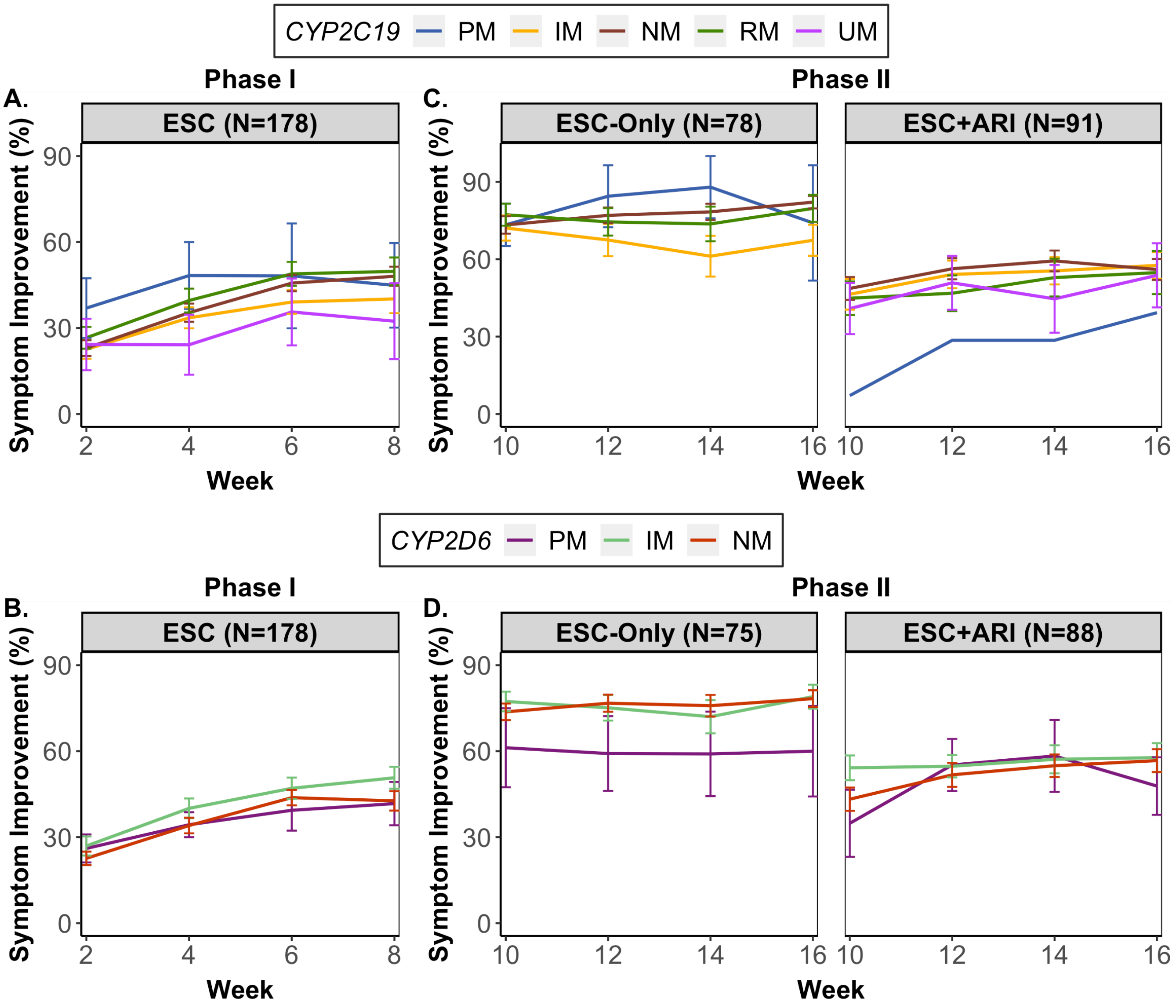

Descriptive plots of change in total MADRS scores from baseline by ungrouped *CYP2C19* and *CYP2D6* metabolizer phenotypes during Phases I and II. Error bars represent standard error.
ARI = Aripiprazole; ESC = Escitalopram; IM = Intermediate Metabolizer; NM = Normal Metabolizer; PM = Poor Metabolizer; RM = Rapid Metabolizer; UM = Ultra-rapid Metabolizer.

Table S7. Summary of mixed effects models for symptom improvement during Phase I and Phase II adjusted for age, ancestry, and sex as fixed effects, and recruitment site and individual as random effects variables.

	Phase I					*Phase II: ESC-Only*					*Phase II: ESC+ARI*				
**Fixed Effects**	**Estimates**	**SE**	**CI (95%)**	**P-Value**	**df**	**Estimates**	**SE**	**CI (95%)**	**P-Value**	**df**	**Estimates**	**SE**	**CI (95%)**	**P-Value**	**df**
**Intercept**	21.19	8.29	4.90 – 37.48	**0.011** *	505	58.69	13.49	32.10 – 85.29	**<0.001** ***	207	42.21	14.02	14.60 – 69.81	**0.003** **	239
**Week**	4.14	0.51	3.13 – 5.14	**<0.001** ***	505	1.47	0.61	0.26 – 2.67	**0.017** *	60	2.00	0.57	0.88 – 3.12	**0.001** ***	70
**CYP2C19 IM+PM**	3.27	5.64	-7.87 – 14.41	0.563	158	26.15	12.71	0.73 – 51.56	**0.044** *	60	-7.04	12.01	-31.00 – 16.92	0.560	70
**CYP2C19 RM+UM**	3.74	5.71	-7.53 – 15.01	0.513	158	14.10	12.37	-10.66 – 38.85	0.259	60	-5.02	12.17	-29.28 – 19.24	0.681	70
**CYP2D6 IM+PM**	3.13	4.72	-6.18 – 12.44	0.508	158	1.19	10.27	-19.35 – 21.73	0.908	207	19.09	10.36	-1.57 – 39.75	0.070	239
**CYP2C19 IM+PM*Week**	-1.48	0.69	-2.84 – -0.12	**0.033** *	505	-2.34	0.85	-4.02 – -0.66	**0.007** **	207	0.45	0.77	-1.08 – 1.97	0.565	239
**CYP2C19 RM+UM*Week**	-0.73	0.71	-2.13 – 0.67	0.306	505	-1.04	0.86	-2.73 – 0.64	0.223	207	0.13	0.78	-1.41 – 1.67	0.865	239
**CYP2D6 IM+PM*Week**	0.31	0.59	-0.85 – 1.47	0.595	505	-0.41	0.71	-1.81 – 0.99	0.566	207	-1.29	0.67	-2.60 – 0.02	0.054	239
**Random Effects**															
N			170 SUBJLABEL					72 SUBJLABEL					82 SUBJLABEL		
			6 SITESYMBOL					6 SITESYMBOL					6 SITESYMBOL		
Observations			679					283					325		

AIC = Akaike information criterion; ARI = Aripiprazole; CI = confidence interval; ESC = Escitalopram; IM = Intermediate Metabolizer; NM = Normal Metabolizer; PM = Poor Metabolizer; RM = Rapid Metabolizer; SE = standard error; UM = Ultra-rapid Metabolizer.
*p<0.05 ** p<0.01 *** p<0.001

Table S8. Summary of mixed effects models for symptom improvement during Phase I and Phase II for the European subset, adjusted for age, ancestry, and sex as fixed effects, and recruitment site and individual as random effects variables.

	Phase I					*Phase II: ESC-Only*					*Phase II: ESC+ARI*				
**Fixed Effects**	**Estimates**	**SE**	**CI (95%)**	**P-Value**	**df**	**Estimates**	**SE**	**CI (95%)**	**P-Value**	**df**	**Estimates**	**SE**	**CI (95%)**	**P-Value**	**df**
**Intercept**	28.33	9.17	10.29 – 46.36	**0.002 ****	373	59.39	13.71	32.32 – 86.47	**<0.001 *****	155	41.32	14.70	12.32 – 70.31	**0.005 ****	183
**Week**	4.16	0.56	3.05 – 5.27	**<0.001 *****	373	1.76	0.61	0.55 – 2.97	**0.005** **	43	2.25	0.63	1.00 – 3.51	**<0.001 *****	52
**CYP2C19 IM+PM**	-0.36	6.81	-13.84 – 13.12	0.958	115	25.57	15.73	-6.15 – 57.30	0.111	43	6.44	13.91	-21.47 – 34.35	0.645	52
**CYP2C19 RM+UM**	1.40	6.09	-10.67 – 13.47	0.819	115	13.26	11.95	-10.84 – 37.35	0.273	155	-3.23	13.17	-29.66 – 23.20	0.807	183
**CYP2D6 IM+PM**	5.90	5.39	-4.77 – 16.58	0.276	115	6.63	10.74	-15.02 – 28.28	0.540	43	15.32	11.74	-8.24 – 38.88	0.198	52
**CYP2C19 IM+PM*Week**	-1.29	0.86	-2.98 – 0.41	0.136	373	-2.27	1.08	-4.40 – -0.13	**0.038 ***	155	-0.10	0.92	-1.91 – 1.70	0.911	183
**CYP2C19 RM+UM*Week**	-0.66	0.77	-2.18 – 0.85	0.389	373	-0.88	0.82	-2.49 – 0.74	0.285	155	0.35	0.87	-1.37 – 2.06	0.690	183
**CYP2D6 IM+PM*Week**	0.19	0.68	-1.15 – 1.53	0.785	373	-0.64	0.74	-2.10 – 0.81	0.384	155	-1.60	0.77	-3.13 – -0.07	**0.040 ***	183
**Random Effects**															
N	126 SUBJLABEL					54 SUBJLABEL					63 SUBJLABEL				
	6 SITESYMBOL					6 SITESYMBOL					6 SITESYMBOL				
Observations	503					213					250				

AIC = Akaike information criterion; ARI = Aripiprazole; CI = confidence interval; ESC = Escitalopram; IM = Intermediate Metabolizer; NM = Normal Metabolizer; PM = Poor Metabolizer; RM = Rapid Metabolizer; SE = standard error; UM = Ultra-rapid Metabolizer.
p*<0.05 ** *p*<0.01 * *p*<0.001

Table S9. Summary of logistic regression models assessing associations of the presence or absence of central nervous system, gastrointestinal, and sexual side effects, and treatment-related weight changes with *CYP2C19* and *CYP2D6* metabolizer groups during Phase I and II, adjusted for age, ancestry, sex, recruitment site, and total MADRS score at baseline.

	Phase I					***Phase II: ESC-Only***					***Phase II: ESC+ARI***				
**Fixed Effects**	**Odds Ratios**	**SE**	**CI (95%)**	**P-Value**	**Summary**	**Odds Ratios**	**SE**	**CI (95%)**	**P-Value**	**Summary**	**Odds Ratios**	**SE**	**CI (95%)**	**P-Value**	**Summary**
**Central Nervous System (CNS) Side Effects**															
**CYP2C19 IM+PM**	0.95	0.44	0.38 – 2.37	0.909	N=169 R^2=0.189 AIC=183.793	0.82	0.66	0.17 – 4.02	0.803	N=70 R^2=0.472 AIC=91.731	1.51	1.38	0.25 – 9.07	0.651	N=82 R^2=0.470 AIC=76.128
**CYP2C19 RM+UM**	1.35	0.68	0.50 – 3.61	0.554		1.10	0.86	0.24 – 5.13	0.903		1.99	1.87	0.32 – 12.51	0.461	
**CYP2D6 IM+PM**	0.76	0.30	0.35 – 1.65	0.481		0.13	0.10	0.03 – 0.61	**0.010 ****		11.52	10.88	1.81 – 73.35	**0.010 ****	
**Gastrointestinal Side Effects**															
**CYP2C19 IM+PM**	0.59	0.25	0.26 – 1.33	0.204	N=168 R^2=0.159 AIC=220.798	0.20	0.18	0.03 – 1.18	0.076	N=71 R^2=0.327 AIC=99.89	1.02	0.63	0.31 – 3.42	0.971	N=82 R^2=0.239 AIC=121.283
**CYP2C19 RM+UM**	1.02	0.45	0.42 – 2.44	0.968		0.78	0.55	0.20 – 3.10	0.729		1.00	0.63	0.29 – 3.41	0.996	
**CYP2D6 IM+PM**	1.09	0.39	0.55 – 2.19	0.801		0.86	0.53	0.26 – 2.89	0.805		0.72	0.39	0.25 – 2.08	0.545	
**Sexual Side Effects**															
**CYP2C19 IM+PM**	1.69	0.70	0.75 – 3.81	0.203	N=167 R^2=0.071 AIC=239.312	4.39	3.91	0.77 – 25.20	0.097	N=71 R^2=0.152 AIC=96.641	2.86	2.08	0.69 – 11.88	0.149	N=81 R^2=0.259 AIC=106.338
**CYP2C19 RM+UM**	1.74	0.70	0.78 – 3.85	0.174		1.18	0.93	0.25 – 5.54	0.830		2.10	1.45	0.55 – 8.10	0.279	
**CYP2D6 IM+PM**	1.66	0.56	0.86 – 3.23	0.132		0.58	0.36	0.17 – 1.96	0.377		6.72	4.46	1.83 – 24.67	**0.004 ****	
**Treatment-Related Weight Gain**															
**CYP2C19 IM+PM**	3.21	1.96	0.97 – 10.62	**0.056**	N=167 R^2=0.117	2.93	3.81	0.23 – 37.29	0.406	N=71 R^2=0.223	1.07	0.65	0.32 – 3.52	0.917	N=81 R^2=0.145

**CYP2C19 RM+UM**	1.83	1.21	0.50 – 6.67	0.359	AIC=145.252	1.75	1.94	0.20 – 15.40	0.614	AIC=67.50 1	1.10	0.65	0.34 – 3.52	0.876	AIC=125.504
**CYP2D6 IM+PM**	0.95	0.47	0.36 – 2.50	0.911		0.49	0.44	0.08 – 2.84	0.426		0.65	0.35	0.23 – 1.85	0.420	

AIC = Akaike information criterion; ARI = Aripiprazole; CI = confidence interval; ESC = Escitalopram; IM = Intermediate Metabolizer; NM = Normal Metabolizer; PM = Poor Metabolizer; RM = Rapid Metabolizer; SE = standard error; UM = Ultra-rapid Metabolizer.
*$p<0.05$ ** $p<0.01$ *** $p<0.001$

Figure S3. Subcategories of CNS side effects that were significantly associated with *CYP2D6* metabolizer groups at Week 16 by treatment arm.

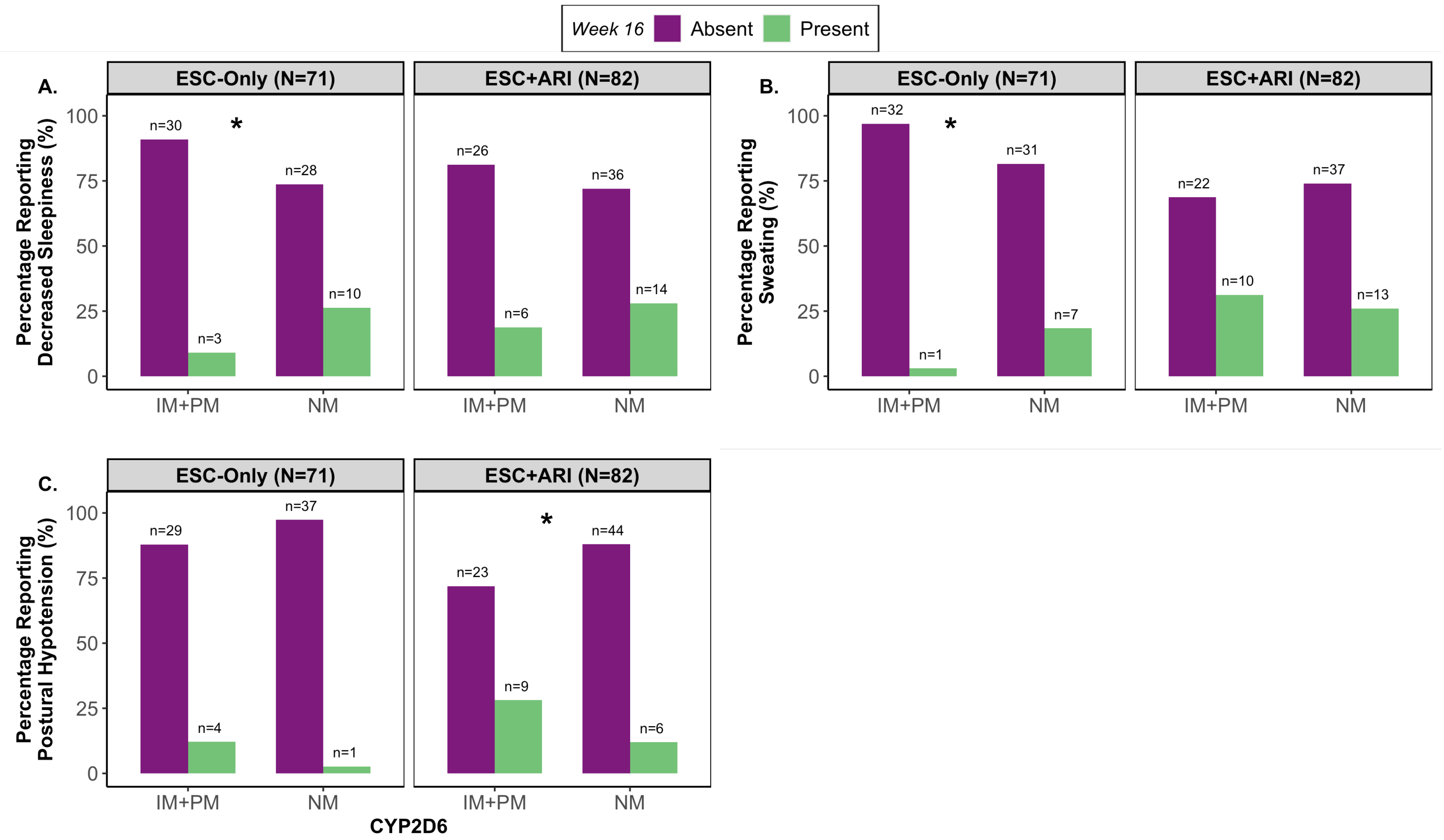

(A) The presence of decreased sleepiness was influenced by *CYP2D6* metabolizer groups in the ESC-Only treatment arm. The odds of reporting decreased sleepiness as a side effect was 25.53 (SE=33.30, 95% [CI 1.99, 328.18]) times higher for NMs compared to IM+PMs ($\chi 2$ (1, N = 71) = 6.18, p=0.013). **(B)** The presence of sweating was influenced by *CYP2D6* metabolizer groups in the ESC-Only treatment arm. The odds of reporting sweating as a side effect was 29.21 (SE=46.80, 95% CI [1.26, 676.37]) times higher for NMs compared to IM+PMs ($\chi 2$ (1, N = 71) = 4.43, p=0.035). **(A)** The presence of postural hypotension was influenced by *CYP2D6* metabolizer groups in the ESC+ARI treatment arm. The odds of reporting postural hypotension as a side effect was 8.07 (SE=6.89, 95% [CI 1.51, 43.04]) times higher for IM+PMs compared to NMs ($\chi 2$ (1, N = 82) = 5.98, p=0.014).

All logistic regression analyses were adjusted for adjusted for age, ancestry, sex, recruitment site, total MADRS score at baseline, and *CYP2D6* metabolizer groups.

ESC = Escitalopram; IM = Intermediate Metabolizer; NM = Normal Metabolizer; PM = Poor Metabolizer.

* p<0.05

Figure S4. Subcategories of CNS side effects that were significantly associated with ungrouped *CYP2D6* metabolizer groups at Week 16 by treatment arm.

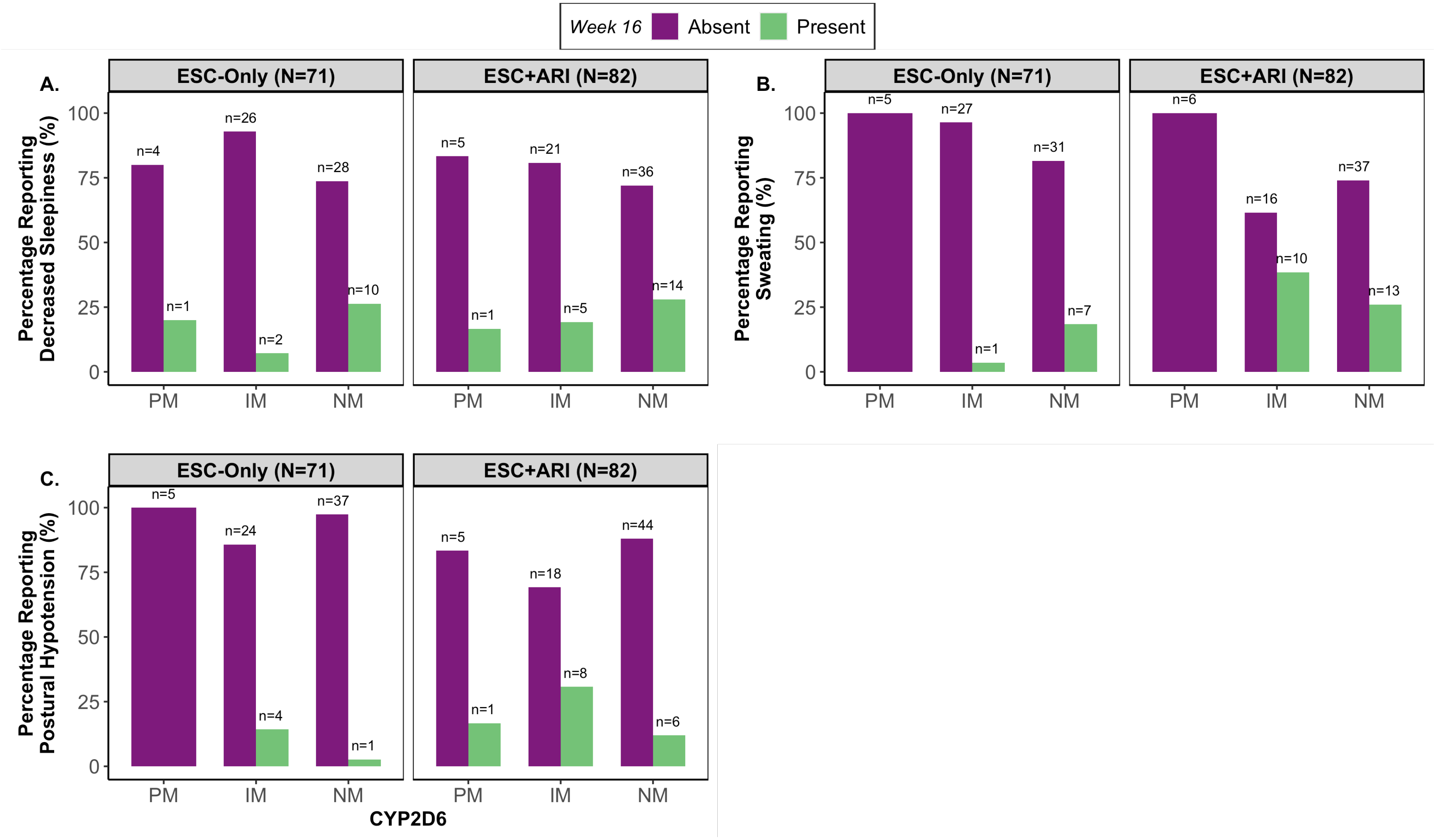

At Week 16, the likelihood of reporting **(A)** decreased sleepiness or **(B)** sweating were not significantly different between *CYP2D6* NMs, IMs and PMs, when the metabolizer phenotypes are assessed separately for either treatment arm. **(C)** *CYP2D6* IMs trended towards a 3.26 (95% CI [0.99, 10.74]) higher odds of reporting the presence of postural hypotension compared to NMs ($\chi2(1, N=81)=5.93$, $p=0.015$ for only the ESC+ARI treatment arm.
ARI = Aripiprazole; ESC = Escitalopram; IM = Intermediate Metabolizer; NM = Normal Metabolizer; PM = Poor Metabolizer.
# indicates trend with *p* between 0.050 to 0.070.

Figure S5. Sexual side effects self-reported to be present or absent by *CYP2C19* and *CYP2D6* metabolizer groups and treatment arm.

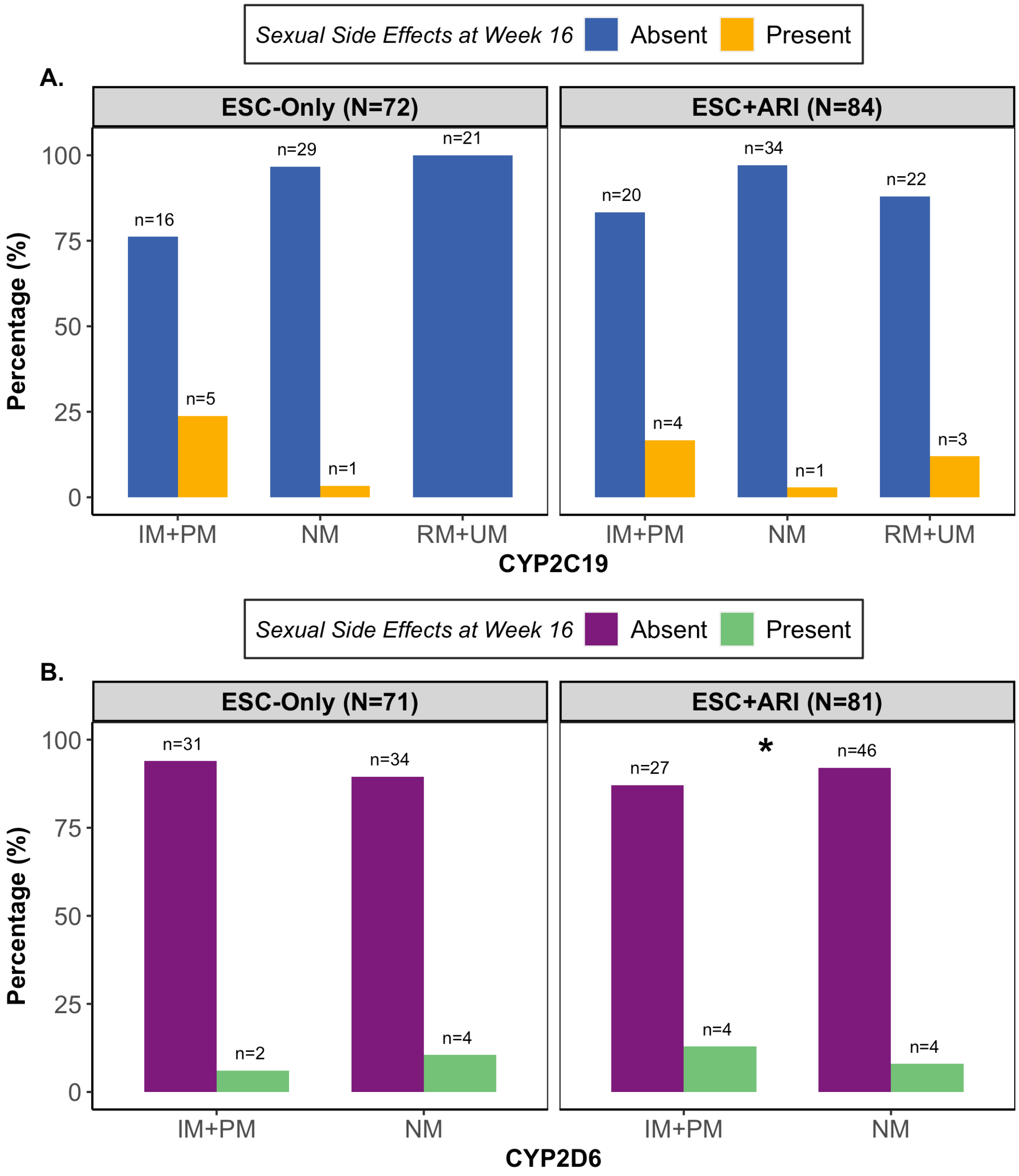

(A) Sexual (CNS) side effects did not show an association with *CYP2C19* metabolizer groups. **(B)** Presence of sexual side effects showed a trend for an association with *CYP2D6* metabolizer groups. In ESC+ARI, the odds of reporting a sexual side effect was 6.72 (95% CI 1.83, 24.67) times higher for IM+PMs compared to NMs ($\chi 2$ (1, N = 81) = 8.26, p=0.004, q=0.046). The ESC-Only treatment arm did not show the same effect.

All logistic regression analyses were adjusted for adjusted for age, ancestry, sex, recruitment site, total MADRS score at baseline, *CYP2C19* and *CYP2D6* metabolizer groups. *P*-values are corrected for multiple testing using the false discovery rate (FDR) approach.

ARI = Aripiprazole; ESC = Escitalopram; IM = Intermediate Metabolizer; NM = Normal Metabolizer; PM = Poor Metabolizer; RM = Rapid Metabolizer; UM = Ultra-rapid Metabolizer.

* $q < 0.05$

Figure S6. Subcategory of sexual side effects that were significantly associated with *CYP2D6* metabolizer groups at Week 16 for the ESC+ARI treatment arm.

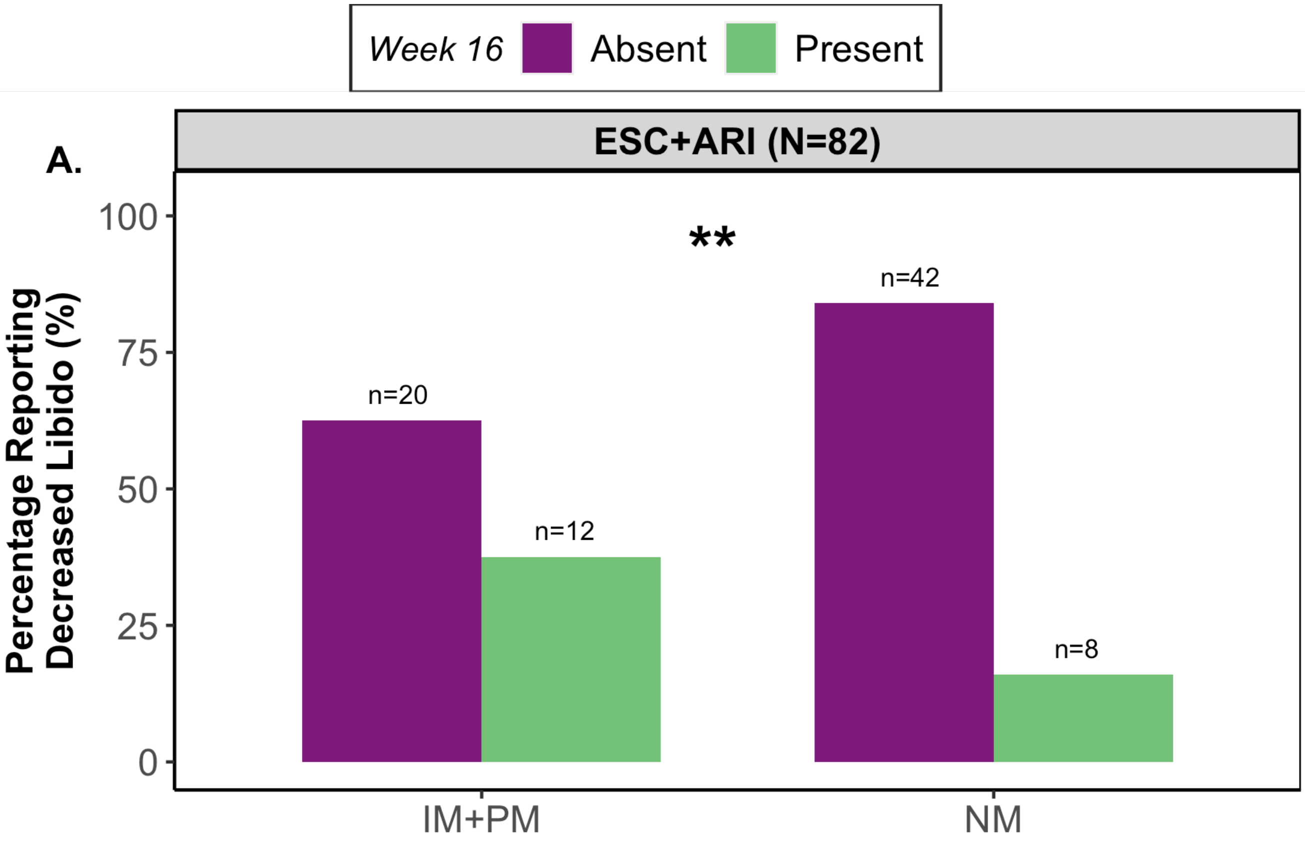

(A) The presence of decreased libido was influenced by *CYP2D6* metabolizer groups in the ESC+ARI treatment arm. The odds of reporting decreased sleepiness as a side effect was 9.63 (95% CI [1.97, 47.04]) times higher for IM+PMs compared to NMs ($\chi 2$ (1, N = 82) = 7.84, p=0.005).
All logistic regression analyses were adjusted for adjusted for age, ancestry, sex, recruitment site, total MADRS score at baseline, and *CYP2D6* metabolizer groups.
ARI = Aripiprazole; ESC = Escitalopram; IM = Intermediate Metabolizer; NM = Normal Metabolizer; PM = Poor Metabolizer; RM = Rapid Metabolizer; UM = Ultra-rapid Metabolizer
* $p<0.05$

Figure S7. Sexual side effects and decreased libido self-reported to be present or absent at Week 16 by ungrouped *CYP2D6* metabolizer phenotypes for the ESC+ARI treatment arm.

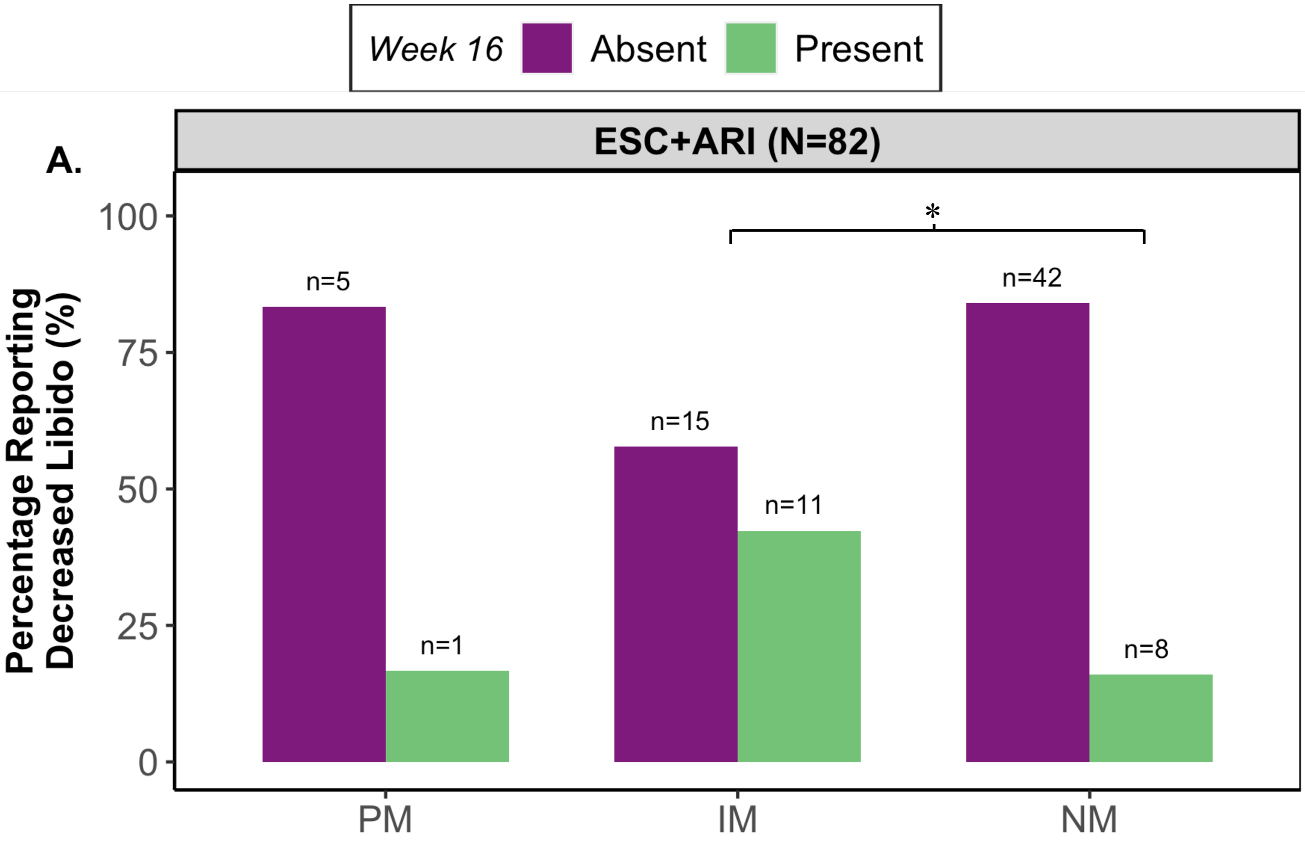

(A) At Week 16, *CYP2D6* IMs had 3.27 (95% CI [1.17, 9.18]) higher odds of reporting the presence of sexual side effects compared to NMs ($\chi 2(1, N=81)=5.93$, $p=0.015$), specifically decreased libido (OR=3.85, 95% CI [1.30, 11.39]), while the likelihood of reporting a sexual side effect was not significantly different between NMs and PMs for ESC+ARI treatment arm.
ARI = Aripiprazole; ESC = Escitalopram; IM = Intermediate Metabolizer; NM = Normal Metabolizer; PM = Poor Metabolizer.

Figure S8. Gastrointestinal side effects self-reported to be present or absent by *CYP2C19* and *CYP2D6* metabolizer groups and treatment arm.

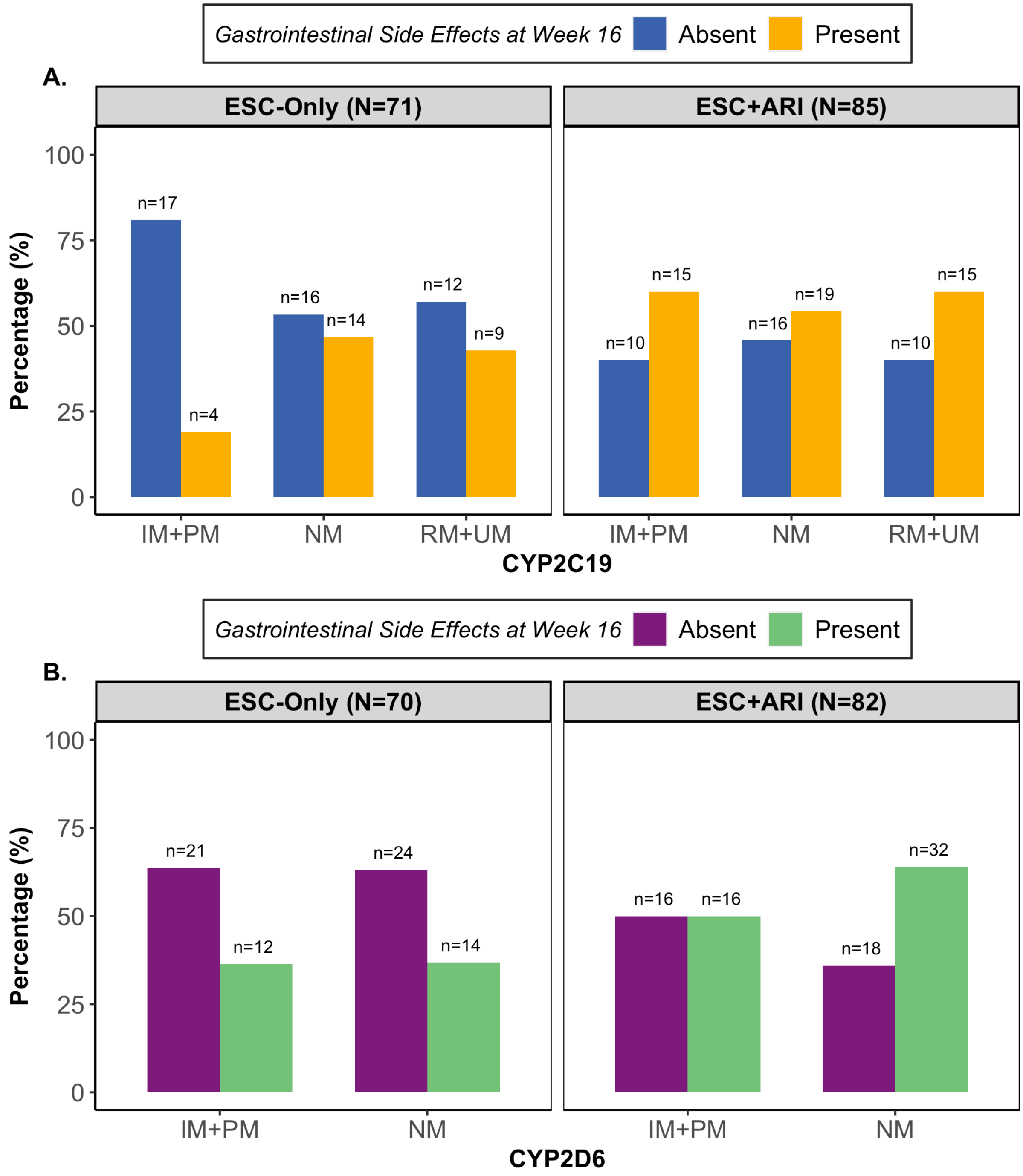

No associations between the presence of gastrointestinal side effects at trial end with either *CYP2C19* or *CYP2D6* metabolizer groups were observed.
All logistic regression analyses were adjusted for adjusted for age, ancestry, sex, recruitment site, total MADRS score at baseline, and *CYP2C19* and *CYP2D6* metabolizer groups.
ARI = Aripiprazole; ESC = Escitalopram; IM = Intermediate Metabolizer; NM = Normal Metabolizer; PM = Poor Metabolizer; RM = Rapid Metabolizer; UM = Ultra-rapid Metabolizer.

Figure S9. Treatment-related weight gain self-reported to be present or absent by *CYP2C19* and *CYP2D6* metabolizer groups and treatment arm.

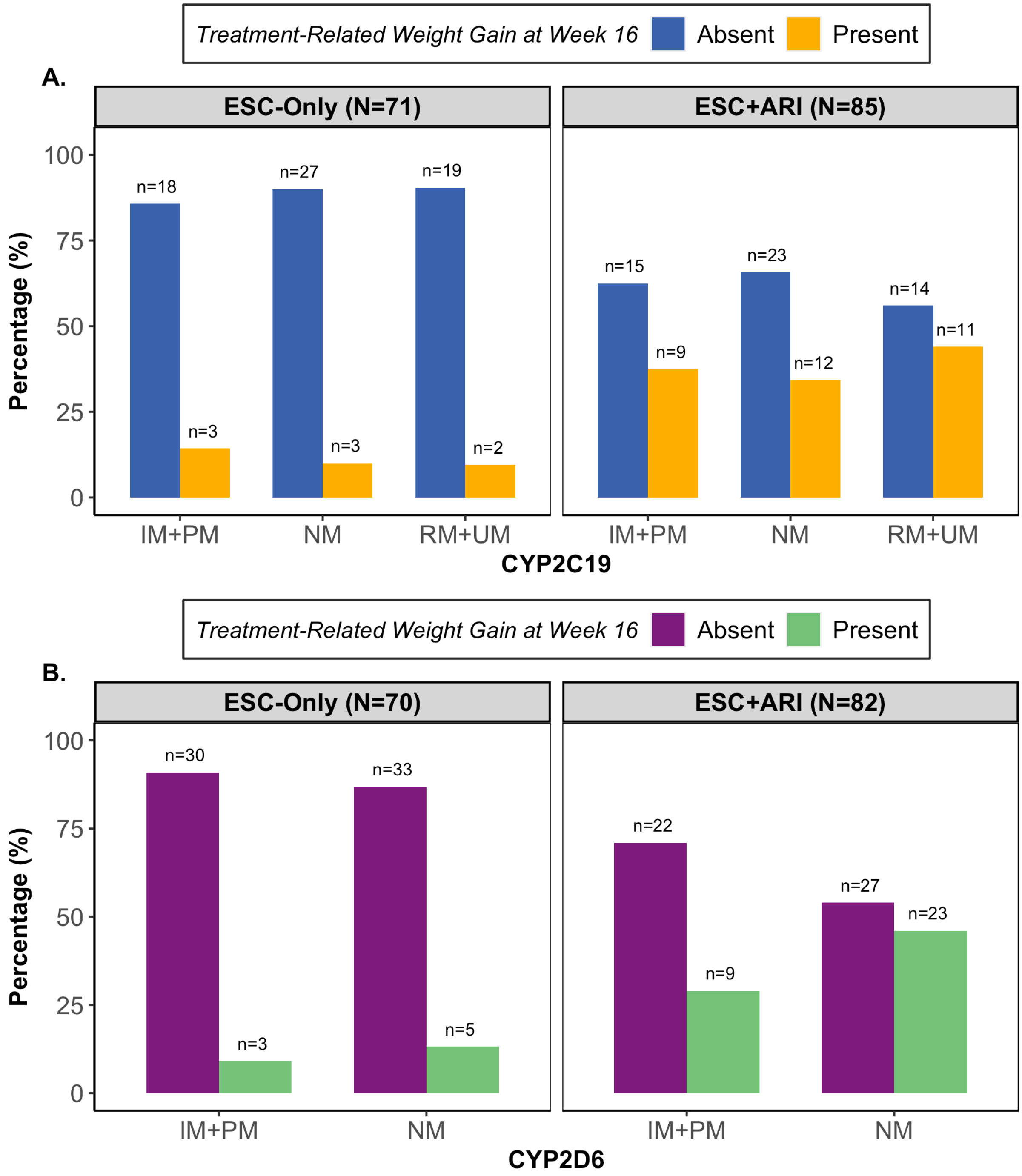

No associations between the treatment-related weight gain at trial end with either *CYP2C19* or *CYP2D6* metabolizer groups were observed.
All logistic regression analyses were adjusted for adjusted for age, ancestry, sex, recruitment site, total MADRS score at baseline, and *CYP2C19* and *CYP2D6* metabolizer groups.
ARI = Aripiprazole; ESC = Escitalopram; IM = Intermediate Metabolizer; NM = Normal Metabolizer; PM = Poor Metabolizer; RM = Rapid Metabolizer; UM = Ultra-rapid Metabolizer.

Figure S10. Intensity of central nervous system (CNS) side effects over time by *CYP2C19* and *CYP2D6* metabolizer group and treatment-arm.

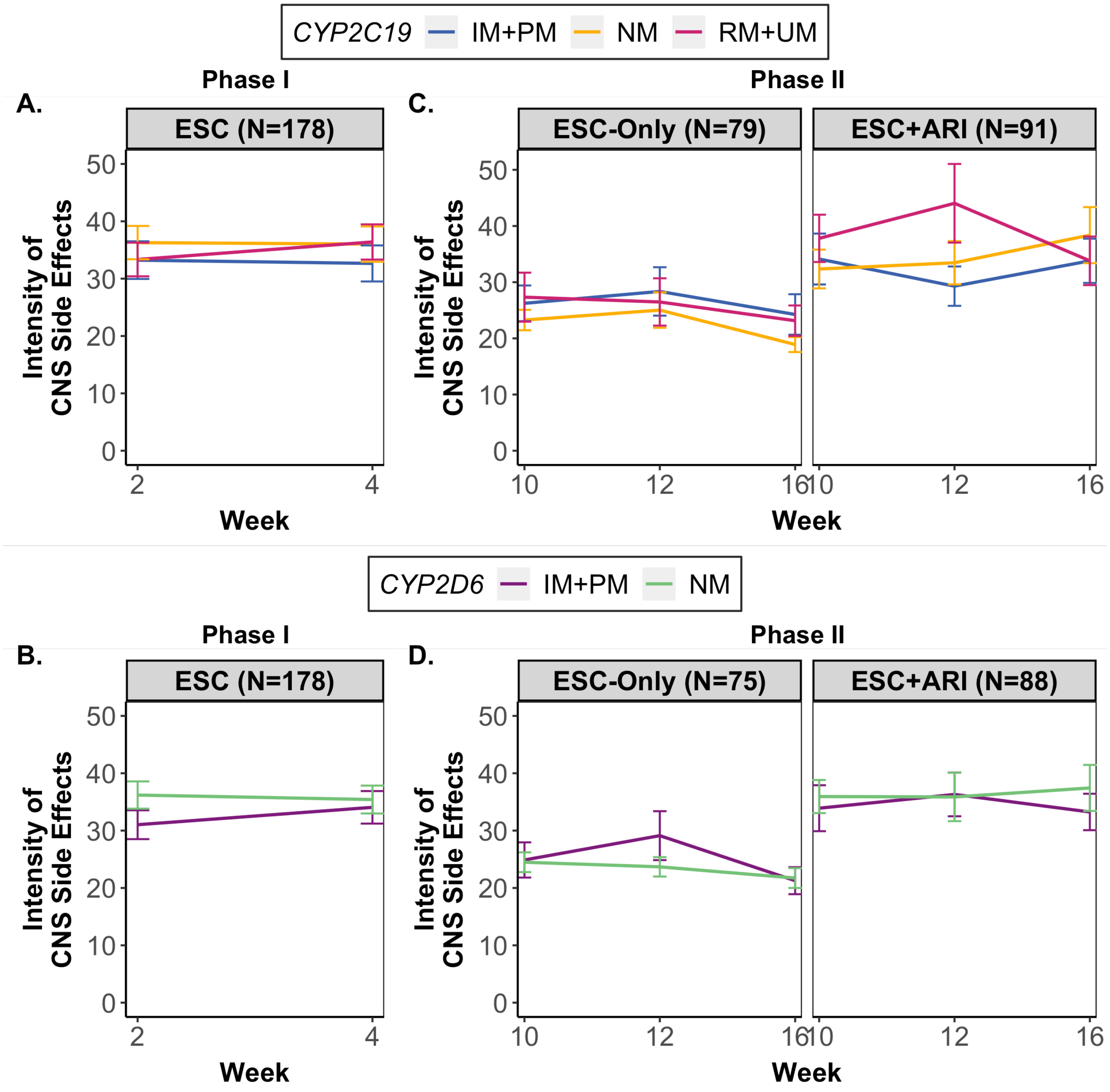

CNS side effect intensity was not associated with *CYP2C19* and *CYP2D6* metabolizer groups during Phases I and II. Error bars represent standard error.
ARI = Aripiprazole; IM = Intermediate Metabolizer; NM = Normal Metabolizer; PM = Poor Metabolizer; RM = Rapid Metabolizer; UM = Ultra-rapid Metabolizer.

Figure S11. Intensity of gastrointestinal side effects over time by *CYP2C19* and *CYP2D6* metabolizer group and treatment-arm.

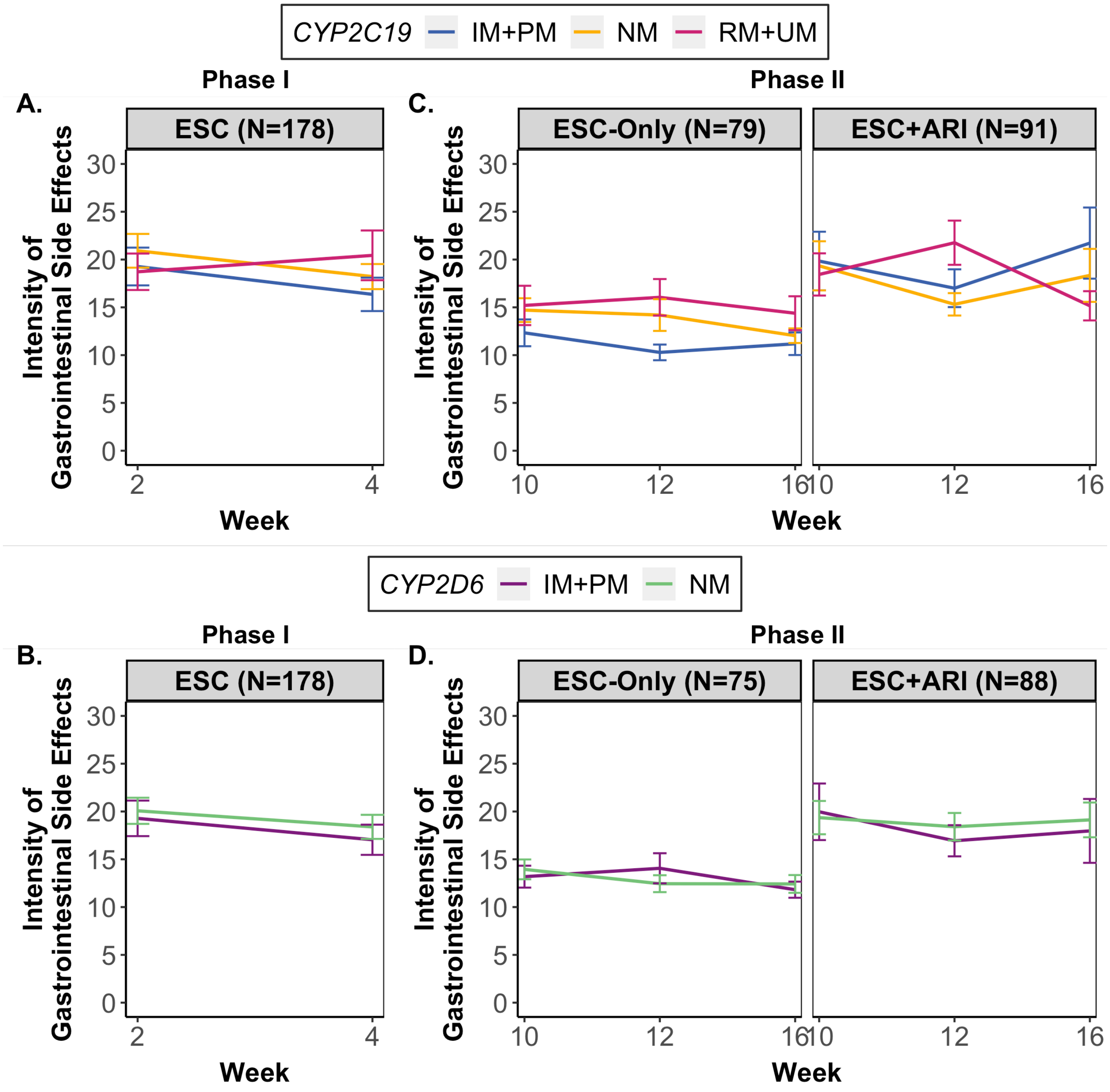

Gastrointestinal side effect intensity was not associated with *CYP2C19* and *CYP2D6* metabolizer groups during Phases I and II. Error bars represent standard error.
ARI = Aripiprazole; IM = Intermediate Metabolizer; NM = Normal Metabolizer; PM = Poor Metabolizer; RM = Rapid Metabolizer; UM = Ultra-rapid Metabolizer.

Figure S12. Intensity of sexual side effects over time by *CYP2C19* and *CYP2D6* metabolizer group and treatment-arm.

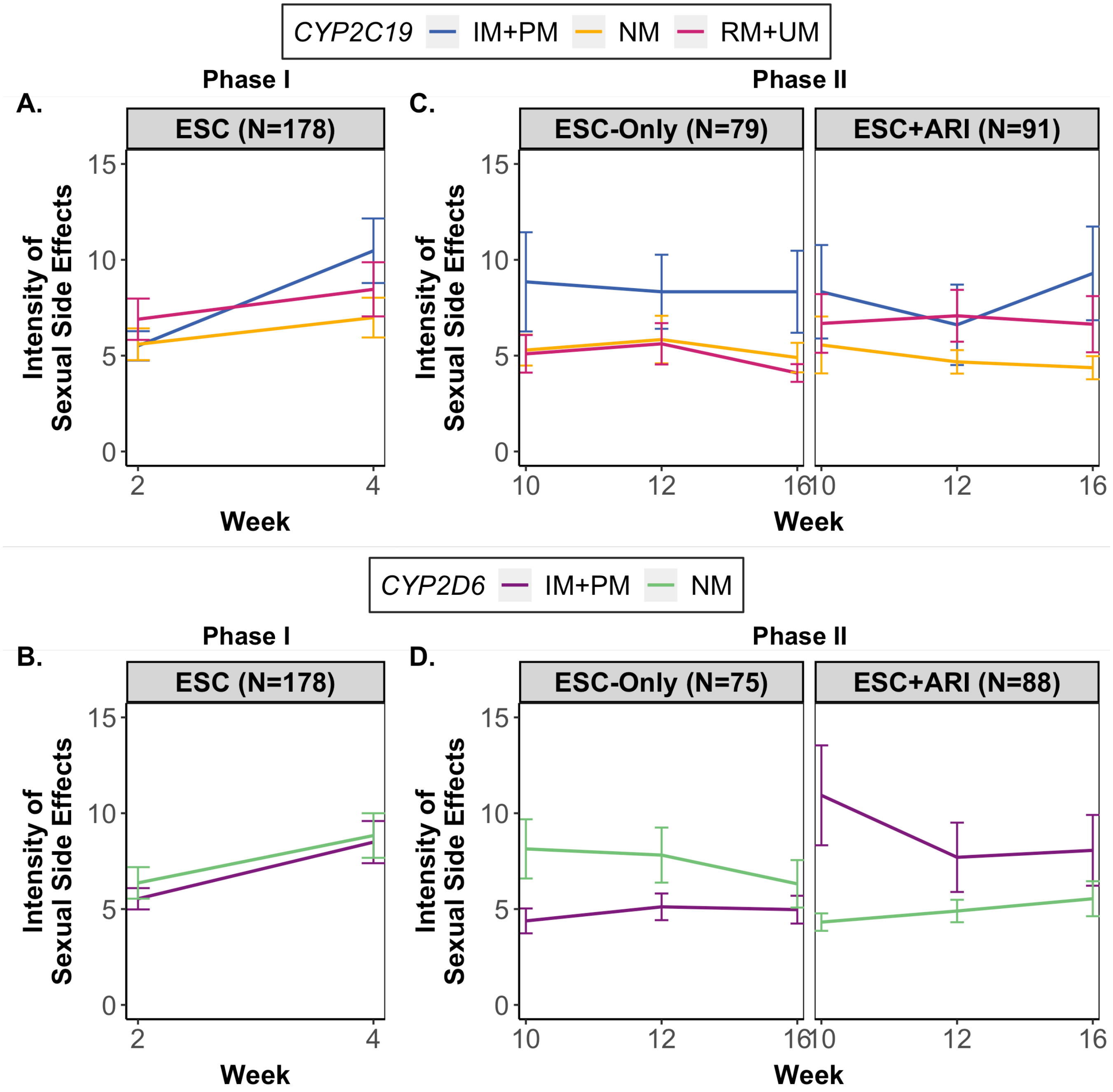

Sexual side effect intensity was not associated with *CYP2C19* and *CYP2D6* metabolizer groups during Phases I and II. Error bars represent standard error.

ARI = Aripiprazole; IM = Intermediate Metabolizer; NM = Normal Metabolizer; PM = Poor Metabolizer; RM = Rapid Metabolizer; UM = Ultra-rapid Metabolizer.

Figure S13. Intensity of treatment-related weight gain over time by *CYP2C19* and *CYP2D6* metabolizer group and treatment-arm.

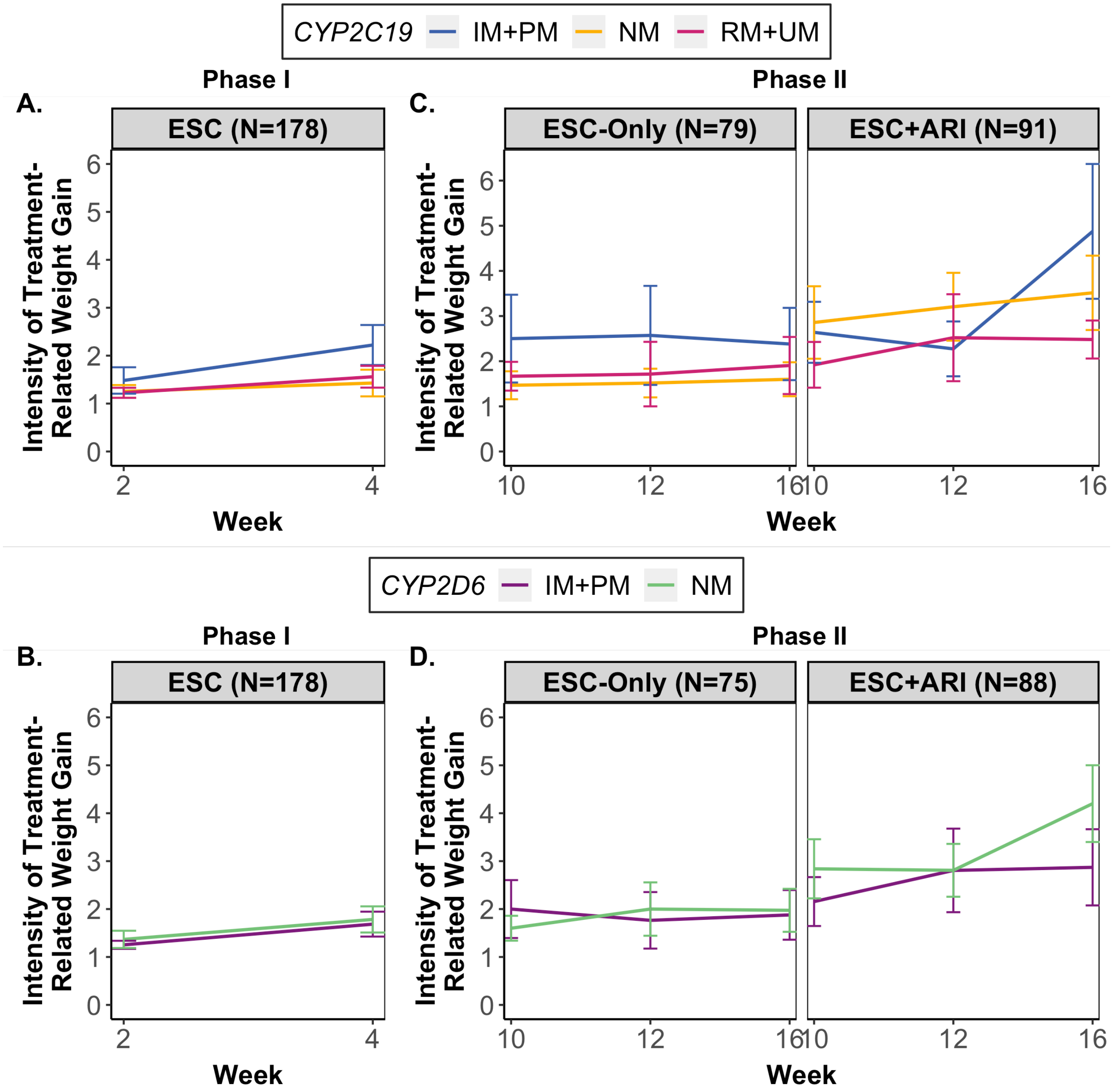

Treatment-related weight gain intensity was not associated with *CYP2C19* and *CYP2D6* metabolizer groups during Phases I and II. Error bars represent standard error.
ARI = Aripiprazole; IM = Intermediate Metabolizer; NM = Normal Metabolizer; PM = Poor Metabolizer; RM = Rapid Metabolizer; UM = Ultra-rapid Metabolizer.

Table S10. Summary of logistic regression models assessing associations of the presence or absence of subcategories of central nervous system side effects with CYP2D6 metabolizer groups for the ESC-Only treatment arm, adjusted for age, ancestry, sex, recruitment site, and total MADRS score at baseline.

			Phase II: ESC-Only		
**Fixed Effects**	**Odds Ratios**	**SE**	**CI (95%)**	**P-Value**	**Summary**
			**Agitation**		
**CYP2D6 IM+PM**	2.38×10^{3}	5.00×10^{4}	$0.00 – 1.93 \times 10^{21}$	0.712	N=71 R^2=0.302 AIC=38.798
			**Blurred Vision**		
**CYP2D6 IM+PM**	4.28	9.32	0.06 – 305.79	0.504	N=71 R^2=0.509 AIC=35.534
			**Decreased Sleepiness**		
**CYP2D6 IM+PM**	0.04 *	0.05	0.00 – 0.50	**0.013 ***	N=71 R^2=0.600 AIC=61.002
			**Drowsiness**		
**CYP2D6 IM+PM**	0.97	0.59	0.29 – 3.22	0.961	N=71 R^2=0.212 AIC=95.386
			**Dizziness**		
**CYP2D6 IM+PM**	0.00	0.00	$0.00 – 1.03 \times 10^{47}$	0.855	N=70 R^2=0.460 AIC=28.456
			**Flushing**		
**CYP2D6 IM+PM**	0.68	1.03	0.04 – 13.13	0.799	N=71 R^2=0.261 AIC=29.898
			**Headache**		
**CYP2D6 IM+PM**	0.26	0.28	0.03 – 2.16	0.213	N=71 R^2=0.405 AIC=65.612
			**Increased Sleepiness**		
**CYP2D6 IM+PM**	0.62	0.66	0.08 – 4.98	0.653	N=71 R^2=0.551 AIC=57.711
			**Nervousness**		

**CYP2D6 IM+PM**	5.34	6.63	0.47 – 60.81	0.177	N=71 R^2=0.327 AIC=42.244
			**Postural Hypotension**		
**CYP2D6 IM+PM**	6.55	8.98	0.45 – 96.20	0.170	N=71 R^2=0.510 AIC=46.021
			**Sweating**		
**CYP2D6 IM+PM**	0.03	0.05	0.00 – 0.79	**0.035 ***	N=71 R^2=0.515 AIC=54.569
			**Tremor**		
**CYP2D6 IM+PM**	1.99	1.83×10^{2}	$0.00 – 1.25 \times 10^{79}$	0.994	N=71 R^2=1.00 AIC=26.002
			**Twitching myoclonus**		
**CYP2D6 IM+PM**	0.00	0.04	$0.00 – 6.36 \times 10^{142}$	0.955	N=71 R^2=1.00 AIC=26.003
			**Weakness and Fatigue**		
**CYP2D6 IM+PM**	0.26	0.24	0.04 – 1.61	0.147	N=71 R^2=0.275 AIC=71.935

AIC = Akaike information criterion; CI = confidence interval; ESC = Escitalopram; IM = Intermediate Metabolizer; NM = Normal Metabolizer; PM = Poor Metabolizer; RM = Rapid Metabolizer; SE = standard error; UM = Ultra-rapid Metabolizer.
*p<0.05

Table S11. Summary of logistic regression models assessing associations of the presence or absence of subcategories of sexual side effects with *CYP2D6* metabolizer groups for the ESC-ARI treatment arm, adjusted for adjusted for age, ancestry, sex, recruitment site, and total MADRS score at baseline.

			Phase II: ESC-Only		
**Fixed Effects**	**Odds Ratios**	**SE**	**CI (95%)**	**P-Value**	**Summary**
			**Anorgasmia**		
**CYP2D6 IM+PM**	3.33	2.54	0.75 – 14.87	0.114	N=81<br>R^2=0.203<br>AIC=84.015
			**Decreased Libido**		
**CYP2D6 IM+PM**	9.63 **	7.79	1.97 – 47.04	**0.005 ****	N=82<br>R^2=0.370<br>AIC=93.730
			**Increased Libido**		
**CYP2D6 IM+PM**	0.00	0.00	0.00 – 4.48×10^3	0.321	N=76<br>R^2=0.745<br>AIC=31.278

AIC = Akaike information criterion; CI = confidence interval; ESC = Escitalopram; IM = Intermediate Metabolizer; NM = Normal Metabolizer; PM = Poor Metabolizer; RM = Rapid Metabolizer; SE = standard error; UM = Ultra-rapid Metabolizer.
*p<0.05

Table S12. Summary of linear mixed effects models assessing association between the intensity of central nervous system, gastrointestinal, and sexual side effects and treatment-related weight gain with every two-week assessment and *CYP2C19* and *CYP2D6* metabolizer groups over the course of Phase I and II, adjusted for age, ancestry, sex, and interaction between time and *CYP2C19* or *CYP2D6* as fixed effects, and site and subject as random effects variables.

	Phase I					*Phase II: ESC-Only*					*Phase II: ESC+ARI*				
**Fixed Effects**	**Estimates**	**SE**	**CI (95%)**	**P-Value**	**df**	**Estimates**	**SE**	**CI (95%)**	**P-Value**	**df**	**Estimates**	**SE**	**CI (95%)**	**P-Value**	**df**
**Central Nervous System (CNS) Side Effects**															
**Week**	-0.03	0.04	-0.10 – 0.04	0.398	164	-0.03	0.01	-0.05 – -0.01	**0.013 ***	136	0.01	0.02	-0.02 – 0.05	0.410	156
**CYP2C19 IM+PM**	-0.11	0.17	-0.44 – 0.22	0.516	157	-0.17	0.25	-0.68 – 0.34	0.516	59	0.01	0.32	-0.62 – 0.64	0.984	69
**CYP2C19 RM+UM**	-0.22	0.17	-0.56 – 0.12	0.209	157	-0.21	0.24	-0.70 – 0.28	0.394	59	0.60	0.32	-0.03 – 1.24	0.063	69
**CYP2D6 IM+PM**	-0.17	0.14	-0.45 – 0.11	0.232	157	0.05	0.20	-0.36 – 0.46	0.811	136	0.16	0.27	-0.38 – 0.70	0.560	156
**CYP2C19 IM+PM*Week**	0.00	0.05	-0.09 – 0.10	0.919	164	0.02	0.02	-0.01 – 0.06	0.199	59	-0.01	0.02	-0.05 – 0.04	0.782	69
**CYP2C19 RM+UM*Week**	0.04	0.05	-0.05 – 0.14	0.373	164	0.02	0.02	-0.01 – 0.05	0.225	59	-0.05	0.02	-0.09 – -0.00	**0.046 ***	69
**CYP2D6 IM+PM*Week**	0.05	0.04	-0.03 – 0.13	0.207	164	-0.01	0.01	-0.04 – 0.02	0.555	136	-0.00	0.02	-0.04 – 0.04	0.944	156
**Model diagnostics**	Observations = 338<br>Marginal R^2 = 0.103<br>AIC = 570.346					Observations = 212<br>Marginal R^2 = 0.140<br>AIC =248.271					Observations = 242<br>Marginal R^2 = 0.142<br>AIC =409.910				
**Gastrointestinal Side Effects**															
**Week**	-0.04	0.04	-0.11 – 0.03	0.232	163	-0.43	0.19	-0.82 – -0.05	**0.029 ***	137	-0.00	0.02	-0.03 – 0.03	0.991	159

**CYP2C19 IM+PM**	-0.02	0.17	-0.35 – 0.31	0.891	157	-8.83	3.86	-16.55 – -1.10	**0.026 ***	59	-0.33	0.32	-0.98 – 0.31	0.307	69
**CYP2C19 RM+UM**	-0.25	0.17	-0.59 – 0.08	0.138	157	-5.23	3.81	-12.86 – 2.41	0.176	59	0.34	0.33	-0.32 – 0.99	0.304	69
**CYP2D6 IM+PM**	0.01	0.14	-0.27 – 0.29	0.942	157	1.77	3.15	-4.54 – 8.07	0.577	59	0.33	0.28	-0.23 – 0.89	0.240	69
**CYP2C19 IM+PM*Week**	-0.03	0.05	-0.12 – 0.07	0.553	163	0.44	0.27	-0.09 – 0.98	0.105	137	0.03	0.02	-0.02 – 0.07	0.259	159
**CYP2C19 RM+UM*Week**	0.05	0.05	-0.04 – 0.15	0.268	163	0.43	0.27	-0.12 – 0.97	0.124	137	-0.03	0.02	-0.07 – 0.02	0.265	159
**CYP2D6 IM+PM*Week**	-0.01	0.04	-0.09 – 0.07	0.853	163	-0.13	0.23	-0.58 – 0.32	0.582	137	-0.03	0.02	-0.07 – 0.01	0.192	159
**Model diagnostics**	Observations = 337<br>Marginal R^2 = 0.198<br>AIC = 548.974					Observations = 213<br>Marginal R^2 = 0.171<br>AIC = 1337.590					Observations = 245<br>Marginal R^2 = 0.158<br>AIC = 420.968				
**Sexual Side Effects**															
**Week**	0.06	0.05	-0.04 – 0.16	0.220	158	-0.25	0.19	-0.62 – 0.12	0.184	137	0.02	0.27	-0.51 – 0.55	0.940	155
**CYP2C19 IM+PM**	-0.16	0.25	-0.65 – 0.33	0.523	157	2.88	3.87	-4.87 – 10.63	0.460	59	-3.75	5.02	-13.76 – 6.26	0.457	68
**CYP2C19 RM+UM**	0.13	0.25	-0.36 – 0.62	0.598	157	1.72	3.73	-5.75 – 9.19	0.647	59	0.01	5.01	-9.98 – 10.00	0.999	68
**CYP2D6 IM+PM**	0.01	0.21	-0.40 – 0.42	0.973	157	-7.54	3.11	-13.75 – -1.32	**0.018 ***	59	12.19	4.32	3.58 – 20.80	**0.006 ** **	68
**CYP2C19 IM+PM*Week**	0.12	0.07	-0.02 – 0.26	0.090	158	-0.01	0.26	-0.53 – 0.51	0.976	137	0.49	0.37	-0.25 – 1.22	0.192	155
**CYP2C19 RM+UM*Week**	0.00	0.07	-0.14 – 0.14	0.959	158	-0.15	0.26	-0.67 – 0.37	0.565	137	0.14	0.37	-0.60 – 0.87	0.715	155
**CYP2D6 IM+PM*Week**	0.01	0.06	-0.10 – 0.13	0.804	158	0.37	0.22	-0.06 – 0.80	0.093	137	-0.61	0.32	-1.24 – 0.02	0.057	155

**Model diagnostics**	Observations = 332<br>Marginal R^2 = 0.116<br>AIC = 786.924					Observations = 213<br>Marginal R^2 = 0.308<br>AIC = 1336.450					Observations = 240<br>Marginal R^2 = 0.206<br>AIC = 1645.792				
**Treatment-Related Weight Gain**															
**Week**	0.10	0.19	-0.28 – 0.48	0.605	162	0.01	0.08	-0.15 – 0.18	0.885	136	0.18	0.18	-0.17 – 0.53	0.320	155
**CYP2C19 IM+PM**	-0.48	0.84	-2.13 – 1.17	0.566	157	0.99	1.74	-2.50 – 4.48	0.573	59	-3.77	3.19	-10.13 – 2.58	0.240	69
**CYP2C19 RM+UM**	-0.24	0.86	-1.94 – 1.46	0.782	157	-0.55	1.68	-3.91 – 2.80	0.742	59	-0.87	3.20	-7.26 – 5.52	0.787	69
**CYP2D6 IM+PM**	-0.03	0.71	-1.43 – 1.38	0.971	157	0.53	1.40	-2.26 – 3.33	0.703	59	1.66	2.74	-3.80 – 7.12	0.545	69
**CYP2C19 IM+PM*Week**	0.32	0.26	-0.21 – 0.84	0.234	162	0.02	0.12	-0.21 – 0.25	0.849	136	0.29	0.24	-0.19 – 0.78	0.228	155
**CYP2C19 RM+UM*Week**	0.08	0.27	-0.45 – 0.62	0.764	162	0.06	0.12	-0.17 – 0.29	0.615	136	-0.05	0.24	-0.53 – 0.44	0.848	155
**CYP2D6 IM+PM*Week**	-0.02	0.23	-0.47 – 0.42	0.916	162	-0.06	0.10	-0.25 – 0.14	0.567	136	-0.17	0.21	-0.58 – 0.24	0.421	155
**Model diagnostics**	Observations = 336<br>Marginal R^2 = 0.047<br>AIC = 1473.695					Observations = 212<br>Marginal R^2 = 0.078<br>AIC = 1012.255					Observations = 241<br>Marginal R^2 = 0.120<br>AIC = 1414.287				

AIC = Akaike information criterion; ARI = Aripiprazole; CI = confidence interval; ESC = Escitalopram; IM = Intermediate Metabolizer; NM = Normal Metabolizer; PM = Poor Metabolizer; RM = Rapid Metabolizer; SE = standard error; UM = Ultra-rapid Metabolizer.
*p<0.05, ** p<0.01, *** p<0.001.

Table S13. Summary of logistic regression models assessing associations of the presence or absence of central nervous system, gastrointestinal, and sexual side effects, and treatment-related weight changes with CYP2C19 and CYP2D6 metabolizer groups during Phase I and II for the European subset, adjusted for age, ancestry, site, and sex.

	Phase I					*Phase II: ESC-Only*					*Phase II: ESC+ARI*				
**Fixed Effects**	**Odds Ratios**	**SE**	**CI (95%)**	**P-Value**	**Summary**	**Odds Ratios**	**SE**	**CI (95%)**	**P-Value**	**Summary**	**Odds Ratios**	**SE**	**CI (95%)**	**P-Value**	**Summary**
**Central Nervous System (CNS) Side Effects**															
**CYP2C19 IM+PM**	1.48	0.88	0.46 – 4.74	0.509	N=125 R^2=0.251 AIC=132.489	0.34	0.34	0.05 – 2.40	0.279	N=53 R^2=0.540 AIC=67.615	1.44	1.30	0.25 – 8.39	0.685	N=63 R^2=0.441 AIC=67.346
**CYP2C19 RM+UM**	2.10	1.16	0.71 – 6.23	0.180		1.46	1.29	0.26 – 8.28	0.666		3.92	3.92	0.55 – 27.80	0.171	
**CYP2D6 IM+PM**	0.66	0.31	0.26 – 1.68	0.384		0.13	0.13	0.02 – 0.91	**0.040**		7.62	7.24	1.18 – 49.14	**0.033**	
**Gastrointestinal Side Effects**															
**CYP2C19 IM+PM**	0.84	0.43	0.31 – 2.26	0.724	N=124 R^2=0.148 AIC=162.568	0.09	0.11	0.01 – 1.06	0.056	N=53 R^2=0.341 AIC=78.47 5	1.24	0.92	0.29 – 5.29	0.771	N=63 R^2=0.250 AIC=92.960
**CYP2C19 RM+UM**	1.51	0.74	0.58 – 3.93	0.395		0.47	0.36	0.11 – 2.07	0.321		1.43	1.01	0.36 – 5.70	0.612	
**CYP2D6 IM+PM**	0.90	0.37	0.40 – 2.03	0.791		1.32	0.91	0.34 – 5.12	0.693		0.61	0.39	0.18 – 2.12	0.439	
**Sexual Side Effects**															
**CYP2C19 IM+PM**	1.91	0.93	0.73 – 4.98	0.185	N=125 R^2=0.097 AIC=180.980	1.88	1.73	0.31 – 11.47	0.495	N=53 R^2=0.169 AIC=78.52 8	5.30	4.37	1.06 – 26.65	**0.043 ***	N=63 R^2=0.260 AIC=89.149
**CYP2C19 RM+UM**	1.84	0.81	0.77 – 4.38	0.171		1.31	1.01	0.29 – 5.91	0.729		3.55	2.70	0.80 – 15.75	0.095	
**CYP2D6 IM+PM**	1.98	0.76	0.93 – 4.22	0.077		0.62	0.42	0.17 – 2.35	0.485		2.62	1.69	0.74 – 9.28	0.135	
**Treatment-Related Weight Gain**															
**CYP2C19 IM+PM**	2.56	1.90	0.60 – 10.9 5	0.204	N=125 R^2=0.139	3.89	4.80	0.35 – 43.75	0.271	N=53 R^2=0.373	0.57	0.41	0.14 – 2.36	0.438	N=62 R^2=0.169

**CYP2C19 RM+UM**	1.60	1.17	0.38 – 6.75	0.522	AIC=100.622	0.46	0.60	0.03 – 6.04	0.553	AIC=48.32<br>9	1.00	0.67	0.27 – 3.68	0.998	AIC=98.021
**CYP2D6 IM+PM**	0.81	0.50	0.24 – 2.70	0.728		0.82	0.99	0.08 – 8.72	0.871		0.65	0.39	0.20 – 2.11	0.470	

AIC = Akaike information criterion; ARI = Aripiprazole; CI = confidence interval; ESC = Escitalopram; IM = Intermediate Metabolizer; NM = Normal Metabolizer; PM = Poor Metabolizer; RM = Rapid Metabolizer; SE = standard error; UM = Ultra-rapid Metabolizer.
*p<0.05, ** p<0.01, *** p<0.001.

Table S14. Summary of linear mixed effects models assessing association between the intensity of central nervous system, gastrointestinal, and sexual side effects and treatment-related weight gain with every two-week assessment and CYP2C19 and CYP2D6 metabolizer groups over the course of Phase I and II for the European subset, adjusted for age, ancestry, sex, and interaction between time and CYP2C19 or CYP2D6 as fixed effects, and site and subject as random effects variables.

	Phase I					*Phase II: ESC-Only*					*Phase II: ESC+ARI*				
**Fixed Effects**	**Estimates**	**SE**	**CI (95%)**	**P-Value**	**df**	**Estimates**	**SE**	**CI (95%)**	**P-Value**	**df**	**Estimates**	**SE**	**CI (95%)**	**P-Value**	**df**
**Central Nervous System (CNS) Side Effects**															
**Week**	-0.04	0.04	-0.12 – 0.05	0.398	120	-0.03	0.01	-0.06 – -0.01	**0.018 ***	103	0.01	0.02	-0.03 – 0.05	0.716	119
**CYP2C19 IM+PM**	-0.15	0.22	-0.60 – 0.29	0.493	115	-0.16	0.34	-0.84 – 0.52	0.638	43	-0.06	0.40	-0.85 – 0.73	0.881	52
**CYP2C19 RM+UM**	-0.25	0.20	-0.64 – 0.15	0.218	115	-0.41	0.26	-0.92 – 0.11	0.121	43	0.46	0.38	-0.29 – 1.21	0.228	52
**CYP2D6 IM+PM**	-0.09	0.18	-0.44 – 0.26	0.616	115	0.09	0.23	-0.38 – 0.55	0.711	43	0.30	0.33	-0.37 – 0.97	0.376	52
**CYP2C19 IM+PM*Week**	0.02	0.06	-0.11 – 0.14	0.775	115	0.02	0.02	-0.03 – 0.07	0.371	43	-0.01	0.03	-0.06 – 0.05	0.813	52
**CYP2C19 RM+UM*Week**	0.06	0.06	-0.05 – 0.17	0.289	120	0.03	0.02	-0.00 – 0.07	0.053	103	-0.04	0.03	-0.09 – 0.02	0.194	119
**CYP2D6 IM+PM*Week**	0.01	0.05	-0.09 – 0.11	0.784	120	-0.01	0.02	-0.04 – 0.02	0.536	103	-0.01	0.02	-0.06 – 0.04	0.637	119
**Model diagnostics**	Observations = 250<br>Marginal R^2 = 0.058<br>AIC = 455.247					Observations = 161<br>Marginal R^2 = 0.179<br>AIC = 190.247					Observations = 186<br>Marginal R^2 = 0.084<br>AIC = 338.843				
**Gastrointestinal Side Effects**															
**Week**	-0.07	0.04	-0.15 – 0.01	0.090	120	-0.43	0.22	-0.86 – -0.00	**0.050**	102	-0.00	0.02	-0.04 – 0.04	0.995	121

**CYP2C19 IM+PM**	-0.12	0.21	-0.53 – 0.30	0.582	115	-7.44	5.20	-17.92 – 3.04	0.160	43	-0.36	0.40	-1.17 – 0.45	0.376	52
**CYP2C19 RM+UM**	-0.30	0.19	-0.68 – 0.07	0.109	115	-8.96	3.98	-16.99 – -0.93	**0.030 ***	43	0.27	0.38	-0.50 – 1.03	0.490	52
**CYP2D6 IM+PM**	-0.03	0.17	-0.36 – 0.30	0.871	115	4.12	3.57	-3.08 – 11.32	0.255	43	0.48	0.34	-0.21 – 1.16	0.169	52
**CYP2C19 IM+PM*Week**	0.00	0.06	-0.12 – 0.12	0.995	115	0.25	0.38	-0.51 – 1.00	0.517	43	0.03	0.03	-0.03 – 0.09	0.297	52
**CYP2C19 RM+UM*Wee k**	0.08	0.05	-0.03 – 0.18	0.147	120	0.60 *	0.29	0.02 – 1.17	**0.042 ***	102	-0.02	0.03	-0.08 – 0.03	0.459	121
**CYP2D6 IM+PM*Week**	-0.02	0.05	-0.11 – 0.08	0.714	120	-0.24	0.26	-0.75 – 0.28	0.370	102	-0.03	0.03	-0.08 – 0.02	0.189	121
**Model diagnostics**	Observations = 250<br>Marginal R^2 = 0.126<br>AIC = 424.021					Observations = 160<br>Marginal R^2 = 0.220<br>AIC = 998.795					Observations = 188<br>Marginal R^2 = 0.153<br>AIC = 339.012				
							**Sexual Side Effects**								
**Week**	0.04	0.06	-0.08 – 0.16	0.546	117	-0.17	0.18	-0.53 – 0.19	0.351	103	0.13	0.30	-0.47 – 0.72	0.680	121
**CYP2C19 IM+PM**	-0.25	0.33	-0.90 – 0.40	0.448	115	-3.69	4.42	-12.60 – 5.22	0.408	43	-1.99	5.92	-13.86 – 9.88	0.738	52
**CYP2C19 RM+UM**	0.05	0.29	-0.51 – 0.62	0.854	115	0.92	3.36	-5.84 – 7.69	0.785	43	0.74	5.62	-10.53 – 12.01	0.896	52
**CYP2D6 IM+PM**	0.05	0.26	-0.46 – 0.55	0.858	115	-3.12	3.01	-9.20 – 2.96	0.307	43	12.03 *	5.01	1.99 – 22.08	**0.020 ***	52
**CYP2C19 IM+PM*Week**	0.17	0.09	-0.01 – 0.35	0.070	115	0.33	0.32	-0.30 – 0.96	0.301	43	0.43	0.44	-0.43 – 1.29	0.325	52
**CYP2C19 RM+UM*Wee k**	0.02	0.08	-0.14 – 0.18	0.774	117	-0.10	0.24	-0.58 – 0.37	0.672	103	0.10	0.41	-0.72 – 0.92	0.817	121

**CYP2D6 IM+PM*Week**	0.03	0.07	-0.11 – 0.18	0.659	117	0.14	0.22	-0.29 – 0.57	0.518	103	-0.61	0.37	-1.34 – 0.12	0.103	121
**Model diagnostics**	Observations = 247<br>Marginal R^2 = 0.106<br>AIC = 611.202					Observations = 161<br>Marginal R^2 = 0.066<br>AIC = 956.098					Observations = 188<br>Marginal R^2 = 0.239<br>AIC = 1294.973				
**Treatment-Related Weight Gain**															
**Week**	0.16	0.21	-0.25 – 0.56	0.446	120	-0.00	0.10	-0.19 – 0.19	0.993	102	0.16	0.22	-0.27 – 0.60	0.460	117
**CYP2C19 IM+PM**	-1.21	0.99	-3.18 – 0.76	0.226	115	3.24	2.35	-1.50 – 7.97	0.175	43	-4.55	4.16	-12.90 – 3.81	0.280	52
**CYP2C19 RM+UM**	-0.31	0.89	-2.08 – 1.46	0.732	115	-0.83	1.79	-4.43 – 2.77	0.645	43	-0.79	3.92	-8.66 – 7.08	0.841	52
**CYP2D6 IM+PM**	0.30	0.79	-1.26 – 1.87	0.700	115	0.51	1.61	-2.73 – 3.75	0.754	43	0.95	3.52	-6.11 – 8.02	0.787	52
**CYP2C19 IM+PM*Week**	0.50	0.31	-0.12 – 1.12	0.115	115	-0.06	0.17	-0.39 – 0.27	0.717	43	0.33	0.32	-0.30 – 0.96	0.302	52
**CYP2C19 RM+UM*Week**	0.07	0.28	-0.49 – 0.63	0.805	120	0.02	0.13	-0.23 – 0.27	0.881	102	-0.07	0.30	-0.66 – 0.53	0.828	117
**CYP2D6 IM+PM*Week**	-0.12	0.25	-0.61 – 0.38	0.641	120	-0.02	0.11	-0.25 – 0.21	0.860	102	-0.09	0.27	-0.62 – 0.45	0.746	117
**Model diagnostics**	Observations = 250<br>Marginal R^2 = 0.055<br>AIC = 1075.204					Observations = 160<br>Marginal R^2 = 0.302<br>AIC = 764.137					Observations = 184<br>Marginal R^2 = 0.113<br>AIC = 1111.061				

AIC = Akaike information criterion; ARI = Aripiprazole; CI = confidence interval; ESC = Escitalopram; IM = Intermediate Metabolizer; NM = Normal Metabolizer; PM = Poor Metabolizer; RM = Rapid Metabolizer; SE = standard error; UM = Ultra-rapid Metabolizer.
*$p<0.05$, ** $p<0.01$, *** $p<0.001$.

Table S15. Summary of linear regression models assessing associations of dose-adjusted serum concentrations of ESC, S-DCT, and S-DCT/ESC ratio at Weeks 2, 10 and 16 with *CYP2C19* and *CYP2D6* metabolizer groups, adjusted for age, ancestry, sex, and recruitment site.

	Week 2				*Week 10: ESC-Only*				*Week 10: ESC+ARI*				*Week 16: ESC-Only*				*Week 16: ESC+ARI*			
**Fixed Effects**	**B**	**SE**	**CI (95%)**	**P-Value**	**B**	**SE**	**CI (95%)**	**P-Value**	**B**	**SE**	**CI (95%)**	**P-Value**	**B**	**SE**	**CI (95%)**	**P-Value**	**B**	**SE**	**CI (95%)**	**P-Value**
**ESC_{adj} concentrations in serum**																				
**CYP2C19 IM+PM**	0.59	0.14	0.30 – 0.87	**<0.001 *****	0.65	0.26	0.14 – 1.17	**0.014 ***	0.69	0.22	0.24 – 1.13	**0.003 ****	0.66	0.26	0.13 – 1.19	**0.016 ***	0.77	0.20	0.37 – 1.17	**<0.001 *****
**CYP2C19 RM+UM**	-0.05	0.14	-0.33 – 0.23	0.703	0.14	0.23	-0.33 – 0.60	0.559	0.01	0.23	-0.45 – 0.47	0.980	-0.10	0.23	-0.57 – 0.37	0.666	0.17	0.21	-0.24 – 0.58	0.413
**CYP2D6 IM+PM**	0.31	0.12	0.07 – 0.54	**0.012 ***	0.71	0.19	0.34 – 1.09	**<0.001 *****	-0.37	0.20	-0.77 – 0.03	0.071	0.75	0.20	0.35 – 1.15	**<0.001 *****	-0.32	0.18	-0.67 – 0.04	0.085
n	160				66				77				67				74			
R^2 / R^2 adj	0.331 / 0.276				0.478 / 0.360				0.284 / 0.150				0.518 / 0.411				0.447 / 0.338			
AIC	367.461				158.916				195.447				168.506				169.115			
**$S\text{-}DCT_{adj}$ concentrations in serum**																				
**CYP2C19 IM+PM**	-0.04	0.05	-0.13 – 0.05	0.397	-0.12	0.12	-0.35 – 0.11	0.309	-0.02	0.09	-0.20 – 0.15	0.784	-0.09	0.11	-0.31 – 0.13	0.412	-0.12	0.09	-0.31 – 0.07	0.201
**CYP2C19 RM+UM**	0.04	0.05	-0.05 – 0.14	0.343	0.02	0.11	-0.20 – 0.23	0.878	0.14	0.09	-0.04 – 0.32	0.129	0.07	0.10	-0.14 – 0.27	0.517	0.08	0.10	-0.11 – 0.28	0.389
**CYP2D6 IM+PM**	-0.08	0.04	-0.16 – 0.00	0.051	-0.11	0.09	-0.28 – 0.06	0.190	-0.11	0.08	-0.27 – 0.05	0.177	-0.12	0.08	-0.29 – 0.05	0.163	-0.12	0.09	-0.29 – 0.05	0.175
n	157				65				77				66				73			
R^2 / R^2 adj	0.174 / 0.105				0.299 / 0.138				0.246 / 0.105				0.251 / 0.082				0.220 / 0.064			
AIC	8.319				53.353				53.017				50.952				56.097			
**$S\text{-}DCT_{adj}/ESC_{adj}$ ratio in serum**																				

**CYP2C19 IM+PM**	-0.13	0.04	-0.20 – -0.05	**0.001 *****	-0.19	0.07	-0.34 – -0.05	**0.008 ****	-0.17	0.06	-0.29 – -0.04	**0.008 ****	-0.16	0.06	-0.28 – -0.05	**0.007 ****	-0.22	0.06	-0.34 – -0.10	**<0.001 *****
**CYP2C19 RM+UM**	0.03	0.04	-0.05 – 0.10	0.462	-0.03	0.06	-0.16 – 0.10	0.654	0.05	0.06	-0.07 – 0.18	0.403	0.02	0.05	-0.09 – 0.12	0.730	-0.05	0.06	-0.17 – 0.07	0.420
**CYP2D6 IM+PM**	-0.11	0.03	-0.17 – -0.05	**0.001 *****	-0.17	0.05	-0.28 – -0.07	**0.002 ****	0.07	0.05	-0.04 – 0.18	0.206	-0.20	0.04	-0.28 – -0.11	**<0.001 *****	-0.00	0.05	-0.11 – 0.10	0.942
n	157				65				76				66				72			
R^2 / R^2 adj	0.373 / 0.321				0.457 / 0.332				0.333 / 0.206				0.542 / 0.438				0.366 / 0.238			
AIC	-59.260				-11.298				-4.780				-36.228				-15.476			

Serum levels are adjusted for dosage.
AIC = Akaike information criterion; ARI = Aripiprazole; CI = confidence interval; ESC = Escitalopram; IM = Intermediate Metabolizer; NM = Normal Metabolizer; PM = Poor Metabolizer; RM = Rapid Metabolizer; SE = standard error; UM = Ultra-rapid Metabolizer.
p*<0.05, ** *p*<0.01, * *p*<0.001.

Figure S14. Serum concentrations of dose-corrected ESC by ungrouped *CYP2C19* and *CYP2D6* metabolizer phenotypes and treatment-arm.

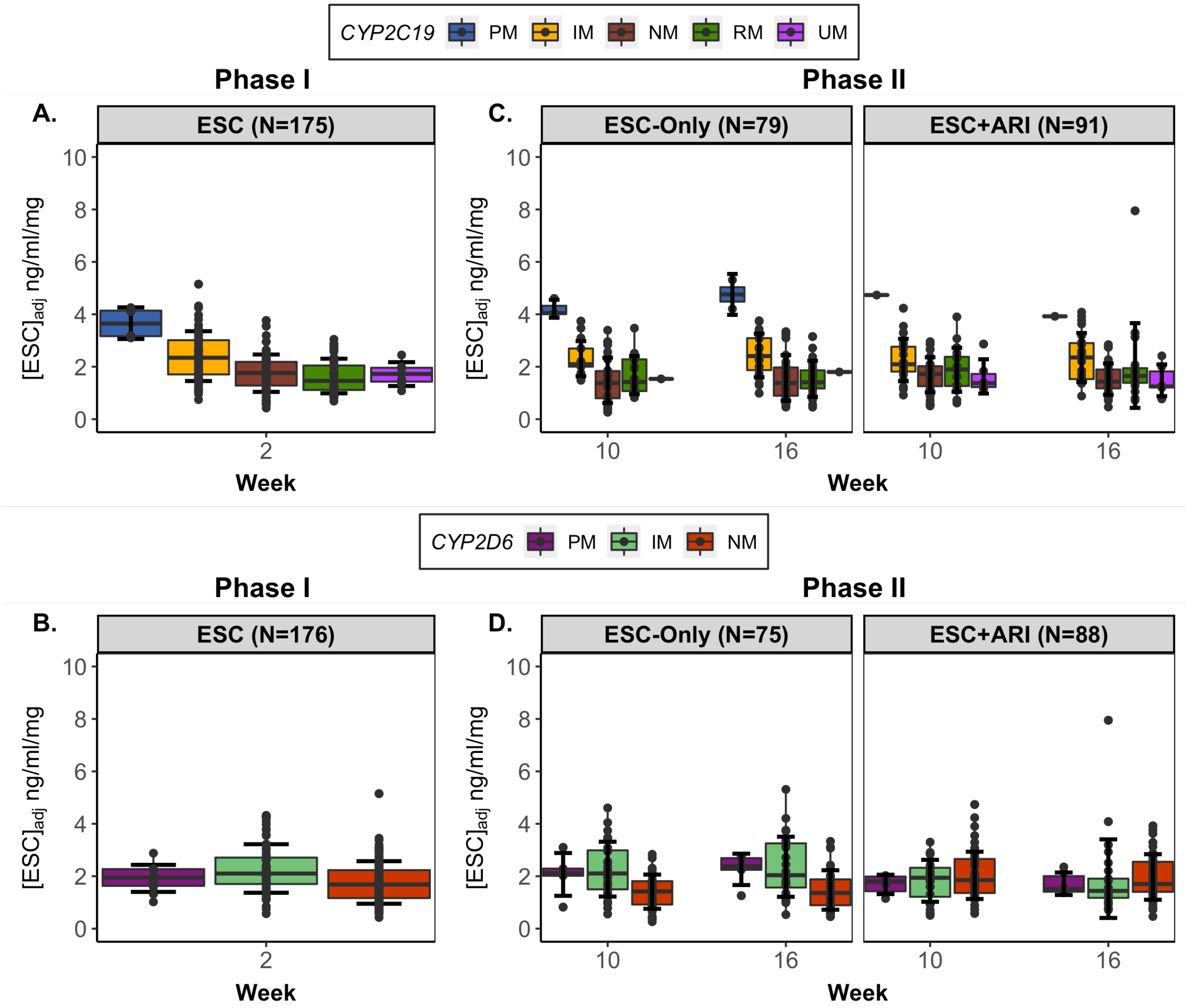

Descriptive plots of ESC_{adj} concentrations at Weeks 2, 10 and 16 by ungrouped *CYP2C19* and *CYP2D6* metabolizer phenotypes during Phases I and II. Error bars represent standard error.
ARI = Aripiprazole; ESC_{adj} = Dose-Adjusted Escitalopram; IM = Intermediate Metabolizer; NM = Normal Metabolizer; PM = Poor Metabolizer; RM = Rapid Metabolizer; UM = Ultra-rapid Metabolizer.

Table S16. Summary of linear regression models assessing associations of dose-adjusted serum concentrations of ESC, S-DCT, and S-DCT/ESC ratio at Weeks 2, 10 and 16 with CYP2C19 and CYP2D6 metabolizer groups for the European subset, adjusted for age, ancestry, sex, and recruitment site.

	Week 2				*Week 10: ESC-Only*				*Week 10: ESC+ARI*				*Week 16: ESC-Only*				*Week 16: ESC+ARI*			
**Fixed Effects**	**B**	**SE**	**CI (95%)**	**P-Value**	**B**	**SE**	**CI (95%)**	**P-Value**	**B**	**SE**	**CI (95%)**	**P-Value**	**B**	**SE**	**CI (95%)**	**P-Value**	**B**	**SE**	**CI (95%)**	**P-Value**
**ESC_{adj} concentrations in serum**																				
**CYP2C19 IM+PM**	0.50	0.17	0.17 – 0.83	**0.003 ****	0.62	0.30	0.00 – 1.23	**0.050**	0.60	0.26	0.06 – 1.13	**0.029 ***	0.56	0.29	-0.03 – 1.14	0.060	0.67	0.25	0.16 – 1.18	**0.011 ***
**CYP2C19 RM+UM**	-0.07	0.15	-0.36 – 0.23	0.654	0.20	0.24	-0.29 – 0.70	0.412	0.05	0.24	-0.44 – 0.55	0.831	-0.02	0.22	-0.47 – 0.42	0.921	0.17	0.25	-0.33 – 0.67	0.497
**CYP2D6 IM+PM**	0.35	0.13	0.09 – 0.61	**0.010 ****	0.72	0.21	0.30 – 1.14	**0.001 ****	-0.29	0.23	-0.75 – 0.17	0.206	0.96	0.20	0.56 – 1.37	**<0.001 *****	-0.17	0.22	-0.62 – 0.27	0.436
n	122				52				58				50				56			
R^2 / R^2 adj	0.307 / 0.238				0.438 / 0.283				0.345 / 0.189				0.552 / 0.423				0.344 / 0.180			
AIC	273.737				125.626				144.392				113.995				137.884			
**$S\text{-}DCT_{adj}$ concentrations in serum**																				
**CYP2C19 IM+PM**	-0.06	0.06	-0.18 – 0.05	0.280	-0.16	0.13	-0.42 – 0.10	0.218	-0.07	0.09	-0.25 – 0.12	0.454	-0.08	0.11	-0.31 – 0.14	0.456	-0.22	0.09	-0.40 – -0.04	**0.019 ***
**CYP2C19 RM+UM**	0.01	0.05	-0.09 – 0.11	0.813	-0.01	0.10	-0.22 – 0.20	0.891	0.08	0.08	-0.09 – 0.25	0.346	0.03	0.09	-0.14 – 0.21	0.717	-0.00	0.09	-0.18 – 0.17	0.972
**CYP2D6 IM+PM**	-0.08	0.05	-0.17 – 0.01	0.081	-0.07	0.09	-0.24 – 0.11	0.440	-0.13	0.08	-0.29 – 0.03	0.102	-0.05	0.08	-0.21 – 0.11	0.559	-0.14	0.08	-0.29 – 0.02	0.091
n	120				51				59				49				56			
R^2 / R^2 adj	0.213 / 0.133				0.421 / 0.257				0.376 / 0.230				0.457 / 0.295				0.419 / 0.274			
AIC	10.908				32.407				22.218				18.612				20.957			

S-DCT_{adj}/ESC_{adj} ratio in serum																				
**CYP2C19 IM+PM**	-0.13	0.05	-0.23 – -0.04	**0.005 ****	-0.23	0.09	-0.42 – -0.04	**0.021 ***	-0.19	0.07	-0.34 – -0.05	**0.011 ***	-0.17	0.08	-0.33 – -0.01	**0.043 ***	-0.25	0.07	-0.38 – -0.12	**<0.001 *****
**CYP2C19 RM+UM**	0.02	0.04	-0.06 – 0.10	0.640	-0.06	0.08	-0.22 – 0.09	0.401	0.04	0.07	-0.10 – 0.17	0.608	-0.02	0.06	-0.14 – 0.11	0.787	-0.08	0.06	-0.20 – 0.05	0.245
**CYP2D6 IM+PM**	-0.13	0.04	-0.20 – -0.05	**0.001 *****	-0.19	0.06	-0.31 – -0.06	**0.005 ****	0.09	0.06	-0.04 – 0.22	0.159	-0.24	0.06	-0.35 – -0.12	**<0.001 *****	-0.03	0.06	-0.15 – 0.08	0.551
n	120				51				58				49				55			
R^2 / R^2 adj	0.393 / 0.331				0.440 / 0.282				0.440 / 0.306				0.492 / 0.341				0.426 / 0.279			
AIC	-36.767				0.964				-4.545				-14.594				-13.894			

Serum levels are adjusted for dosage.
AIC = Akaike information criterion; ARI = Aripiprazole; CI = confidence interval; ESC = Escitalopram; IM = Intermediate Metabolizer; NM = Normal Metabolizer; PM = Poor Metabolizer; RM = Rapid Metabolizer; SE = standard error; UM = Ultra-rapid Metabolizer.
p*<0.05, ** *p*<0.01, * *p*<0.001.

Figure S15. Serum concentrations of dose-corrected ESC by *CYP2C19* and *CYP2D6* treatment arm and metabolizer groups during Phase II.

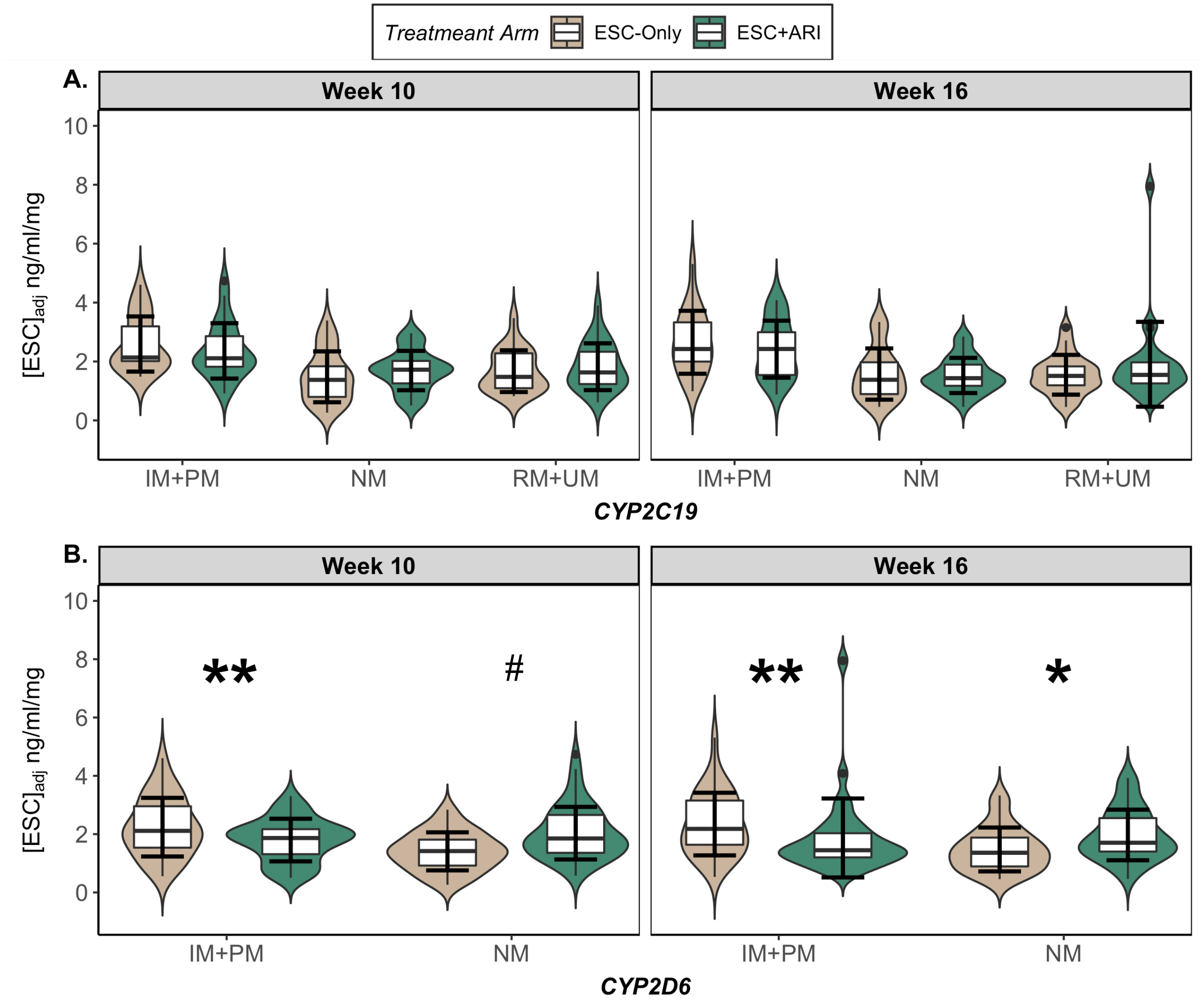

(A) *CYP2C19* NMs, IM+PMs, and RM+UMs did not differ in ESC_{adj} concentrations by treatment arm, whereas **(B)** *CYP2D6* NMs in ESC+ARI demonstrated significantly higher ESC_{adj} serum levels compared to *CYP2D6* NMs in ESC-Only at both Weeks 10 (W=381, p=0.050, r=-0.52) and 16 (W=549, p=0.006, r=-0.47). *CYP2D6* IM+PMs in ESC+ARI had lower ESC levels than CYP2D6 IM+PMs in ESC-Only at Week 16 (W=689, p=0.017, r=-0.56). Error bars represent standard error.
ARI = Aripiprazole; ESC_{adj} = Dose-Adjusted Escitalopram; IM = Intermediate Metabolizer; NM = Normal Metabolizer; PM = Poor Metabolizer; RM = Rapid Metabolizer; UM = Ultra-rapid Metabolizer.
**p<0.01; *p<0.05; # indicates trend with p between 0.050 and 0.070.

Figure S16. Serum concentrations of dose-corrected S-DCT by *CYP2C19* and *CYP2D6* metabolizer groups and treatment-arm.

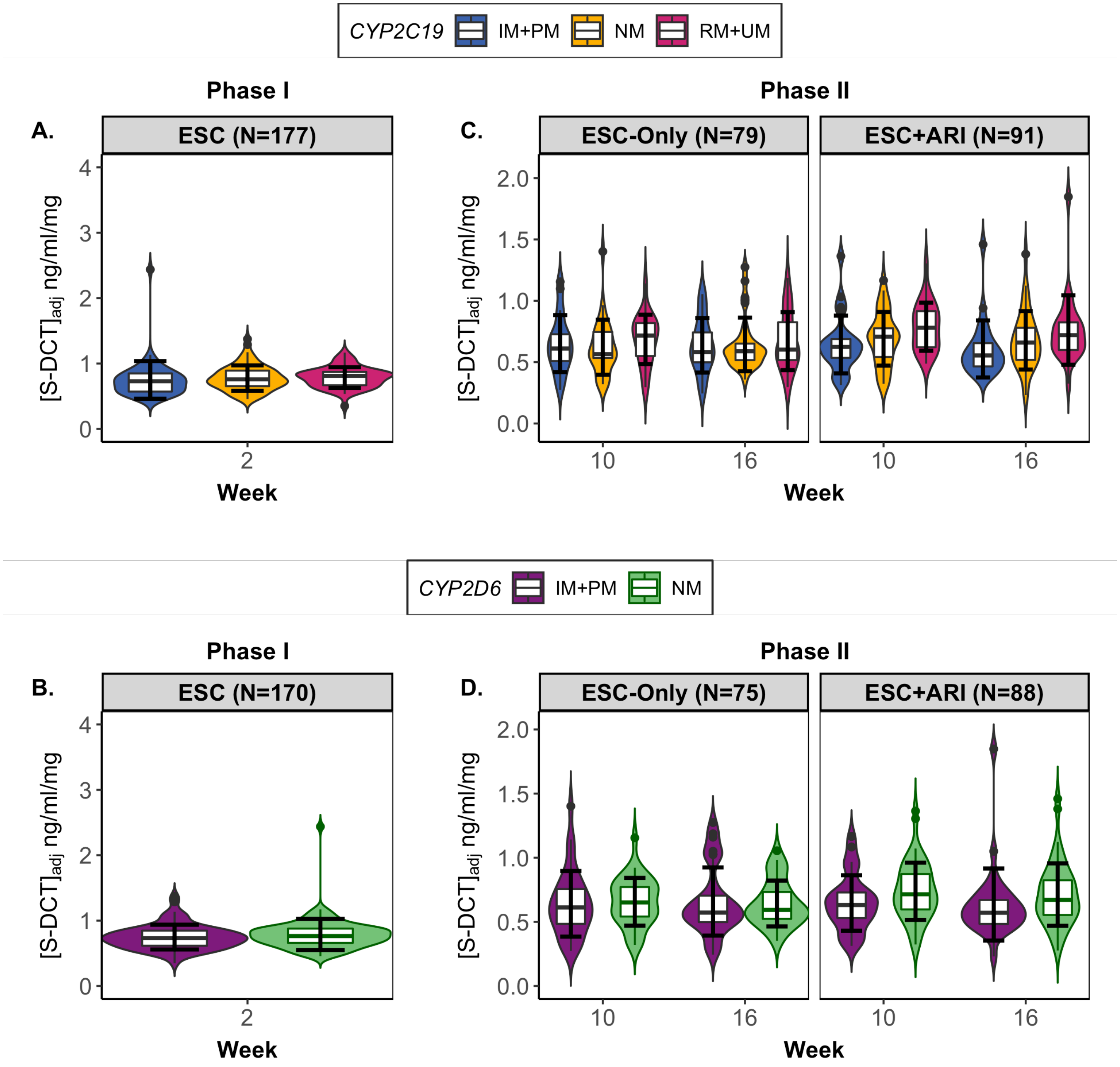

S-DCT$_{ad}$ was not associated with either *CYP2C19* or *CYP2D6* metabolizer groups during (**A-B**) Phase I or (**C-D**) Phase II. All linear regression analyses were adjusted for age, ancestry, sex, site, time since last dose, *CYP2C19* or *CYP2D6* metabolizer groups. Error bars represent standard error. *P*-values are corrected for multiple testing using the false discovery rate (FDR) approach.

ARI = Aripiprazole; ESC = Escitalopram; NM = normal metabolizer, PM = Poor Metabolizer; RM = Rapid Metabolizer; SE = standard error; S-DCT = S-desmethylcitalopram; UM = Ultra-rapid Metabolizer.

* q<0.05, ** q<0.01, *** q<0.001.

Figure S17. Dose-adjusted serum S-DCT$_{adj}$/ESC$_{adj}$ ratio for Phase I and II by ungrouped *CYP2C19* and *CYP2D6* metabolizer phenotypes.

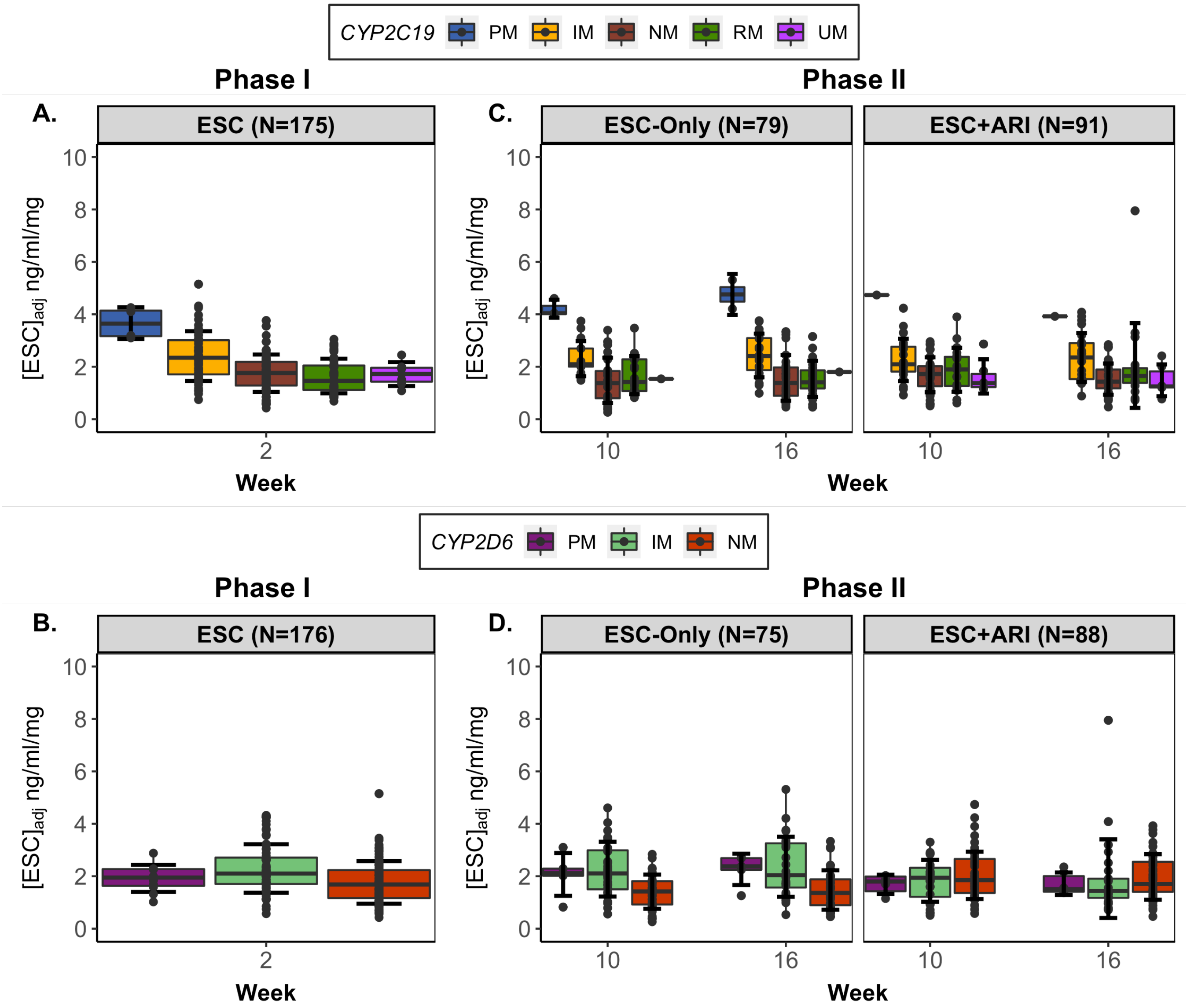

Descriptive plots of S-DCT$_{adj}$/ESC$_{adj}$ concentrations at Weeks 2, 10 and 16 by ungrouped *CYP2C19* and *CYP2D6* metabolizer phenotypes during Phases I and II. Error bars represent standard error.
ARI = Aripiprazole; ESC$_{adj}$ = Dose-Adjusted Escitalopram; IM = Intermediate Metabolizer; NM = Normal Metabolizer; PM = Poor Metabolizer; RM = Rapid Metabolizer; UM = Ultra-rapid Metabolizer.

Table S17. Summary of linear regression models assessing associations of dose-adjusted serum concentrations of ARI, DHA, and DHA/ARI ratio at Weeks 10 and 16 with CYP2D6 metabolizer status, adjusted for age, ancestry, sex, and recruitment site.

	Week 10				*Week 16*			
**Fixed Effects**	**B**	**SE**	**CI (95%)**	**P-Value**	**B**	**SE**	**CI (95%)**	**P-Value**
**ARI concentrations in serum**								
**CYP2D6 IM+PM**	-2.48	1.38	-5.23 – 0.27	0.076	1.54	1.70	-1.86 – 4.95	0.369
Observations	77				74			
R^2 / R^2 adjusted	0.253 / 0.139				0.101 / -0.042			
AIC	490.809				501.372			
**DHA concentrations in serum**								
**CYP2D6 IM+PM**	-0.99 **	0.34	-1.67 – -0.30	**0.006**	-0.47	0.58	-1.63 – 0.69	0.420
Observations	72				72			
R^2 / R^2 adjusted	0.230 / 0.104				0.033 / -0.125			
AIC	249.848				330.658			
**DHA/ARI ratio in serum**								
**CYP2D6 IM+PM**	-0.05 *	0.02	-0.09 – -0.01	**0.028**	-0.07 **	0.03	-0.12 – -0.02	**0.010**
Observations	72				72			
R^2 / R^2 adjusted	0.383 / 0.281				0.289 / 0.172			
AIC	-158.842				-111.987			

Serum levels are adjusted for dosage.
AIC=Akaike information criterion; ARI=Aripiprazole; CI=confidence interval; DHA = dehydroaripiprazole; ESC=Escitalopram; IM = Intermediate Metabolizer; NM = Normal Metabolizer; PM = Poor Metabolizer; SE=standard error.
p*<0.05, ** *p*<0.01, * *p*<0.001.

Figure S18. Serum concentrations of dose-corrected ARI_{adj}, DHA_{adj}, and the DHA_{adj}/ARI_{adj} ratio for the ESC+ARI treatment arm by *CYP2D6* metabolizer group.

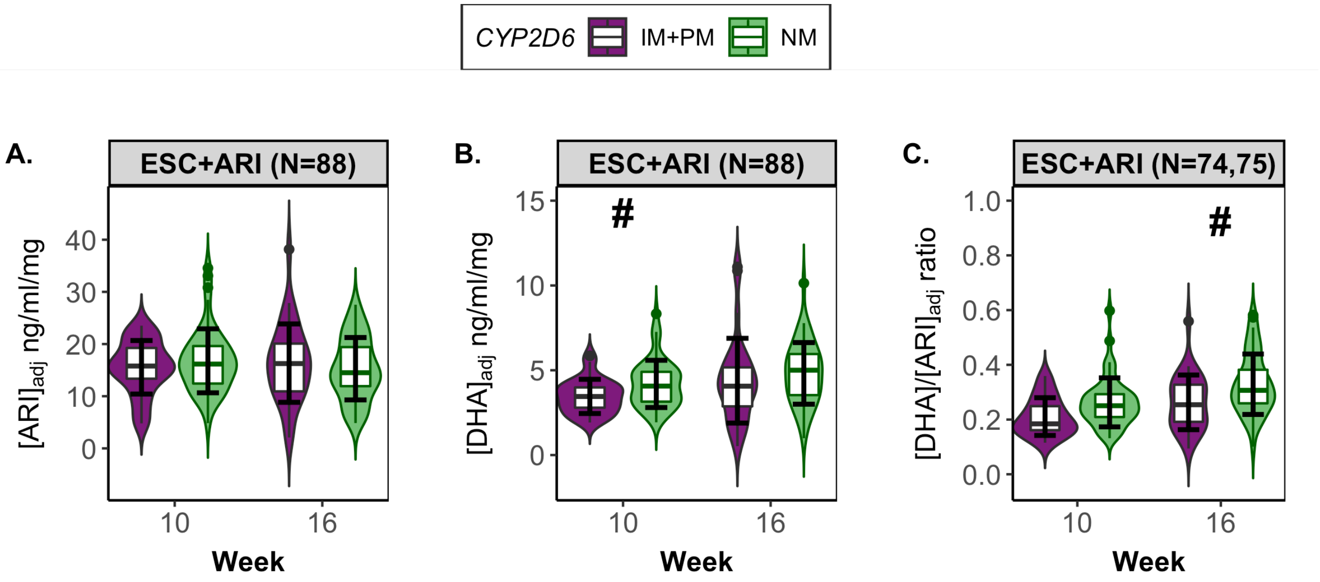

During Phase II, in the ESC+ARI treatment arm, mean (**A**) ARI_{adj} serum concentrations were not associated with *CYP2D6* metabolizer groups. (**B**) A trend for an association was observed between serum DHA_{adj} concentrations and *CYP2D6* metabolizer groups at Week 10 with IM+PMs demonstrating lower DHA_{adj} concentrations relative to NMs. (**C**) A trend for an association was observed between serum DHA/ARI_{adj} ratio and *CYP2D6* metabolizer groups with *CYP2D6* IM+PMs showing lower ARI_{adj}/DHA_{adj} ratio compared to NMs at only Week 16.
All linear regression analyses were adjusted for age, ancestry, sex, site, and time since last dose. Error bars represent standard error.
ARI = Aripiprazole; DHA = dehydroaripiprazole; ESC = Escitalopram; IM = Intermediate Metabolizer; NM = Normal Metabolizer; PM = Poor Metabolizer.
# indicates trend with *q* between 0.050 to 0.070.

Figure S19. Serum concentrations of dose-corrected ARI_{adj}, DHA_{adj}, and the DHA_{adj}/ARI_{adj} ratio for the ESC+ARI treatment arm by ungrouped *CYP2D6* metabolizer group.

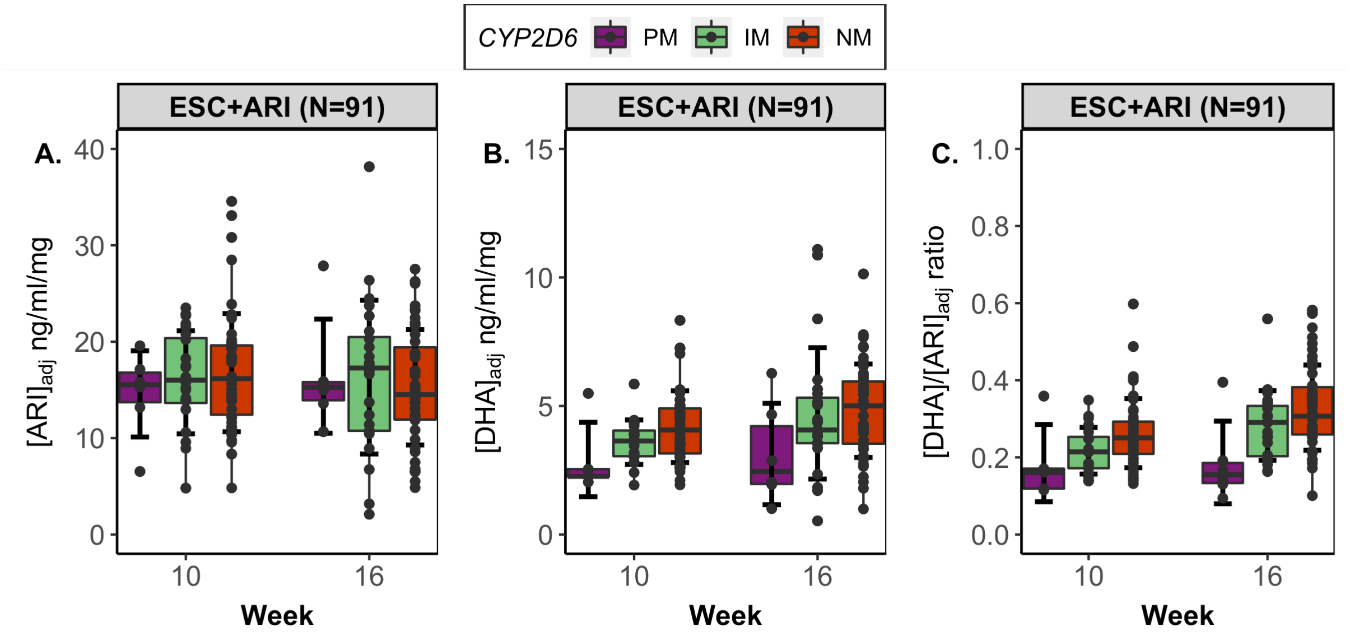

Descriptive plots of ARI_{adj}, DHA_{adj}, and the DHA_{adj}/ARI_{adj} ratio in serum at Weeks 10 and 16 by ungrouped *CYP2D6* metabolizer phenotypes during Phases II. Error bars represent standard error.
ARI = Aripiprazole; ESC_{adj} = Dose-Adjusted Escitalopram; IM = Intermediate Metabolizer; NM = Normal Metabolizer; PM = Poor Metabolizer.

Table S18. Summary of linear regression models assessing associations of dose-adjusted serum concentrations of ARI, DHA, and DHA/ARI ratio at Weeks 10 and 16 with CYP2D6 metabolizer status for the European subset, adjusted for age, ancestry, sex, and recruitment site.

	Week 10				*Week 16*			
**Fixed Effects**	**B**	**SE**	**CI (95%)**	**P-Value**	**B**	**SE**	**CI (95%)**	**P-Value**
**ARI concentrations in serum**								
**CYP2D6 IM+PM**	-3.21	1.60	-6.43 – 0.01	0.051	2.47	1.97	-1.49 – 6.42	0.216
Observations	58				57			
R^2 / R^2 adjusted	0.309 / 0.179				0.145 / -0.019			
AIC	369.596				388.198			
**DHA concentrations in serum**								
**CYP2D6 IM+PM**	-1.27	0.42	-2.13 – -0.42	**0.004 ****	-0.08	0.73	-1.55 – 1.38	0.908
Observations	55				56			
R^2 / R^2 adjusted	0.237 / 0.085				0.044 / -0.143			
AIC	194.887				268.727			
**DHA/ARI ratio in serum**								
**CYP2D6 IM+PM**	-0.05	0.02	-0.10 – -0.01	**0.028 ***	-0.07	0.03	-0.14 – -0.01	**0.022 ***
Observations	55				56			
R^2 / R^2 adjusted	0.374 / 0.249				0.357 / 0.231			
AIC	-129.074				-82.646			

Serum levels are adjusted for dosage.
AIC=Akaike information criterion; ARI=Aripiprazole; CI=confidence interval; DHA = dehydroaripiprazole; ESC=Escitalopram; IM = Intermediate Metabolizer; NM = Normal Metabolizer; PM = Poor Metabolizer; SE=standard error.
*p<0.05, ** p<0.01, *** p<0.001.

Table S19. Spearman correlations of measures of serum concentrations with symptom improvement from baseline and intensity of side effects at Weeks 2, 10 and 16.

	**Week 2 of Phase I**		
	**[ESC_{adj}]**	**[$S\text{-}DCT_{adj}$]**	**[S-DCT]/[ESC_{adj}]**
**%Δ in MADRS from Baseline**	-0.09	-0.19	-0.002
**CNS intensity**	-0.02	0.05	0.05
**Gastrointestinal intensity**	0.03	0.07	0.01
**Sexual intensity**	-0.06	-0.02	0.03
**Weight gain intensity**	0.02	-0.16	-0.08

	**ESC-Only: Week 10 of Phase II**		
	**[ESC_{adj}]**	**[$S\text{-}DCT_{adj}$]**	**[S-DCT]/[ESC_{adj}]**
**%Δ in MADRS from Baseline**	-0.09	-0.11	0.03
**CNS intensity**	0.20	0.23	-0.20
**Gastrointestinal intensity**	-0.13	-0.06	0.11
**Sexual intensity**	**-0.36***	-0.05	**0.41***
**Weight gain intensity**	-0.02	-0.09	-0.04
	**ESC-Only: Week 16 of Phase II**		
	**[ESC_{adj}]**	**[$S\text{-}DCT_{adj}$]**	**[S-DCT]/[ESC_{adj}]**
**%Δ in MADRS from Baseline**	0.16	0.39	0.49
**CNS intensity**	0.77	0.44	0.99
**Gastrointestinal intensity**	0.21	0.38	0.18
**Sexual intensity**	0.42	0.73	0.11
**Weight gain intensity**	0.058	0.019	0.31

	**ESC+ARI: Week 10 of Phase II**					
	**[ESC_{adj}]**	**[$S\text{-}DCT_{adj}$]**	**[S-DCT]/[ESC_{adj}]**	**[ARI_{adj}]**	**[DHA_{adj}]**	**[DHA]/[ARI_{adj}]**
**%Δ in MADRS from Baseline**	-0.1	-0.02	0.12	0.06	-0.09	-0.03
**CNS intensity**	0.07	0.007	-0.02	0.09	-0.15	-0.15
**Gastrointestinal intensity**	0.07	-0.01	-0.05	-0.09	-0.23	-0.13
**Sexual intensity**	-0.03	-0.09	-0.02	0.004	-0.23	-0.23
**Weight gain intensity**	0.3	0.13	-0.17	0.15	-0.03	-0.19
	**ESC+ARI: Week 16 of Phase II**					
	**[ESC_{adj}]**	**[$S\text{-}DCT_{adj}$]**	**[S-DCT]/[ESC_{adj}]**	**[ARI_{adj}]**	**[DHA_{adj}]**	**[DHA]/[ARI_{adj}]**
**%Δ in MADRS from Baseline**	-0.10	0.03	0.09	**-0.36***	**-0.35***	0.26
**CNS intensity**	0.12	0.11	-0.12	0.09	0.11	-0.08
**Gastrointestinal intensity**	0.21	0.26	-0.09	-0.02	-0.04	-0.04
**Sexual intensity**	0.01	0.05	-0.09	-0.02	-0.12	-0.09
**Weight gain intensity**	0.23	0.1	-0.10	0.16	0.14	-0.02

Serum levels are adjusted for dosage.
ARI = Aripiprazole; DHA = dehydroaripiprazole; ESC = Escitalopram; MADRS = Montgomery-Asberg Depression Rating Scale; S-DCT = S-desmethylcitalopram.
$*q<0.05$

Figure S20. Spearman correlation between ESC metabolite-to-drug ratio and sexual side effect intensity at Week 10.

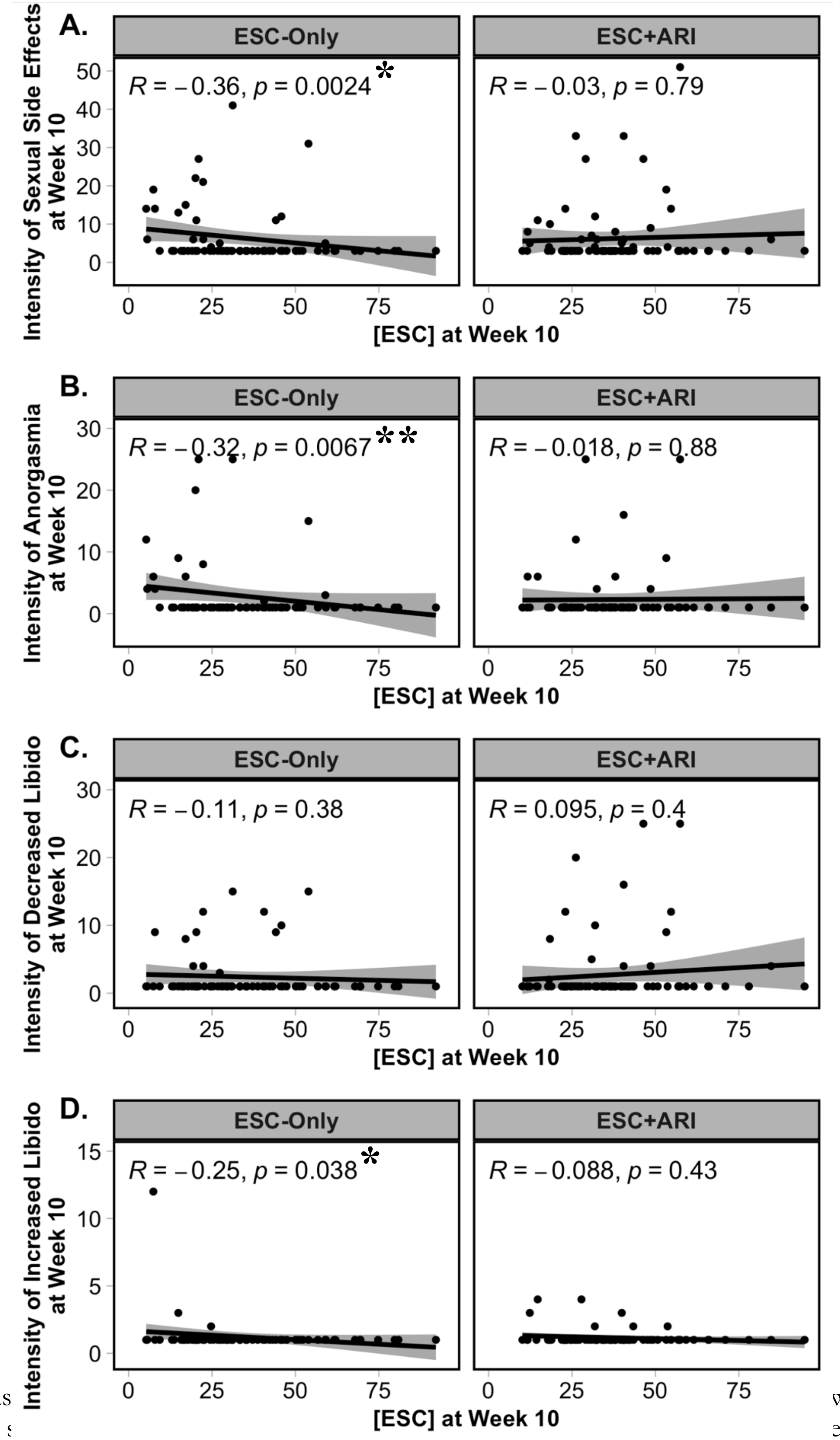

(A) During Phas [illegible] was significantly correlated with s [illegible] esting (R=-0.36,

p<0.001, *q*=0.035). The lower the serum levels of ESC, the greater the intensity of sexual side effects that is reported. The ESC+ARI treatment arm did not show this effect. *Post-hoc* analyses revealed this effect was driven by a significant correlation between serum ESC_j levels and intensity of anorgasmia (R=-0.32, *p*=0.007) and decreased libido (R=-0.25, *p*=0.038).
ESC = Escitalopram; S-DCT = S-desmethylcitalopram.
**p*<0.05; ***p*<0.01

Figure S21. Spearman correlation between concentrations of ARI and metabolite-to-drug ratio and symptom improvement from baseline at Week 16.

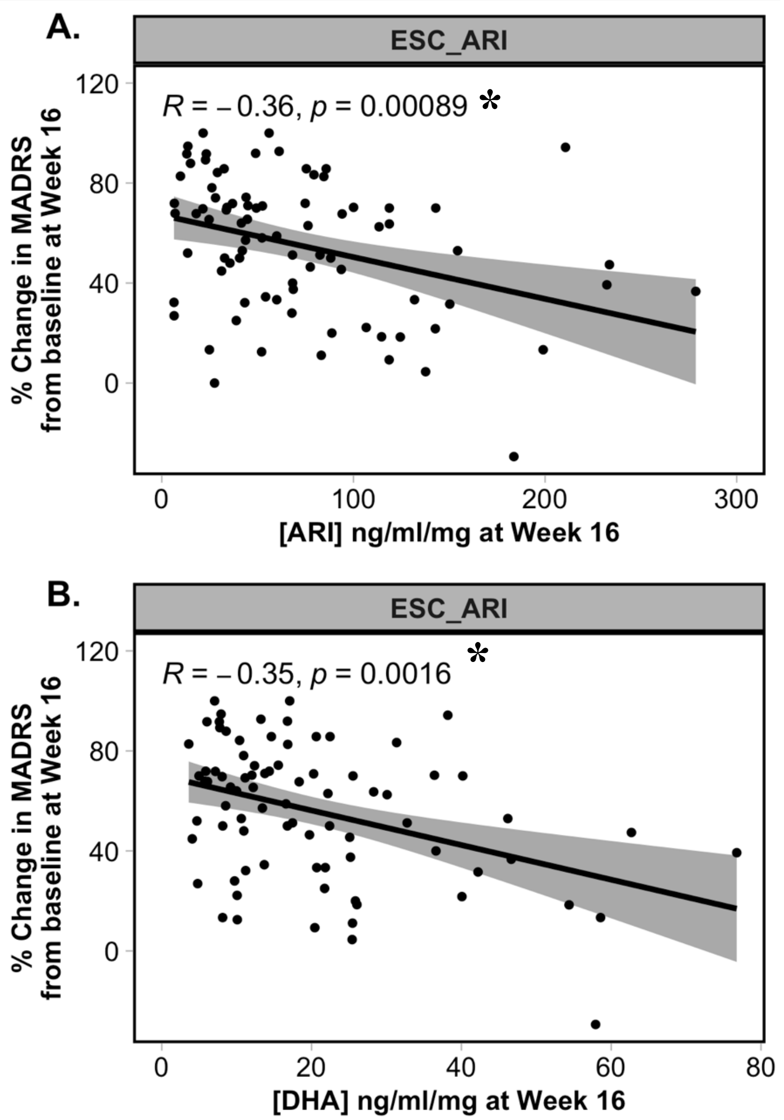

During Phase II, in the ESC+ARI treatment arm, unadjusted serum **(A)** ARI and (**B**) DHA concentrations were significantly correlated percent change in MADRS from baseline at Week 16, following correcting for multiple testing (R=-0.36, $p<0.001$, $q=0.048$; R=-0.35, $p<0.001$, $q=0.048$, respectively). The higher the concentration of ARI and DHA in this treatment arm, the lower the percentage symptom improvement at Week 16.
ARI = Aripiprazole; DHA = Dehydroaripiprazole.
*$p<0.05$

Figure S22. Summary of the mediation analysis with dose-adjusted escitalopram serum concentrations at Week 16 as mediator of the relationship between *CYP2C19* and *CYP2C6* metabolizer groups and total symptom improvement in the ESC-Only treatment arm.

A.

	Model 1			Model 2			Model 3		
	**B**	**CI**	***p***	**B**	**CI**	***p***	**B**	**CI**	***p***
(Intercept)	83.93	[74.91, 92.95]	**<0.001 *****	1.22	[0.88, 1.56]	**<0.001 *****	92.13	[80.37, 103.89]	**<0.001 *****
CYP2C19 IM+PM	-14.97	[-27.08, -2.85]	**0.016 ***	1.00	[0.54, 1.46]	**<0.001 *****	-8.26	[-21.69, 5.17]	0.224
CYP2C19 RM+UM	-3.14	[-15.41, 9.13]	0.611	-0.10	[-0.56, 0.37]	0.678	-3.79	[-15.77, 8.19]	0.530
CYP2D6 IM+PM	-1.86	[-11.97, 8.26]	0.715	0.82	[0.43, 1.20]	**<0.001 *****	3.63	[-7.54, 14.79]	0.519
ESC_{adj} levels at Week 16							-6.70	[-13.08, -0.32]	**0.040 ***
N	69			69			69		
R2	0.09			0.41			0.15		

B.

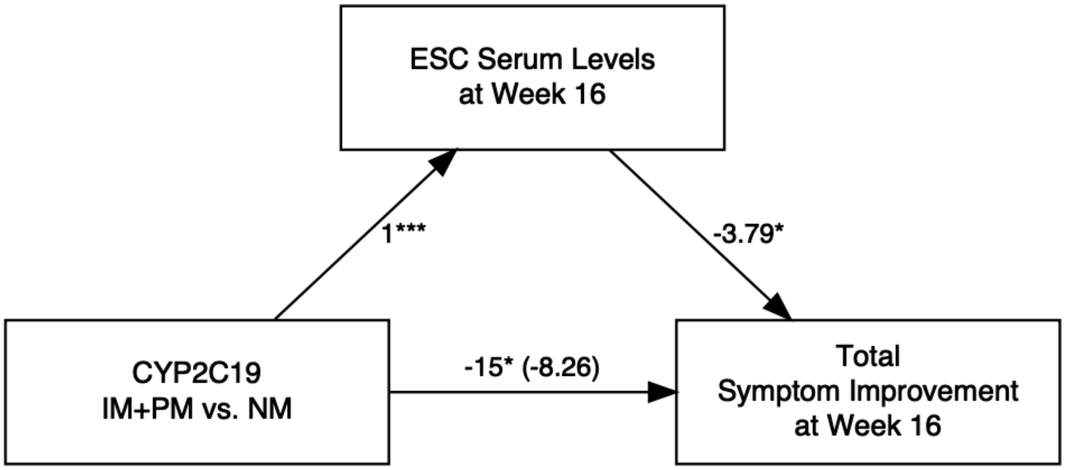

C.

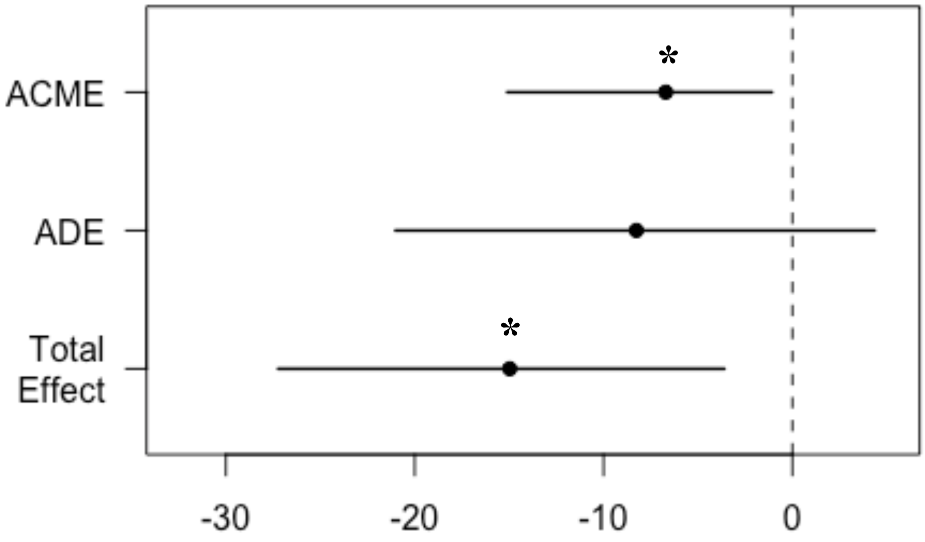

	Estimate	95% CI Lower	95% CI Upper	p-value
ACME	-6.71	-15.00	-0.98	0.020 *
ADE	-8.26	-20.97	4.64	0.193
Total Effect	-14.97	-27.16	-3.05	0.014 *
Prop. Mediated	0.45	0.05	1.71	0.033 *

The three linear regression models performed are shown in **(A)**. Model 1 revealed that *CYP2C19* IM+PMs showed lower symptom improvement at Week 16 compared to NMs. Model 2 revealed that *CYP2C19* IM+PMs also showed higher ESC_{adj} serum levels at W16 compared to NMs. In Model 3, relationship between *CYP2C19* metabolizer group and total symptom improvement after controlling for ESC_{adj} serum concentrations was not significant, while ESC serum concentrations were significantly associated with total symptom improvement at Week 16. Effects are indicated by regression coefficients in overall mediation model shown in **(B)**, where indirect effect of *CYP2C19* metabolizer group on total symptom improvement is shown in parenthesis. Results of the nonparametric bootstrap analyses with 5000 simulations **(C)** estimated a percent mediation of 45% indicating that about half of the effect of *CYP2C19* IM+PM phenotype on symptom improvement may be mediated by ESC serum levels.

ACME=average causal mediation effects, ADE=average direct effect, ESC_{adj} = escitalopram adjusted for dosage, IM=intermediate metabolizer; NM=normal metabolizers; PM=poor metabolizer; RM=rapid metabolizer; UM=ultra-rapid metabolizer.

***$p < 0.001$; **$p < 0.01$; *$p < 0.05$.

Figure S23. The relationship between total symptom improvement and serum levels of ESC, corrected for dosage, at trial end by *CYP2C19* metabolizer phenotypes for each treatment arm.

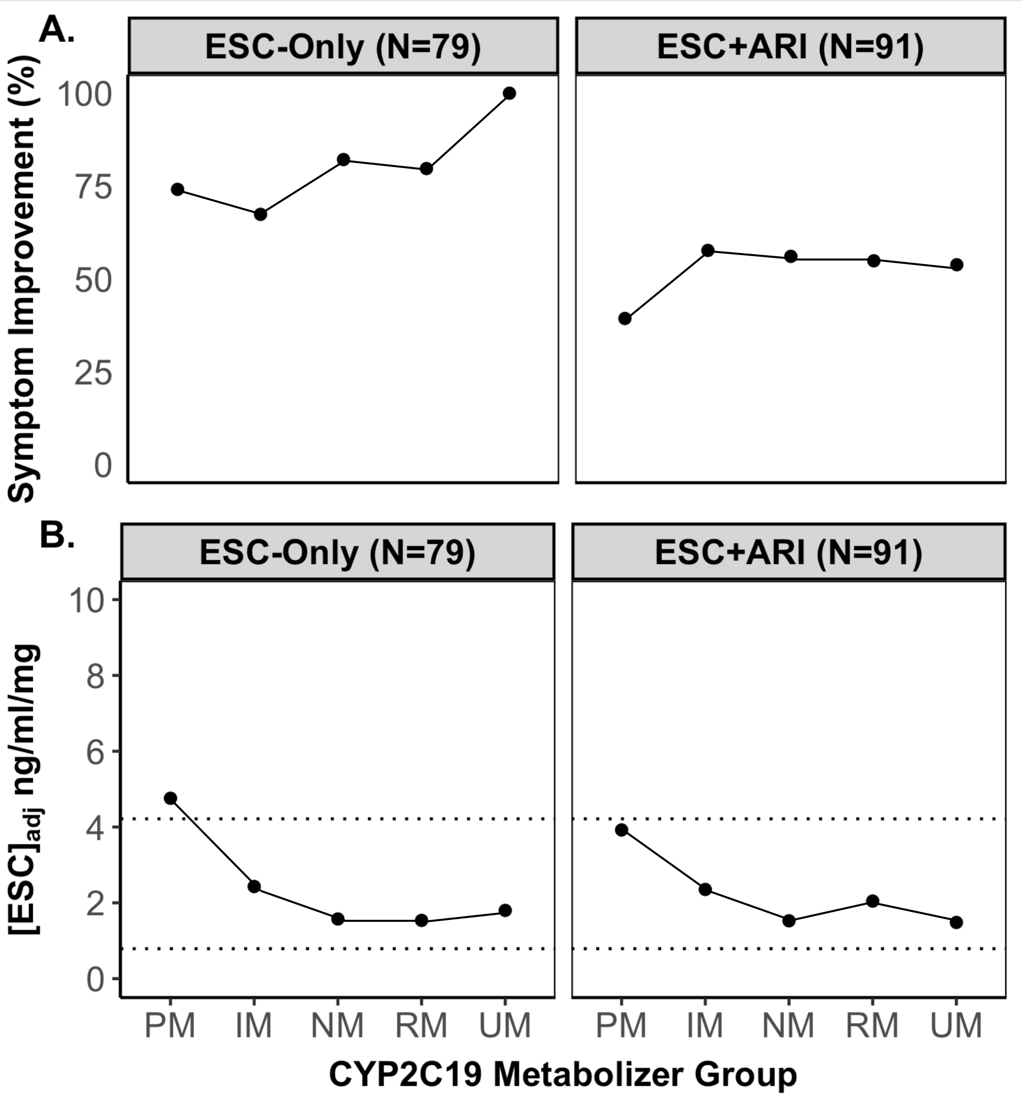

Those with slower CYP2C19 enzymatic capacity demonstrate **(A)** lower total symptom improvement and **(B)** higher ESC_{adj} serum levels at trial end in the ESC-Only treatment arm. In ESC+ARI, *CYP2C19* PMs demonstrate the **(A)** lowest symptom improvement and **(B)** highest ESC serum concentrations; however, symptom improvement between IMs, NMs, RMs, and UMs do not significantly differ.
The dotted line represents the therapeutic range, which is the serum concentration usually expected to achieve the desired therapeutic effect.
ESC_{adj} = escitalopram adjusted for dosage, IM=intermediate metabolizer; NM=normal metabolizers; PM=poor metabolizer; RM=rapid metabolizer; UM=ultra-rapid metabolizer.
